# A Randomized, Double-blind, Multicenter Trial Comparing Efficacy and Safety of Imipenem/Cilastatin/Relebactam Versus Piperacillin/Tazobactam in Adults With Hospital-acquired or Ventilator-associated Bacterial Pneumonia (RESTORE-IMI 2 Study)

**DOI:** 10.1093/cid/ciaa803

**Published:** 2020-08-12

**Authors:** Ivan Titov, Richard G Wunderink, Antoine Roquilly, Daniel Rodríguez Gonzalez, Aileen David-Wang, Helen W Boucher, Keith S Kaye, Maria C Losada, Jiejun Du, Robert Tipping, Matthew L Rizk, Munjal Patel, Michelle L Brown, Katherine Young, Nicholas A Kartsonis, Joan R Butterton, Amanda Paschke, Luke F Chen

**Affiliations:** 1 Department of Anesthesiology and Intensive Care, Ivano-Frankivsk Regional Clinical Hospital, Ivano-Frankivsk, Ukraine; 2 Department of Medicine, Division of Pulmonary and Critical Care, Northwestern University Feinberg School of Medicine, Chicago, Illinois, USA; 3 EA3826 Thérapeutiques Anti-Infectieuses, Institut de Recherche en Santé 2 Nantes Biotech, Université, de Nantes, Nantes, France; 4 Department of Intensive Care, Hospital Civil de Guadalajara, Guadalajara, Mexico; 5 Department of Medicine & Philippine General Hospital, Division of Pulmonary Medicine, University of the Philippines, Manila, Philippines; 6 Division of Geographic Medicine and Infectious Diseases, Tufts Medical Center, Boston, Massachusetts, USA; 7 Department of Internal Medicine, Division of Infectious Diseases, University of Michigan Medical School, Ann Arbor, Michigan, USA; 8 Merck Research Laboratories, Merck & Co, Inc, Kenilworth, New Jersey, USA

**Keywords:** carbapenem resistant, KPC, *Pseudomonas*, nosocomial pneumonia, mechanical ventilation

## Abstract

**Background:**

Imipenem combined with the β-lactamase inhibitor relebactam has broad antibacterial activity, including against carbapenem-resistant gram-negative pathogens. We evaluated efficacy and safety of imipenem/cilastatin/relebactam in treating hospital-acquired/ventilator-associated bacterial pneumonia (HABP/VABP).

**Methods:**

This was a randomized, controlled, double-blind phase 3 trial. Adults with HABP/VABP were randomized 1:1 to imipenem/cilastatin/relebactam 500 mg/500 mg/250 mg or piperacillin/tazobactam 4 g/500 mg, intravenously every 6 hours for 7–14 days. The primary endpoint was day 28 all-cause mortality in the modified intent-to-treat (MITT) population (patients who received study therapy, excluding those with only gram-positive cocci at baseline). The key secondary endpoint was clinical response 7–14 days after completing therapy in the MITT population.

**Results:**

Of 537 randomized patients (from 113 hospitals in 27 countries), the MITT population comprised 264 imipenem/cilastatin/relebactam and 267 piperacillin/tazobactam patients; 48.6% had ventilated HABP/VABP, 47.5% APACHE II score ≥15, 24.7% moderate/severe renal impairment, 42.9% were ≥65 years old, and 66.1% were in the intensive care unit. The most common baseline pathogens were *Klebsiella pneumoniae* (25.6%) and *Pseudomonas aeruginosa* (18.9%). Imipenem/cilastatin/relebactam was noninferior (*P < *.001) to piperacillin/tazobactam for both endpoints: day 28 all-cause mortality was 15.9% with imipenem/cilastatin/relebactam and 21.3% with piperacillin/tazobactam (difference, −5.3% [95% confidence interval {CI}, −11.9% to 1.2%]), and favorable clinical response at early follow-up was 61.0% and 55.8%, respectively (difference, 5.0% [95% CI, −3.2% to 13.2%]). Serious adverse events (AEs) occurred in 26.7% of imipenem/cilastatin/relebactam and 32.0% of piperacillin/tazobactam patients; AEs leading to treatment discontinuation in 5.6% and 8.2%, respectively; and drug-related AEs (none fatal) in 11.7% and 9.7%, respectively.

**Conclusions:**

Imipenem/cilastatin/relebactam is an appropriate treatment option for gram-negative HABP/VABP, including in critically ill, high-risk patients.

**Clinical Trials Registration:**

NCT02493764.

## Teaser

Imipenem/cilastatin/relebactam was noninferior to piperacillin/tazobactam for treating hospital-acquired and ventilator-associated bacterial pneumonia (HABP/VABP), with a comparable tolerability profile. Imipenem/cilastatin/relebactam is an efficacious treatment option for nosocomial pneumonia, including HABP/VABP in mechanically ventilated and critically ill, high-risk patients.

**(See the Editorial Commentary by Kollef and Micek on pages e4549–51.)**

Hospital-acquired bacterial pneumonia (HABP) and ventilator-associated bacterial pneumonia (VABP) are common nosocomial infections [1–3] associated with high mortality rates (~20%–50%). Mortality is highest in ventilated HABP, followed by VABP, and lowest in nonventilated HABP [4, 5]. In high-risk populations (eg, critically ill, mechanically ventilated, and/or immunocompromised patients), rapid initiation of appropriate antibacterial therapy is crucial to improve survival [6]. Mortality worsens in HABP/VABP caused by antibacterial-resistant pathogens, for example, multidrug-resistant *Pseudomonas aeruginosa* and extended-spectrum β-lactamase (ESBL)-producing Enterobacterales [7]. The incidence of multidrug-resistant gram-negative pathogens, such as carbapenem-resistant Enterobacterales (CRE) and *Pseudomonas aeruginosa*, is increasing worldwide [8–10]. Since carbapenems are cornerstones of HABP/VABP therapy [3, 4], new treatment options are needed.

Carbapenem nonsusceptibility can be overcome by combining carbapenems with suitable β-lactamase inhibitors (BLIs). The novel, small-molecule BLI relebactam (REL) inhibits class A carbapenemases (eg, *Klebsiella pneumoniae* carbapenemase [KPC]) and class C cephalosporinases (eg, AmpC) [11], which commonly contribute to carbapenem nonsusceptibility. Given their complementary pharmacokinetic/pharmacodynamic (PK/PD) profiles, REL is particularly suitable for combination with imipenem/cilastatin (IMI), a well-established carbapenem coadministered with the renal dehydropeptidase inhibitor cilastatin [12, 13]. Imipenem plus REL has broad antibacterial activity, including many strains of CRE and carbapenem-nonsusceptible *P. aeruginosa* [14–18]. The combination of imipenem/cilastatin with REL exhibits good intrapulmonary penetration [12]. Preclinical studies, dose-ranging phase 1 and 2 trials, and population PK analyses support 500 mg/ 500 mg IMI with 250 mg REL every 6 hours as a suitable dosing regimen for HABP/VABP treatment [12, 13, 19–23]. This dose also showed efficacy in a phase 3 trial assessing IMI/REL for treating IMI-nonsusceptible infections, including HABP/VABP [24]. We conducted a large randomized controlled trial evaluating efficacy and safety of IMI/REL vs piperacillin/tazobactam (PIP/TAZ) for treatment of HABP/VABP.

## METHODS

### Study Design

RESTORE-IMI 2 (Protocol number MK-7655A-014) was a phase 3, randomized, double-blind, noninferiority trial evaluating IMI/REL vs PIP/TAZ for HABP/VABP. The study was conducted in accordance with principles of Good Clinical Practice and approved by the appropriate institutional review boards and regulatory agencies. The trial is registered at ClinicalTrials.gov (NCT02493764).

### Patients

Eligible patients were ≥18 years old and required intravenous antibacterial therapy for nonventilated HABP, ventilated HABP, or VABP. An adequate baseline lower respiratory tract (LRT) specimen was required within 48 hours of screening. Patients needed to fulfill 3 diagnostic criteria, with an onset of 48 hours after starting mechanical ventilation for VABP; or of either 48 hours after hospitalization or within 7 days of hospital discharge for HABP: (1) ≥1 clinical feature: new onset or worsening pulmonary signs/symptoms (eg, cough, dyspnea, tachypnea, need for mechnical ventilation); hypoxemia; need for acute ventilator support system changes to enhance oxygenation; and/or new onset of suctioned respiratory secretions; (2) ≥1 of the following signs: fever; hypothermia; total peripheral white blood cell count (WBC) ≥10 000 cells/μL; leukopenia (total WBC count ≤4500 cells/μL); and/or >15% immature neutrophils; and (3) chest radiograph showing ≥1 new/progressive infiltrate suggestive of bacterial pneumonia [25].

Patients with >24 hours of effective antibacterial therapy for the current HABP/VABP episode within 72 hours prior to randomization were not eligible, unless they failed this prior therapy (ie, persistent/worsening signs/symptoms of HABP/VABP despite >48 hours on the prior regimen). Other important exclusion criteria were as follows: baseline LRT specimen showed only gram-positive cocci; creatinine clearance <15 mL/minute or need for dialysis; confirmed/suspected community-acquired, viral, fungal, or parasitic pneumonia; HABP/VABP caused by any airway obstructive process, including lung cancer; immunodeficiency/active immunosuppression; expected survival <72 hours; concurrent condition (eg, tuberculosis, cystic fibrosis, or endocarditis) potentially precluding evaluation of therapeutic response; and anticipated need for specific medications, including nonstudy systemic antibacterial agents, valproate, selective serotonin reuptake inhibitors, serotonin-norepinephrine reuptake inhibitors, or monoamine oxidase inhibitors. See the Supplementary Appendix for the full study protocol and inclusion/exclusion criteria.

### Randomization and Masking

After a ≤48-hour screening period, eligible patients were randomized (stratified by nonventilated HABP vs ventilated HABP/VABP and by Acute Physiology and Chronic Health Evaluation II [APACHE II] score <15 vs ≥15; block size = 4) via a centralized, interactive voice/integrated web response system in a 1:1 ratio to either IMI/REL 500 mg/500 mg/250 mg or PIP/TAZ 4 g/500 mg. Patients and all investigational staff remained blinded to treatment assignments throughout. Unblinded study pharmacists prepared the infusions, masking infusion bags with opaque sleeves.

### Procedures

Both IMI/REL and PIP/TAZ were dose-adjusted based on renal function (see Supplementary Appendix for details; creatinine clearance was estimated using the Cockcroft-Gault equation) and administered every 6 hours as 30-minute intravenous infusions. Treatment duration was 7–14 days; a 14-day duration was required with HABP/VABP due to *P. aeruginosa* or concurrent bacteremia. All patients received empiric intravenous linezolid (600 mg every 12 hours) until baseline respiratory cultures confirmed the absence of methicillin-resistant *Staphylococcus aureus* (MRSA); if MRSA was present, linezolid was continued for ≥7 days total (≥14 days with MRSA bacteremia). Adjunctive gram-negative therapy and other concomitant nonstudy systemic antibacterial agents were prohibited.

A full assessment schedule is in the protocol shown in the Supplementary Appendix. Study visits were performed on day 1 (randomization); days 3, 6, and 10 (if applicable); and at end of therapy (EOT). Following study therapy completion, patients were evaluated at an early follow-up visit (EFU) 7–14 days post-EOT and on day 28 post-randomization. Clinical HABP/VABP signs/symptoms, respiratory parameters, and adverse events (AEs) were assessed daily during intravenous therapy and at EOT, EFU, and day 28. Chest radiographs were obtained on day 1 (if not obtained within ≤48 hours of randomization) and at EOT, EFU, and day 28.

LRT samples for Gram stain, microbiologic culture, and susceptibility testing were obtained at EOT, EFU, and other visits as clinically indicated. Blood cultures were to be collected on day 1 and, if positive, repeated until achieving 2 negative consecutive cultures. Pathogen identification and susceptibility were confirmed at a central laboratory using standard broth dilution methodology and current Clinical and Laboratory Standards Institute breakpoints [26, 27]. Intermediate-susceptible pathogens were classified as nonsusceptible.

### Outcomes

The primary efficacy population was the modified intent-to-treat (MITT) population, that is, all randomized patients with ≥1 dose of study treatment and whose baseline Gram stain did not show only gram-positive cocci. The microbiologic MITT (mMITT) population comprised MITT patients with ≥1 baseline LRT pathogen species against which imipenem plus REL is known to have antibacterial activity. The clinically evaluable (CE) population corresponded to MITT patients who met diagnostic criteria for HABP/VABP, had no major protocol violations, received the minimum therapy duration, and had a corresponding efficacy assessment. The safety population comprised all randomized patients with ≥1 dose of assigned study treatment.

The primary efficacy endpoint was day 28 all-cause mortality (ACM). The key secondary endpoint was favorable clinical response at EFU, both in the MITT population. Other secondary endpoints were day 28 mortality (mMITT population), microbiologic response at EOT and EFU (mMITT population), and clinical response at EFU (CE population). Clinical response was categorized as overall favorable (resolution of baseline HABP/VABP signs/symptoms and no nonstudy antibacterial therapy for HABP/VABP), overall unfavorable (persistence, progression, or insufficient improvement of baseline HABP/VABP signs/symptoms; patient discontinued study therapy due to lack of efficacy; or death due to the index HABP/VABP infection), or indeterminate (data not available for any reason, including when a patient died from causes not attributable to HABP/VABP). Overall microbiologic response was categorized as eradication (LRT culture showing absence of baseline pathogen), presumed eradication (LRT culture unavailable because of clinical cure), persistence (LRT culture growing the baseline pathogen), presumed persistence (patient discontinued study therapy due to unfavorable clinical response), or indeterminate (any circumstances, including incomplete data, precluding characterization of microbiologic outcome); eradication and presumed eradication were regarded as favorable responses. Indeterminate clinical and microbiologic responses were treated as failures for MITT and mMITT analyses and excluded from the CE analysis.

### Statistical Analysis

This trial evaluated the noninferiority of IMI/REL to PIP/TAZ in the primary and key secondary endpoints, which were compared using the stratified Miettinen and Nurminen method [28]. Noninferiority in the primary endpoint was achieved if the upper bound of the 2-sided 95% confidence interval (CI) for the adjusted treatment difference (IMI/REL minus PIP/TAZ) was <10%. Noninferiority in the key secondary endpoint was assumed if the lower bound of the 2-sided 95% CI for the adjusted treatment difference was greater than −12.5%. The planned sample size of 268 patients per arm provided 90% power to reject the null hypothesis that the true difference exceeded the noninferiority margin of the respective endpoint for the primary endpoint and 84% power for the key secondary endpoint, at a 1-tailed α of 2.5%. Safety data were analyzed descriptively. All statistical analyses were conducted using SAS version 9.4 software (SAS Institute, Cary, North Carolina).

## RESULTS

### Patients

**Figure 1.**
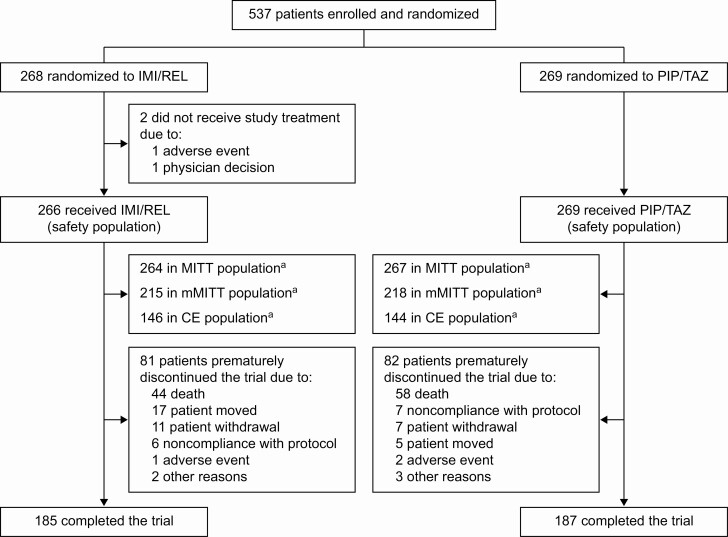
Study analysis population flowchart. ^a^Reasons for exclusion from this analysis population are listed in Supplementary Table 1. Abbreviations: CE, clinically evaluable; IMI/REL, imipenem/cilastatin with relebactam; MITT, modified intent-to-treat; mMITT, microbiologic modified intent-to-treat; PIP/TAZ, piperacillin/tazobactam.

Patients were randomized at 113 hospitals from 27 countries between January 2016 and April 2019. Of 537 randomized patients, 535 (266 IMI/REL, 269 PIP/TAZ) received ≥1 dose of study treatment, and 531 (264 IMI/REL, 267 PIP/TAZ) were included in the MITT population (Figure 1 and Supplementary Table 1). Baseline demographic and clinical characteristics were similar between treatment arms (Table 1); 66.1% of MITT patients were in the intensive care unit (ICU), 47.5% had APACHE II score ≥15, 48.6% had ventilated HABP/VABP, 42.9% were >65 years old, 16.6% had augmented renal clearance (creatinine clearance of ≥150 mL/minute), and 24.7% had moderate/severe renal impairment. Overall, 45.2% of patients received ≥1 dose of systemic antibacterials within 72 hours prior to study therapy (Supplementary Table 2); the use of systemic antibacterial agents with gram-negative activity within 72 hours prior to first dose of study drug was slightly higher in the PIP/TAZ (49.1%) than the IMI/REL arm (41.3%) (Table 1).

**Table 1.** Baseline (at Randomization) Demographics and Clinical Characteristics in the Modified Intent-to-Treat Population

Characteristic	IMI/REL (n = 264)	PIP/TAZ (n = 267)	Total (N = 531)
Sex			
Female	86 (32.6)	78 (29.2)	164 (30.9)
Male	178 (67.4)	189 (70.8)	367 (69.1)
Age, y			
<65	151 (57.2)	152 (56.9)	303 (57.1)
≥65	113 (42.8)	115 (43.1)	228 (42.9)
Mean (SD)	60.5 (16.9)	58.8 (18.4)	59.6 (17.7)
Median (range)	62.0 (18–96)	62.0 (18–98)	62.0 (18–98)
Geographic region			
Americas	59 (22.3)	71 (26.6)	130 (24.5)
United States	5 (1.9)	15 (5.6)	20 (3.8)
Europe	166 (62.9)	160 (59.9)	326 (61.4)
Asia and Australia	39 (14.8)	36 (13.5)	75 (14.1)
Weight, kg, median (range)	75.0 (26.8–150.5)	78.0 (27.7–145.0)	76.2 (26.8–150.5)
BMI, kg/m^2^, median (range)	25.9 (12.6–52.1)	25.6 (13.7–62.8)	25.7 (12.6–62.8)
Creatinine clearance, mL/min^a^			
≥150 (augmented renal clearance)	38 (14.4)	50 (18.7)	88 (16.6)
≥90 to <150 (normal renal function)	103 (39.0)	85 (31.8)	188 (35.4)
<90 to ≥60 (mild renal impairment)	52 (19.7)	72 (27.0)	124 (23.4)
<60 to ≥30 (moderate renal impairment)	61 (23.1)	48 (18.0)	109 (20.5)
<30 to ≥15 (severe renal impairment)	10 (3.8)	12 (4.5)	22 (4.1)
Elevated hepatic enzymes^b^			
Yes	71 (26.9)	91 (34.1)	162 (30.5)
No	178 (67.4)	161 (60.3)	339 (63.8)
Missing	15 (5.7)	15 (5.6)	30 (5.6)
In the ICU			
Yes	175 (66.3)	176 (65.9)	351 (66.1)
No	89 (33.7)	91 (34.1)	180 (33.9)
APACHE II score			
<15	139 (52.7)	140 (52.4)	279 (52.5)
≥15	125 (47.3)	127 (47.6)	252 (47.5)
Mean (SD)	14.6 (6.2)	14.8 (6.7)	14.7 (6.4)
Median (range)	14.0 (2–31)	14.0 (1–37)	14.0 (1–37)
Primary diagnosis			
Nonventilated HABP	142 (53.8)	131 (49.1)	273 (51.4)
Ventilated HABP/VABP	122 (46.2)	136 (50.9)	258 (48.6)
Ventilated HABP	31 (11.7)	35 (13.1)	66 (12.4)
VABP	91 (34.5)	101 (37.8)	192 (36.2)
CPIS			
<6	114 (43.2)	95 (35.6)	209 (39.4)
≥6	150 (56.8)	172 (64.4)	322 (60.6)
Mean (SD)	5.9 (1.8)	6.1 (1.8)	6.0 (1.8)
Median (range)	6.0 (1–10)	6.0 (1–10)	6.0 (1–10)
Duration of prior hospitalization, d			
Mean (SD)	30.4 (126.1)	31.1 (143.0)	30.7 (134.7)
Median (range)	8.0 (1–1169)	7.0 (1–1338)	8.0 (1–1338)
Missing	0	1 (0.4)	1 (0.2)
Received systemic antibacterial with gram-negative activity within 72 h prior to first dose			
No	155 (58.7)	136 (50.9)	291 (54.8)
Yes (≤24 h)	54 (20.5)	68 (25.5)	122 (23.0)
Yes (>24 to ≤72 h)	55 (20.8)	63 (23.6)	118 (22.2)
Concurrent bacteremia			
Yes (with any pathogen)	15 (5.7)	16 (6.0)	31 (5.8)
Yes (with baseline LRT pathogen)	5 (1.9)	7 (2.6)	12 (2.3)
No	249 (94.3)	251 (94.0)	500 (94.2)
No. of baseline LRT pathogens			
Monomicrobial	160 (60.6)	160 (59.9)	320 (60.3)
Polymicrobial	55 (20.8)	58 (21.7)	113 (21.3)
None	49 (18.6)	49 (18.4)	98 (18.5)
Baseline LRT pathogen (≥10% in either treatment arm)^c^	(n = 215)	(n = 218)	(N = 433)
*Klebsiella pneumoniae*	58 (27.0)	53 (24.3)	111 (25.6)
*Pseudomonas aeruginosa*	34 (15.8)	48 (22.0)	82 (18.9)
*Acinetobacter calcoaceticus-baumannii* complex	32 (14.9)	36 (16.5)	68 (15.7)
*Escherichia coli*	30 (14.0)	37 (17.0)	67 (15.5)
MSSA	23 (10.7)	22 (10.1)	45 (10.4)

Data are presented as no. (%) unless otherwise indicated.

Abbreviations: APACHE II, Acute Physiology and Chronic Health Evaluation II; CPIS, Clinical Pulmonary Infection Score; HABP, hospital-acquired bacterial pneumonia; ICU, intensive care unit; IMI/REL, imipenem/cilastatin with relebactam; LRT, lower respiratory tract; MSSA, methicilin-susceptible *Staphylococcus aureus*; PIP/TAZ, piperacillin/tazobactam; SD, standard deviation; VABP, ventilator-associated bacterial pneumonia.

^a^Creatinine clearance was estimated using the Cockcroft-Gault equation.

^b^Defined as either alanine aminotransferase or aspartate aminotransferase being greater than the upper limit of normal at randomization.

^c^Baseline pathogens were assessed in the microbiologic modified intent-to-treat population.

Baseline LRT pathogens were similar between arms (Table 1); most frequent were *K. pneumoniae* (25.6%), *P. aeruginosa* (18.9%), *Acinetobacter calcoaceticus-baumannii* complex (15.7%), and *Escherichia coli* (15.5%). Among MITT patients with ≥1 identified baseline LRT pathogen and susceptibility interpretation available, 149 of 187 (79.7%) of IMI/REL and 127 of 193 of PIP/TAZ (65.8%) patients had all those baseline pathogens with susceptibility interpretations susceptible to randomized study therapy. Baseline concurrent bacteremia with any pathogen was reported for 5.7% of IMI/REL and 6.0% of PIP/TAZ patients.

In the IMI/REL arm, 209 of 266 (78.6%) MITT patients completed study therapy, vs 187 of 269 (69.5%) receiving PIP/TAZ. Mean treatment duration was 8.7 days with IMI/REL and 8.3 days with PIP/TAZ; median duration was 6.8 (range, 0–14) days in both arms (Supplementary Table 3). Concomitant, nonstudy, systemic antibacterial agents with gram-negative activity were administered, in violation of the study protocol, to 21 of 264 (8.0%) IMI/REL and 28 of 267 (10.5%) PIP/TAZ patients (Supplementary Table 4).

### Efficacy

**Figure 2.**
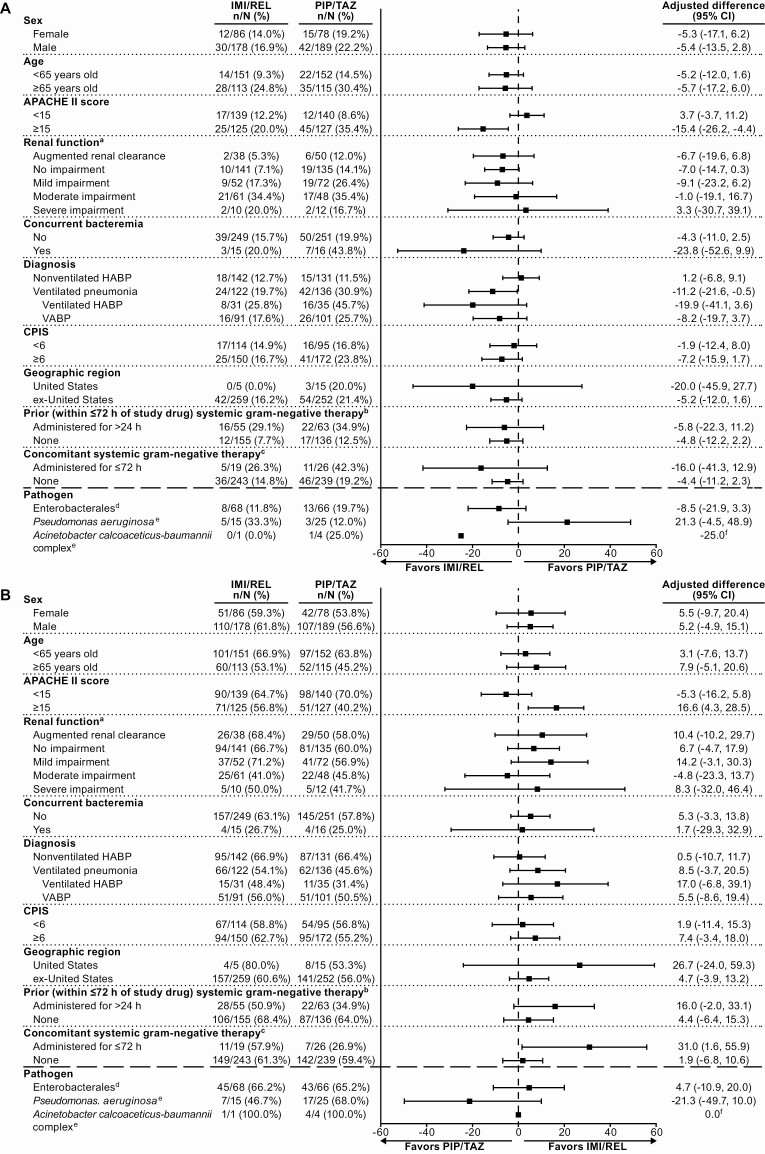
Primary and key secondary efficacy endpoints in clinically relevant patient subgroups of the modified intent-to-treat (MITT) population: 28-day all-cause mortality (*A*) and favorable clinical response (*B*). (Note: Per-pathogen outcomes are shown for microbiologic modified intent-to-treat [mMITT] patients with all baseline lower respiratory tract [LRT] isolates susceptible to both study drugs.) ^a^Post hoc analysis; all other subgroups were prospectively defined. ^b^Outcomes in patients who received <24 hours of prior, systemic, gram-negative therapy (applicable to 20.5% of imipenem/cilastatin with relebactam [IMI/REL] and 25.5% of piperacillin/tazobactam [PIP/TAZ] patients) are not shown. ^c^Two patients in each treatment arm received >72 hours of concomitant, systemic, gram-negative therapy; outcomes in this very small subpopulation are not shown. ^d^Outcomes are shown for the subpopulation of mMITT patients with only Enterobacterales (of any species) baseline LRT isolates and all baseline isolates susceptible to both IMI/REL and PIP/TAZ. ^e^Outcomes are shown for the subpopulation of mMITT patients with ≥1 baseline LRT isolate of this pathogen and all baseline isolates susceptible to both IMI/REL and PIP/TAZ. ^f^CIs were not calculated due to the low sample size (<5 patients in both arms) of this subpopulation. Abbreviations: APACHE II, Acute Physiology and Chronic Health Evaluation II; CI, confidence interval; CPIS, Clinical Pulmonary Infection Score; HABP, hospital-acquired bacterial pneumonia; IMI/REL, imipenem/cilastatin with relebactam; PIP/TAZ, piperacillin/tazobactam; VABP, ventilator-associated bacterial pneumonia.

IMI/REL was noninferior to PIP/TAZ for the primary endpoint of day 28 ACM: 15.9% with IMI/REL and 21.3% with PIP/TAZ (adjusted treatment difference, −5.3% [95% CI, −11.9% to 1.2%]; noninferiority *P* <.001). In the subgroup of patients with a primary diagnosis of ventilated HABP/VABP as well as in the subgroup of patients with APACHE II scores ≥15, mortality was lower with IMI/REL than PIP/TAZ, and the 95% CI for the difference excluded 0 (Figure 2A and Supplementary Table 5). Mortality rates in other patient subpopulations were comparable between treatment arms.

IMI/REL was also noninferior to PIP/TAZ for the key secondary endpoint of favorable clinical response: 61.0% with IMI/REL and 55.8% with PIP/TAZ (adjusted treatment difference, 5.0% [95% CI, −3.2% to 13.2%]; noninferiority *P* <.001). The incidence of relapse/clinical failure was comparable between IMI/REL (38/264 [14.4%]) and PIP/TAZ (32/267 [12.0%]). Favorable clinical response rates at EFU were comparable between treatment arms across all evaluated clinically relevant subgroups, except patients with APACHE II scores ≥15, where response rates were greater with IMI/REL (Figure 2B and Supplementary Table 6). In the other secondary endpoints, including overall microbiologic response at EFU, outcomes were comparable between treatments (Table 2 and Supplementary Table 7). Day 28 ACM in mMITT patients with a primary diagnosis of ventilated HABP or VABP showed the same pattern as in the primary efficacy population: mortality rates were lower for IMI/REL (19/102 [18.6%]) than PIP/TAZ (33/107 [30.8%]), with a difference of −12.2% (95% CI, −23.7% to −.5%).

**Table 2.** Primary, Key Secondary, and Other Prespecified Secondary Efficacy Endpoints

Endpoint	IMI/REL, no./No. (%)^a^	PIP/TAZ, no./No. (%)^a^	Adjusted Difference^b^, % (95% CI)
Primary endpoint			
Day 28 all-cause mortality (MITT)	42/264 (15.9)	57/267 (21.3)	−5.3 (−11.9 to 1.2)^c^
Key secondary endpoint			
Favorable clinical response at EFU (MITT)	161/264 (61.0)^d^	149/267 (55.8)^d^	5.0 (−3.2 to 13.2)^e^
Other secondary endpoints			
Day 28 all-cause mortality (mMITT)	36/215 (16.7)	44/218 (20.2)	−3.5 (−10.9 to 3.6)
Favorable microbiologic response at EFU (mMITT)	146/215 (67.9)^d^	135/218 (61.9)^d^	6.2 (−2.7 to 15.0)
Favorable clinical response at EFU (CE)	101/136 (74.3)	100/126 (79.4)	−3.7 (−13.6 to 6.4)

Abbreviations: CE, clinically evaluable population; CI, confidence interval; EFU, early follow-up visit; IMI/REL, imipenem/cilastatin with relebactam; MITT, modified intent-to-treat population; mMITT, microbiologic modified intent-to-treat population; PIP/TAZ, piperacillin/tazobactam.

^a^no./No. indicates number of patients who died or had unknown survival status (for mortality endpoints) or number of patients with favorable response (for response endpoints)/ total number of patients in the particular analysis population and treatment arm.

^b^Adjusted differences and CIs stratified by pneumonia type (nonventilated hospital-acquired bacterial pneumonia [HABP] vs ventilated HABP/ventilator-associated bacterial pneumonia) and by baseline Acute Physiology and Chronic Health Evaluation II score (<15 vs ≥15) using the Miettinen and Nurminen method [28].

^c^The upper bound of the CI is less than the predefined noninferiority margin of 10 percentage points, indicating success for the noninferiority hypothesis.

^d^A breakdown of reasons for unfavorable response by treatment arm is shown in Supplementary Table 7.

^e^The lower bound of the CI is greater than the predefined noninferiority margin of −12.5 percentage points, indicating success for the noninferiority hypothesis.

Baseline pathogen susceptibility to study drugs did not impact primary and key secondary efficacy outcomes (Supplementary Table 8). Indeed, in a post hoc mMITT analysis of patients who had all baseline pathogens susceptible to both IMI/REL and PIP/TAZ, per-pathogen microbiologic eradication rates at EOT were comparable between both treatment arms for Enterobacterales, *P. aeruginosa*, and *A. calcoaceticus-baumannii* complex (Supplementary Table 9). The corresponding per-pathogen day 28 ACM and favorable clinical response rates are shown in Figure 2.

### Safety

Most patients (85.0% IMI/REL, 86.6% PIP/TAZ) had ≥1 AE, but few AEs were classified as drug-related by the investigator (Table 3). The incidence of specific drug-related AEs (Supplementary Table 10) was generally similar between treatment arms; most commonly reported with IMI/REL were diarrhea, increased aspartate aminotransferase, and increased alanine aminotransferase, each with an incidence of 2.3%. Six patients (2.3%) in the IMI/REL arm and 4 (1.5%) in the PIP/TAZ arm discontinued treatment due to drug-related AEs (Table 3). Serious drug-related AEs were reported in 1.1% of IMI/REL vs 0.7% of PIP/TAZ patients. No death was considered drug-related.

**Table 3.** Summary of Adverse Events During Intravenous Therapy and the 14-Day Follow-up Period in the Safety Population

Patients With Adverse Events	IMI/REL (n = 266^a^)	PIP/TAZ (n = 269^a^)	Unadjusted Difference, % (95% CI)^b^
At least 1 AE	226 (85.0)	233 (86.6)	−1.7 (−7.7 to 4.3)
Drug-related^c^ AEs	31 (11.7)	26 (9.7)	2.0 (−3.3 to 7.4)
Serious AEs	71 (26.7)	86 (32.0)	−5.3 (−13.0 to 2.5)
Serious drug-related^c^ AEs	3 (1.1)	2 (0.7)	0.4 (−1.7 to 2.6)
Deaths	40 (15.0)	57 (21.2)	−6.2 (−12.7 to .4)
Drug-related^c^ deaths	0 (0.0)	0 (0.0)	0.0 (−1.4 to 1.4)
Discontinued drug due to AE	15 (5.6)	22 (8.2)	−2.5 (−7.1 to 1.8)
Discontinued drug due to drug-related^c^ AE	6 (2.3)^d^	4 (1.5)^e^	0.8 (−1.8 to 3.5)

Data are presented as the number (%) of patients who had at least 1 of the indicated type of AE, unless otherwise indicated.

Abbreviations: AE, adverse event; CI, confidence interval; IMI/REL, imipenem/cilastatin with relebactam; PIP/TAZ, piperacillin/tazobactam.

^a^Overall values indicate the total number of patients in the safety population of the particular treatment arm.

^b^Based on the Miettinen and Nurminen method [28].

^c^AE causality in relation to the study therapy was determined by the investigator.

^d^Specific drug-related AEs that led to study therapy discontinuation were as follows: liver function abnormalities (n = 2), rash (n = 2), and thrombocytopenia/decreased platelet count (n = 2).

^e^Specific drug-related AEs that led to study therapy discontinuation were as follows: liver function abnormalities (n = 1), hallucinations (n = 1), generalized tonic-clonic seizure (n = 1), and pyrexia (n = 1).

## DISCUSSION

This randomized, controlled, double-blind trial demonstrated noninferiority of IMI/REL to PIP/TAZ for the treatment of adult HABP/VABP in the primary endpoint of 28-day ACM and the key secondary endpoint of favorable clinical response at EFU. All other secondary endpoints were comparable between treatment arms. The study population consisted largely of patients at increased risk of adverse treatment outcomes and death, reflected in the high proportion of participants enrolled in the ICU, with APACHE II scores ≥15, with either augmented renal clearance or moderate/severe renal impairment, and of elderly patients. Approximately half were mechanically ventilated at initiation of study therapy. Baseline characteristics and causative pathogens were generally balanced between treatment arms; despite differences in baseline susceptibility profiles, clinical and microbiologic outcomes were similar between treatments. Causative pathogens, including key gram-negative bacteria generally seen in HABP/VABP, were similar to other recently completed clinical trials in nosocomial pneumonia and to surveillance studies [16, 18, 29–31]. Most patients experienced ≥1 treatment-emergent AE; a high AE rate was expected from this trial enrolling a severely ill patient population. In contrast, approximately 10% of patients per arm experienced drug-related AEs. IMI/REL was generally well tolerated, with few serious drug-related AEs, few therapy discontinuations due to drug-related AEs, and no drug-related deaths. The safety and tolerability profile of IMI/REL, including specific AEs, was comparable to that of PIP/TAZ. No new safety signals with IMI/REL were observed.

Treatment outcomes in these high-risk patients receiving IMI/REL were generally favorable and similar to previous studies, even though RESTORE-IMI 2 enrolled a more critically ill patient population than some other recent trials in this setting [5, 29]. Mortality patterns in our trial aligned with a recent meta-analysis showing average 28-day ACM to be lowest in nonventilated HABP, followed by VABP, and highest in ventilated HABP. Of note, in the predefined subgroup of mechanically ventilated patients, 28-day mortality was lower (the 95% CI for treatment difference excluded 0) with IMI/REL than PIP/TAZ in both the MITT and mMITT populations. Treatment differences were also apparent in the predefined subpopulation of patients with APACHE II score ≥15 (which was a randomization stratum), where 28-day mortality was lower and favorable clinical response at EFU was higher for IMI/REL, with 95% CIs also excluding 0. In an mMITT analysis of patients who had all baseline pathogens susceptible to both IMI/REL and PIP/TAZ, per-pathogen outcomes with Enterobacterales and *A. calcoaceticus-baumannii* complex were comparable between treatment arms. Patients with *P. aeruginosa* infections had comparable microbiologic eradication rates in both treatment arms at EOT (67% IMI/REL vs 72% PIP/TAZ), but lower clinical response and higher day 28 mortality rates in the IMI/REL arm. This may be attributable to differences between the treatment arms unrelated to the causative pathogen. Several of these patients developed serious/fatal AEs unrelated to their pneumonia, which subsequently led to unfavorable outcomes in mortality and/or clinical response. In addition, the sample size of this subpopulation was very small, and the IMI/REL arm had a smaller denominator (ie, 40% fewer patients with *P. aeruginosa* than the PIP/TAZ arm in that particular mMITT analysis), which may have contributed to the numeric differences in event rates.

Based on our results, IMI/REL is an effective new treatment option for HABP/VABP, including infections in mechanically ventilated, critically ill, and other high-risk patient populations. Carbapenems, such as IMI, remain a HABP/VABP treatment cornerstone [3, 4] due to their broad-spectrum efficacy (including ESBL activity), good tolerability, and extensive clinical experience with their use. However, increasing rates of carbapenem resistance worldwide require new treatment options [32, 33]. IMI/REL is active against many strains of multidrug-resistant gram-negative bacteria implicated in HABP/VABP and has demonstrated efficacy against IMI-nonsusceptible infections in a recent phase 3 trial [24]. The combination of IMI with REL overcomes key resistance mechanisms prevalent in gram-negative pathogens (eg, efflux, porin loss, and β-lactamase production/overexpression) [34–39], and has in vitro activity against most strains of KPC- and/or ESBL-producing Enterobacterales as well as multidrug- and/or carbapenem-resistant *P. aeruginosa*. REL does not inhibit metallo-β-lactamases (eg, NDM) and/or class D β-lactamases (eg, OXA-48); however, the combination of IMI/REL may have antibacterial activity against isolates encoding such enzymes due to inhibition of concomitantly expressed class A or C β-lactamases. Imipenem plus REL does not have added activity over IMI alone against the *A. calcoaceticus-baumannii* complex [11]. Of note, adding REL to IMI reduces the IMI minimum inhibitory concentration even in IMI-susceptible isolates [39], and the PK/PD properties of IMI/REL differ from those of IMI alone. Various preclinical analyses, lung penetration studies, population PK modeling, and probability of target attainment simulations all further support the 500 mg/500 mg/250 mg imipenem/cilastatin/relebactam dose (appropriately adjusted for renal function) used in our trial as being effective for HABP/VABP [12, 13, 19, 21–23]. This dosing regimen was also shown as effective in other clinical trials, including a phase 3 study that enrolled patients with serious infections (including HABP/VABP) due to carbapenem-nonsusceptible pathogens [19, 20, 24].

Our large, well-designed, randomized controlled trial meets current regulatory guidance for antibacterial development for this indication. All patients fulfilled standard definitions of HABP/VABP for enrollment [25]. The comparator drug is widely used for empiric and definitive treatment of HABP/VABP, is recommended in HABP/VABP clinical guidelines, and was administered at the standard dose [3, 4]. Randomization was successful, demonstrated by the fact that baseline characteristics and causative pathogens were comparable between arms, thus minimizing any potential bias. Notably, almost all randomized patients (~99%) received treatment and were included in the primary efficacy population, a much higher proportion than in many other recent HABP/VABP studies. Our study also had some limitations. First, not all enrolled patients were ventilated, which is the highest mortality risk subpopulation of HABP/VABP. Two other recent phase 3 studies of HABP/VABP enrolled only ventilated patients [29, 40]. Conversely, by enrolling both ventilated and nonventilated participants, our study population is representative of the wide range of patients with nosocomial pneumonia encountered in clinical practice. Second, the majority of study participants were enrolled outside the United States. However, this allowed for an evaluation of IMI/REL across different geographic regions and standards of care. Third, immunocompromised patients were excluded.

In conclusion, IMI/REL is noninferior to PIP/TAZ for treating HABP/VABP in adults. Both agents appeared well-tolerated based on the incidence of overall and serious drug-related AEs, and no new safety issues with IMI/REL were noted. IMI/REL is a new treatment option for HABP/VABP, including in high-risk patients.

## Supplementary Data

Supplementary materials are available at *Clinical Infectious Diseases* online. Consisting of data provided by the authors to benefit the reader, the posted materials are not copyedited and are the sole responsibility of the authors, so questions or comments should be addressed to the corresponding author.

ciaa803_suppl_Supplementary_Appendix

ciaa803_suppl_Supplementary_Protocol

## Notes

***Acknowledgments.*** The authors thank the patients and their families and caregivers for participating in this study, along with all investigators and site personnel. A full list of primary investigators for protocol MK-7655A-014 (by country) who enrolled patients is provided in Supplementary Table 11. Medical writing assistance was provided by Dominik Wolf, MSc, and editorial assistance was provided by Michele McColgan, BA, both of Merck Sharp & Dohme Corp, a subsidiary of Merck & Co, Inc, Kenilworth, New Jersey.

***Financial support.*** This work was supported by Merck Sharp & Dohme Corp, a subsidiary of Merck & Co, Inc, Kenilworth, New Jersey.

***Potential conflicts of interest.*** I. T., R. G. W., A. R., D. R. G., and A. D.-W. received institutional research funding from Merck & Co, Inc, for their participation in this clinical trial. H. W. B. and K. S. K. were paid advisory board members for Merck & Co, Inc. A. R. received personal fees from Merck Sharp & Dohme and bioMérieux. M. C. L., J. D., R. T., M. L. R., M. P., M. L. B., N. A. K., K. Y., J. R. B., A. P., and L. F. C. are employees of Merck Sharp & Dohme Corp, and may own stock and/or hold stock options in Merck & Co, Inc. All authors have submitted the ICMJE Form for Disclosure of Potential Conflicts of Interest. Conflicts that the editors consider relevant to the content of the manuscript have been disclosed.
