# Supplementary material for: A Randomized, Double-blind, Multicenter Trial Comparing Efficacy and Safety of Imipenem/Cilastatin/Relebactam Versus Piperacillin/Tazobactam in Adults With Hospital-acquired or Ventilator-associated Bacterial Pneumonia (RESTORE-IMI 2 Study)
Source: Clin Infect Dis. 2020 Aug 12;73(11):e4539–48. doi: 10.1093/cid/ciaa803 (PMC8662781; doi:10.1093/cid/ciaa803)
Supplement: ciaa803_suppl_Supplementary_Appendix [file ciaa803_suppl_supplementary_appendix.docx]

**Online supplementary appendix to:**

**Randomized, double-blind, multicenter trial (RESTORE-IMI 2) comparing efficacy and safety of imipenem/relebactam versus piperacillin/tazobactam in adults with hospital-acquired or ventilator-associated bacterial pneumonia**

Contents

[Supplementary Table 1: Reasons for exclusion from the various analysis populations. 3](#_Toc38369577)

[Supplementary Table 2: Use of prior (i.e., within 72 hours before first dose of study therapy) systemic antibacterial agents with gram-negative activity, as per drug names reported by the investigators. 4](#_Toc38369578)

[Supplementary Table 3: Treatment duration with randomized study drug. 6](#_Toc38369579)

[Supplementary Table 4: Use of concomitant, non-study systemic antibacterial agents with gram-negative activity, as per drug names reported by the investigators. 7](#_Toc38369580)

[Supplementary Table 5: Day 28 all-cause mortality in the modified intent-to-treat population, by randomization stratum. 8](#_Toc38369581)

[Supplementary Table 6: Overall favorable response at EFU in the modified intent-to-treat population, by randomization stratum. 9](#_Toc38369582)

[Supplementary Table 7: Proportion of patients with specific response categories for clinical and microbiologic response at the early follow-up visit. 10](#_Toc38369583)

[Supplementary Table 8. Primary and key secondary efficacy endpoints in patients who had all confirmed baseline LRT pathogens susceptible to both study drugs (mMITT population based on approved CLSI breakpoints). 11](#_Toc38369584)

[Supplementary Table 9: Per-pathogen favorable microbiologic response at EOT (assessed in mMITT patients who had all LRT pathogens susceptible to both IMI/REL and PIP/TAZ). 12](#_Toc38369585)

[Supplementary Table 10: Patients with specific drug-related adverse events of any incidence during IV therapy and the 14-day follow-up period. 13](#_Toc38369586)

[Supplementary Table 11: Number of patients randomized and treated by country and investigational site (with primary site investigator). 15](#_Toc38369587)

# Supplementary Table 1: Reasons for exclusion from the various analysis populations.

|  | **IMI/REL** | | | **PIP/TAZ** | | | **Total** | |
| --- | --- | --- | --- | --- | --- | --- | --- | --- |
|  | **n** | **(%)** | | **n** | **(%)** | | **n** | **(%)** |
| Patients in population | 268 | | | 269 | | | 537 | |
| **Safety** |  |  | |  |  | |  |  |
| Yes | 266 | (99.3) | | 269 | (100.0) | | 535 | (99.6) |
| No | 2 | (0.7) | | 0 |  | | 2 | (0.4) |
| Adverse Event | 1 | (0.4) | | 0 |  | | 1 | (0.2) |
| Physician Decision | 1 | (0.4) | | 0 |  | | 1 | (0.2) |
| **Modified Intent-To-Treat** |  |  | |  |  | |  |  |
| Yes | 264 | (98.5) | | 267 | (99.3) | | 531 | (98.9) |
| No | 4 | (1.5) | | 2 | (0.7) | | 6 | (1.1) |
| Not Treated | 2 | (0.7) | | 0 |  | | 2 | (0.4) |
| Gram-Positive Cocci Only | 2 | (0.7) | | 1 | (0.4) | | 3 | (0.6) |
| Missing Microscopy Result | 0 |  | | 1 | (0.4) | | 1 | (0.2) |
| **Microbiologic Modified Intent-To-Treat** |  |  | |  |  | |  |  |
| Yes | 215 | (80.2) | | 218 | (81.0) | | 433 | (80.6) |
| No | 53 | (19.8) | | 51 | (19.0) | | 104 | (19.4) |
| Not in Modified Intent-to-Treat | 4 | (1.5) | | 2 | (0.7) | | 6 | (1.1) |
| Baseline LRT Specimen Did Not Meet Culture and Identification Requirements for Inclusion | 49 | (18.3) | | 49 | (18.2) | | 98 | (18.2) |
| **Clinically-Evaluable** |  | |  |  | |  |  |  |
| Yes | 146 | | (54.5) | 144 | | (53.5) | 290 | (54.0) |
| No | 122 | | (45.5) | 125 | | (46.5) | 247 | (46.0) |
| Not in Modified Intent-to-Treat | 4 | | (1.5) | 2 | | (0.7) | 6 | (1.1) |
| Clinical Diagnostic Definition Not Met | 9 | | (3.4) | 4 | | (1.5) | 13 | (2.4) |
| Prior Antibacterial Violation | 34 | | (12.7) | 33 | | (12.3) | 67 | (12.5) |
| Inadequate Baseline LRT Specimen | 6 | | (2.2) | 5 | | (1.9) | 11 | (2.0) |
| Protocol Specified Minimum Duration of Study Therapy Not Received | 30 | | (11.2) | 45 | | (16.7) | 75 | (14.0) |
| Concomitant Antibacterial Violation | 1 | | (0.4) | 4 | | (1.5) | 5 | (0.9) |
| Confounding Antibacterial Post-Treatment | 32 | | (11.9) | 25 | | (9.3) | 57 | (10.6) |
| Confounding Medical Condition or Procedure | 6 | | (2.2) | 7 | | (2.6) | 13 | (2.4) |

IMI/REL, imipenem/cilastatin with relebactam. PIP/TAZ, piperacillin/tazobactam.

# Supplementary Table 2: Use of prior (i.e., within 72 hours before first dose of study therapy) systemic antibacterial agents with gram-negative activity, as per drug names reported by the investigators.

|  | **IMI/REL** | | **PIP/TAZ** | | **Total** | |
| --- | --- | --- | --- | --- | --- | --- |
|  | **n** | **(%)** | **n** | **(%)** | **n** | **(%)** |
| **Patients in population** | **264** |  | **267** |  | **531** |  |
| with one or more medications | 109 | (41.3) | 131 | (49.1) | 240 | (45.2) |
| with no medications | 155 | (58.7) | 136 | (50.9) | 291 | (54.8) |
| Amikacin | 2 | (0.8) | 4 | (1.5) | 6 | (1.1) |
| Amoxicillin | 3 | (1.1) | 2 | (0.7) | 5 | (0.9) |
| Amoxicillin trihydrate/clavulanate potassium | 2 | (0.8) | 3 | (1.1) | 5 | (0.9) |
| Amoxicillin/clavulanate potassium | 6 | (2.3) | 10 | (3.7) | 16 | (3.0) |
| Ampicillin | 0 | (0.0) | 1 | (0.4) | 1 | (0.2) |
| Ampicillin sodium/sulbactam sodium | 6 | (2.3) | 6 | (2.2) | 12 | (2.3) |
| Aztreonam | 0 | (0.0) | 1 | (0.4) | 1 | (0.2) |
| Cefalotin sodium | 3 | (1.1) | 0 | (0.0) | 3 | (0.6) |
| Cefazolin | 5 | (1.9) | 7 | (2.6) | 12 | (2.3) |
| Cefepime | 10 | (3.8) | 8 | (3.0) | 18 | (3.4) |
| Cefepime hydrochloride | 2 | (0.8) | 1 | (0.4) | 3 | (0.6) |
| Cefepime hydrochloride/glucose | 0 | (0.0) | 1 | (0.4) | 1 | (0.2) |
| Cefoperazone sodium/sulbactam sodium | 0 | (0.0) | 5 | (1.9) | 5 | (0.9) |
| Cefoperazone/sulbactam | 0 | (0.0) | 1 | (0.4) | 1 | (0.2) |
| Cefotaxime sodium | 3 | (1.1) | 0 | (0.0) | 3 | (0.6) |
| Cefoxitin | 1 | (0.4) | 0 | (0.0) | 1 | (0.2) |
| Cefozopran hydrochloride | 1 | (0.4) | 0 | (0.0) | 1 | (0.2) |
| Cefpodoxime proxetil | 1 | (0.4) | 1 | (0.4) | 2 | (0.4) |
| Ceftazidime | 3 | (1.1) | 0 | (0.0) | 3 | (0.6) |
| Ceftolozane sulfate/tazobactam sodium | 1 | (0.4) | 0 | (0.0) | 1 | (0.2) |
| Ceftriaxone | 21 | (8.0) | 28 | (10.5) | 49 | (9.2) |
| Ceftriaxone sodium | 5 | (1.9) | 3 | (1.1) | 8 | (1.5) |
| Cefuroxime | 3 | (1.1) | 6 | (2.2) | 9 | (1.7) |
| Cefuroxime sodium | 1 | (0.4) | 0 | (0.0) | 1 | (0.2) |
| Ciprofloxacin | 2 | (0.8) | 5 | (1.9) | 7 | (1.3) |
| Colistimethate sodium | 0 | (0.0) | 1 | (0.4) | 1 | (0.2) |
| Colistin | 1 | (0.4) | 1 | (0.4) | 2 | (0.4) |
| Ertapenem sodium | 4 | (1.5) | 2 | (0.7) | 6 | (1.1) |
| Gentamicin | 1 | (0.4) | 1 | (0.4) | 2 | (0.4) |
| Imipenem | 1 | (0.4) | 0 | (0.0) | 1 | (0.2) |
| Imipenem/cilastatin sodium | 1 | (0.4) | 4 | (1.5) | 5 | (0.9) |
| Levofloxacin | 8 | (3.0) | 13 | (4.9) | 21 | (4.0) |
| Meropenem | 12 | (4.5) | 8 | (3.0) | 20 | (3.8) |
| Meropenem trihydrate | 1 | (0.4) | 1 | (0.4) | 2 | (0.4) |
| Moxifloxacin hydrochloride | 0 | (0.0) | 1 | (0.4) | 1 | (0.2) |
| Piperacillin | 2 | (0.8) | 1 | (0.4) | 3 | (0.6) |
| Piperacillin sodium | 0 | (0.0) | 1 | (0.4) | 1 | (0.2) |
| Piperacillin sodium/tazobactam sodium | 24 | (9.1) | 40 | (15.0) | 64 | (12.1) |
| Polymyxin | 0 | (0.0) | 1 | (0.4) | 1 | (0.2) |
| Sulfamethoxazole/trimethoprim | 1 | (0.4) | 0 | (0.0) | 1 | (0.2) |
| Sultamicillin | 1 | (0.4) | 3 | (1.1) | 4 | (0.8) |
| Tigecycline | 1 | (0.4) | 0 | (0.0) | 1 | (0.2) |
| Tobramycin | 1 | (0.4) | 0 | (0.0) | 1 | (0.2) |
| Tobramycin sulfate | 0 | (0.0) | 1 | (0.4) | 1 | (0.2) |

Every patient is counted a single time for each applicable specific prior medication. A patient with multiple prior medications within a medication category is counted a single time for that category.

IMI/REL, imipenem/cilastatin with relebactam. PIP/TAZ, piperacillin/tazobactam.

# Supplementary Table 3: Treatment duration with randomized study drug.

| **Treatment group** | **≤2 days** | **>2 to ≤4 days** | **>4 to ≤6 days** | **>6 to ≤8 days** | **>8 to ≤10 days** | **>10 to ≤12 days** | **>12 to ≤14 days** | **>14**  **days** | **Total patients** | **Range** | **Mean** | **Median** |
| --- | --- | --- | --- | --- | --- | --- | --- | --- | --- | --- | --- | --- |
| **IMI/REL**  **PIP/TAZ** | 13  16 | 10  22 | 9  18 | 118  104 | 32  31 | 14  9 | 70  68 | 0  1 | 266  269 | 0 to 14 days  0 to 14 days | 8.7 days  8.3 days | 6.8 days  6.8 days |

Treatment duration defined as the number of days between the first and last dose of IV therapy: (end of last dose date/time-start of first dose date/time) / 24 hours.

IMI/REL, imipenem/cilastatin with relebactam. PIP/TAZ, piperacillin/tazobactam.

# Supplementary Table 4: Use of concomitant, non-study systemic antibacterial agents with gram-negative activity, as per drug names reported by the investigators.

|  | **IMI/REL** | | **PIP/TAZ** | | **Total** | |
| --- | --- | --- | --- | --- | --- | --- |
|  | **n** | **(%)** | **n** | **(%)** | **n** | **(%)** |
| **Patients in population** | **264** |  | **267** |  | **531** |  |
| with one or more medications | 21 | (8.0) | 28 | (10.5) | 49 | (9.2) |
| with no medications | 243 | (92.0) | 239 | (89.5) | 482 | (90.8) |
| Amikacin | 0 | (0.0) | 1 | (0.4) | 1 | (0.2) |
| Amoxicillin | 0 | (0.0) | 1 | (0.4) | 1 | (0.2) |
| Amoxicillin/clavulanate potassium | 2 | (0.8) | 2 | (0.7) | 4 | (0.8) |
| Ampicillin sodium/sulbactam sodium | 2 | (0.8) | 0 | (0.0) | 2 | (0.4) |
| Cefazolin | 3 | (1.1) | 2 | (0.7) | 5 | (0.9) |
| Cefazolin sodium | 0 | (0.0) | 1 | (0.4) | 1 | (0.2) |
| Cefepime | 1 | (0.4) | 1 | (0.4) | 2 | (0.4) |
| Cefoperazone sodium/sulbactam sodium | 0 | (0.0) | 1 | (0.4) | 1 | (0.2) |
| Cefoperazone/sulbactam | 1 | (0.4) | 0 | (0.0) | 1 | (0.2) |
| Cefotaxime sodium | 1 | (0.4) | 0 | (0.0) | 1 | (0.2) |
| Ceftriaxone | 1 | (0.4) | 1 | (0.4) | 2 | (0.4) |
| Ciprofloxacin | 1 | (0.4) | 2 | (0.7) | 3 | (0.6) |
| Colistimethate sodium | 0 | (0.0) | 1 | (0.4) | 1 | (0.2) |
| Colistin | 1 | (0.4) | 2 | (0.7) | 3 | (0.6) |
| Doxycycline | 0 | (0.0) | 1 | (0.4) | 1 | (0.2) |
| Gentamicin | 0 | (0.0) | 1 | (0.4) | 1 | (0.2) |
| Imipenem/cilastatin sodium | 0 | (0.0) | 2 | (0.7) | 2 | (0.4) |
| Levofloxacin | 0 | (0.0) | 4 | (1.5) | 4 | (0.8) |
| Meropenem | 4 | (1.5) | 8 | (3.0) | 12 | (2.3) |
| Ofloxacin | 1 | (0.4) | 0 | (0.0) | 1 | (0.2) |
| Piperacillin | 1 | (0.4) | 0 | (0.0) | 1 | (0.2) |
| Piperacillin sodium/tazobactam sodium | 3 | (1.1) | 5 | (1.9) | 8 | (1.5) |
| Polymyxin | 0 | (0.0) | 1 | (0.4) | 1 | (0.2) |
| Sulfamethoxazole/trimethoprim | 0 | (0.0) | 1 | (0.4) | 1 | (0.2) |
| Sultamicillin | 0 | (0.0) | 1 | (0.4) | 1 | (0.2) |
| Tobramycin | 1 | (0.4) | 1 | (0.4) | 2 | (0.4) |
| Tobramycin sulfate | 0 | (0.0) | 1 | (0.4) | 1 | (0.2) |

Every patient is counted a single time for each applicable specific prior medication. A patient with multiple prior medications within a medication category is counted a single time for that category.

IMI/REL, imipenem/cilastatin with relebactam. PIP/TAZ, piperacillin/tazobactam.

# Supplementary Table 5: Day 28 all-cause mortality in the modified intent-to-treat population, by randomization stratum.

|  | **IMI/REL** | | **PIP/TAZ** | | **Unadjusted Difference** | **Adjusted Difference** | | ***p*-value** |
| --- | --- | --- | --- | --- | --- | --- | --- | --- |
|  | **n/m** | **(%)** | **n/m** | **(%)** | **%** | **%** | **(CI)^a^** |  |
| **Overall modified intent-to-treat population** | 42/264 | (15.9) | 57/267 | (21.3) | -5.4 | -5.3 | (-11.9, 1.2)b | *p*b <0.001 |
|  |  |  |  |  |  |  | (-12.9, 2.1)c | *p*c =0.111 |
| Non-Ventilated HABP with Baseline APACHE II <15 | 10/102 | (9.8) | 6/102 | (5.9) | 3.9 | - | - | - |
| Non-Ventilated HABP with Baseline APACHE II ≥15 | 7/45 | (15.6) | 12/43 | (27.9) | -12.4 | - | - | - |
| Ventilated HABP/VABP with Baseline APACHE II <15 | 10/41 | (24.4) | 7/41 | (17.1) | 7.3 | - | - | - |
| Ventilated HABP/VABP with Baseline APACHE II ≥15 | 15/76 | (19.7) | 32/81 | (39.5) | -19.8 | - | - | - |
| n/m = number of patients with favorable clinical response / number of modified intent-to-treat patients who should have clinical response assessed at the corresponding visit. EFU, early follow-up. CI, confidence interval. IMI/REL, imipenem/cilastatin with relebactam. PIP/TAZ, piperacillin/tazobactam.  ^a^Adjusted differences and the corresponding confidence intervals are based on Miettinen & Nurminen method stratified by randomization -stratum.  ^b^The 95% CI and the *p*-value are for the non-inferiority hypothesis test where the non-inferiority margin is -12.5% and alpha level (one sided) is 0.025.  ^c^The 97.5% CI and the *p*-value are for the superiority hypothesis test where the alpha level (two sided) is 0.025.  Note: Type of HAP/VAP was one of the stratification factors in this study: at the time of randomization/stratification, participants were stratified by either (a) non-ventilated HAP or (b) ventilated HAP/VAP. This information was captured via the interactive randomization system at the time of randomization/stratification as ‘Ventilation status at randomization’, and the data shown in this table reflect those numbers. The diagnosis was also separately captured in the clinical database after participants had been randomized/stratified – this information is reflected in the demographics table (Table 1 in main manuscript) as ‘Primary diagnosis’.  Any discrepancy in the numbers of MITT patients between these two categories can be attributed to one of two factors. One, there could have been a clerical error at the time of entering the HAP/VAP diagnosis into the interactive system during randomization/stratification. Any such errors occurring at the time of randomization/stratification in the interactive system could not be changed retroactively. Two, there could have been disease improvement or progression between screening and randomization/stratification. There was a slight time lag between when the primary diagnosis was first assessed during the screening period and when the participant was actually randomized/stratified into the study. During that lag between the two steps, patients may have experienced disease progression and had to be ventilated or conversely improved sufficiently to be taken off mechanical ventilation. | | | | | | | | |

# Supplementary Table 6: Overall favorable response at EFU in the modified intent-to-treat population, by randomization stratum.

|  | **IMI/REL** | | **PIP/TAZ** | | **Unadjusted Difference** | **Adjusted Difference** | | ***p*-value** |
| --- | --- | --- | --- | --- | --- | --- | --- | --- |
|  | **n/m** | **(%)** | **n/m** | **(%)** | **%** | **%** | **(CI)^a^** |  |
| **Overall modified intent-to-treat population** | 161/264 | (61.0) | 149/267 | (55.8) | 5.2 | 5.0 | (-3.2, 13.2)^b^ | *p*^b^ <0.001 |
|  |  |  |  |  |  |  | (-4.4, 14.4)_b_ | *p*^c^ =0.235 |
| Non-Ventilated HABP with Baseline APACHE II <15 | 70/102 | (68.6) | 74/102 | (72.5) | -3.9 | - | - | - |
| Non-Ventilated HABP with Baseline APACHE II ≥15 | 27/45 | (60.0) | 20/43 | (46.5) | 13.5 | - | - | - |
| Ventilated HABP/VABP with Baseline APACHE II <15 | 22/41 | (53.7) | 24/41 | (58.5) | -4.9 | - | - | - |
| Ventilated HABP/VABP with Baseline APACHE II ≥15 | 42/76 | (55.3) | 31/81 | (38.3) | 17.0 | - | - | - |
| n/m = number of patients with favorable clinical response / number of modified intent-to-treat patients who should have clinical response assessed at the corresponding visit. EFU, early follow-up. CI, confidence interval. IMI/REL, imipenem/cilastatin with relebactam. PIP/TAZ, piperacillin/tazobactam.  ^a^Adjusted differences and the corresponding confidence intervals are based on Miettinen & Nurminen method stratified by randomization -stratum.  ^b^The 95% CI and the *p*-value are for the non-inferiority hypothesis test where the non-inferiority margin is -12.5% and alpha level (one sided) is 0.025.  ^c^The 97.5% CI and the *p*-value are for the superiority hypothesis test where the alpha level (two sided) is 0.025.  Note: Type of HAP/VAP was one of the stratification factors in this study: at the time of randomization/stratification, participants were stratified by either (a) non-ventilated HAP or (b) ventilated HAP/VAP. This information was captured via the interactive randomization system at the time of randomization/stratification as ‘Ventilation status at randomization’, and the data shown in this table reflect those numbers. The diagnosis was also separately captured in the clinical database after participants had been randomized/stratified – this information is reflected in the demographics table (Table 1 in main manuscript) as ‘Primary diagnosis’.  Any discrepancy in the numbers of MITT patients between these two categories can be attributed to one of two factors. One, there could have been a clerical error at the time of entering the HAP/VAP diagnosis into the interactive system during randomization/stratification. Any such errors occurring at the time of randomization/stratification in the interactive system could not be changed retroactively. Two, there could have been disease improvement or progression between screening and randomization/stratification. There was a slight time lag between when the primary diagnosis was first assessed during the screening period and when the participant was actually randomized/stratified into the study. During that lag between the two steps, patients may have experienced disease progression and had to be ventilated or conversely improved sufficiently to be taken off mechanical ventilation. | | | | | | | | |

# Supplementary Table 7: Proportion of patients with specific response categories for clinical and microbiologic response at the early follow-up visit.

|  | **IMI/REL** | | **PIP/TAZ** | |
| --- | --- | --- | --- | --- |
|  | **n** | **(%)** | **n** | **(%)** |
| **Clinical response in the modified intent-to-treat population** | | | | |
| Number of patients in modified intent-to-treat population | 264 | | 267 | |
| Sustained cure | 124 | (47.0%) | 105 | (39.3%) |
| Cure | 37 | (14.0%) | 44 | (16.5%) |
| Indeterminate | 42 | (15.9%) | 62 | (23.2%) |
| Complication related to underlying medical condition | 6 | (2.3%) | 9 | (3.4%) |
| Death occurred during the study period and the index infection was clearly noncontributory | 12 | (4.5%) | 26 | (9.7%) |
| Extenuating circumstances preclude classification as improved, persistence, or progression | 18 | (6.8%) | 21 | (7.9%) |
| Patient was withdrawn for any reason before sufficient data had been obtained to permit clinical response evaluation | 6 | (2.3%) | 6 | (2.2%) |
| Relapse | 12 | (4.5%) | 3 | (1.1%) |
| Failure | 26 | (9.8%) | 29 | (10.9%) |
| Missing | 23 | (8.7%) | 24 | (9.0%) |
| **Microbiologic response in the microbiologic modified intent-to-treat population** | | | | |
| Number of patients in microbiologic modified intent-to-treat population | 215 | | 218 | |
| Eradication | 73 | (34.0%) | 67 | (30.7%) |
| Presumed Eradication | 73 | (34.0%) | 68 | (31.2%) |
| Persistence | 5 | (2.3%) | 9 | (4.1%) |
| Recurrence | 7 | (3.3%) | 2 | (0.9%) |
| Indeterminate | 57 | (26.5%) | 72 | (33.0%) |

IMI/REL, imipenem/cilastatin with relebactam. PIP/TAZ, piperacillin/tazobactam.

# Supplementary Table 8. Primary and key secondary efficacy endpoints in patients who had all confirmed baseline LRT pathogens susceptible to both study drugs (mMITT population based on approved CLSI breakpoints).

|  | **IMI/REL**  **n/N (%)** | **PIP/TAZ**  **n/N (%)** | **Unadjusted difference** | **Adjusted difference (95% CI)** |
| --- | --- | --- | --- | --- |
| Patients in population | 89 | 96 |  |  |
| **Primary endpoint** |  |  |  |  |
| Day 28 all-cause mortality | 14/89 (15.7%) | 18/96 (18.8%) | -3.0% | -4.8 (-16.1, 6.1) |
| **Key secondary endpoint** |  |  |  |  |
| Favorable clinical response at EFU | 57/89 (64.0%) | 64/96 (66.7%) | -2.6% | 0.9 (-12.6, 14.0) |

Adjusted differences and corresponding confidence intervals stratified by pneumonia type (nonventilated HABP vs. ventilated HABP/VABP) and by baseline APACHE II score (<15 vs. ≥15) using the Miettinen & Nurminen method.

n/m = number of patients with specific endpoint / number of modified intent-to-treat patients with all baseline pathogen from LRT culture susceptible to IMI/REL and PIP/TAZ based on CLSI-approved MIC breakpoints.

EFU, early follow-up. CI, confidence interval. CLSI, Clinical Laboratory Standards Institute. IMI/REL, imipenem/cilastatin with relebactam. LRT, lower respiratory tract. MIC, minimum inhibitory concentration. mMITT, microbiologic modified intent-to-treat population. PIP/TAZ, piperacillin/tazobactam.

# Supplementary Table 9: Per-pathogen favorable microbiologic response at EOT (assessed in mMITT patients who had all LRT pathogens susceptible to both IMI/REL and PIP/TAZ).

|  | **IMI/REL**  **n/N (%)** | **PIP/TAZ**  **n/N (%)** | **Adjusted difference^a^ (95% CI)** |
| --- | --- | --- | --- |
| Enterobacterales^b^ | 56/68 (82.4%) | 49/66 (74.2%) | 10.9% (-2.7, 25.1) |
| *Pseudomonas aeruginosa*^c^ | 10/15 (66.7%) | 18/25 (72.0%) | -5.3% (-35.3, 22.7) |
| *Acinetobacter calcoaceticus-baumannii* complex^c^ | 1/1 (100.0%) | 3/4 (75.0%) | 25.0% |
| CI, confidence interval. EFU, early follow-up visit. IMI/REL, imipenem/cilastatin with relebactam. LRT, lower respiratory tract. mMITT, microbiologic modified intent-to-treat population. N, total number of patients in the particular analysis population and treatment arm. n, number of patients who died or had unknown survival status (for mortality endpoints) or number of patients with favorable response (for response endpoints). PIP/TAZ, piperacillin/tazobactam.  ^a^Differences and confidence are based on the Miettinen & Nurminen method.  ^b^Assessed in patients who only had Enterobacterales (of any species) confirmed as baseline LRT isolates.  ^c^Assessed in patients who had ≥1 confirmed baseline LRT isolate of this pathogen, regardless of whether other LRT pathogens were also isolated at baseline. | | | |

# Supplementary Table 10: Patients with specific drug-related adverse events of any incidence during IV therapy and the 14-day follow-up period.

|  | **IMI/REL** | | | **PIP/TAZ** | | | **Total** | | |
| --- | --- | --- | --- | --- | --- | --- | --- | --- | --- |
|  | **n** | **(%)** | | **n** | **(%)** | | **n** | **(%)** | |
| Patients in population | 266 |  | | 269 |  | | 535 |  | |
| with one or more drug-related adverse events | 31 | (11.7) | | 26 | (9.7) | | 57 | (10.7) | |
| with no drug-related adverse events | 235 | (88.3) | | 243 | (90.3) | | 478 | (89.3) | |
| **Blood and lymphatic system disorders** | **4** | **(1.5)** | | **1** | **(0.4)** | | **5** | **(0.9)** | |
| Leukopenia | 0 | (0.0) | | 1 | (0.4) | | 1 | (0.2) | |
| Thrombocytopenia | 4 | (1.5) | | 0 | (0.0) | | 4 | (0.7) | |
| **Gastrointestinal disorders** | **9** | **(3.4)** | | **9** | **(3.3)** | | **18** | **(3.4)** | |
| Diarrhea | 6 | (2.3) | | 6 | (2.2) | | 12 | (2.2) | |
| Dysbiosis | 0 | (0.0) | | 1 | (0.4) | | 1 | (0.2) | |
| Epigastric discomfort | 0 | (0.0) | | 1 | (0.4) | | 1 | (0.2) | |
| Feces soft | 1 | (0.4) | | 0 | (0.0) | | 1 | (0.2) | |
| Nausea | 1 | (0.4) | | 1 | (0.4) | | 2 | (0.4) | |
| Vomiting | 2 | (0.8) | | 1 | (0.4) | | 3 | (0.6) | |
| **General disorders and administration site** | **3** | **(1.1)** | | **4** | **(1.5)** | | **7** | **(1.3)** | |
| **conditions** |  |  | |  |  | |  |  | |
| Infusion site pain | 0 | (0.0) | | 1 | (0.4) | | 1 | (0.2) | |
| Injection site pain | 0 | (0.0) | | 1 | (0.4) | | 1 | (0.2) | |
| Injection site phlebitis | 1 | (0.4) | | 0 | (0.0) | | 1 | (0.2) | |
| Injection site reaction | 0 | (0.0) | | 1 | (0.4) | | 1 | (0.2) | |
| Injection site swelling | 2 | (0.8) | | 1 | (0.4) | | 3 | (0.6) | |
| Pyrexia | 0 | (0.0) | | 1 | (0.4) | | 1 | (0.2) | |
| **Hepatobiliary disorders** | **1** | **(0.4)** | | **0** | **(0.0)** | | **1** | **(0.2)** | |
| Hepatic function abnormal | 1 | (0.4) | | 0 | (0.0) | | 1 | (0.2) | |
| **Infections and infestations** | **4** | **(1.5)** | | **2** | **(0.7)** | | **6** | **(1.1)** | |
| Antibiotic associated colitis | 1 | (0.4) | | 0 | (0.0) | | 1 | (0.2) | |
| Clostridium difficile colitis | 1 | (0.4) | | 0 | (0.0) | | 1 | (0.2) | |
| Fungal disease carrier | 0 | (0.0) | | 1 | (0.4) | | 1 | (0.2) | |
| Fungal skin infection | 0 | (0.0) | | 1 | (0.4) | | 1 | (0.2) | |
| Gastroenteritis | 1 | (0.4) | | 0 | (0.0) | | 1 | (0.2) | |
| Vulvovaginal candidiasis | 1 | (0.4) | | 0 | (0.0) | | 1 | (0.2) | |
| **Injury, poisoning and procedural** | **0** | | **(0.0)** | **1** | | **(0.4)** | **1** | | **(0.2)** |
| **complications** |  | |  |  | |  |  | |  |
| Infusion related reaction | 0 | | (0.0) | 1 | | (0.4) | 1 | | (0.2) |
| **Investigations** | **10** | | **(3.8)** | **4** | | **(1.5)** | **14** | | **(2.6)** |
| Alanine aminotransferase increased | 6 | | (2.3) | 3 | | (1.1) | 9 | | (1.7) |
| Aspartate aminotransferase increased | 6 | | (2.3) | 0 | | (0.0) | 6 | | (1.1) |
| Blood alkaline phosphatase increased | 4 | | (1.5) | 1 | | (0.4) | 5 | | (0.9) |
| Blood potassium decreased | 1 | | (0.4) | 0 | | (0.0) | 1 | | (0.2) |
| Blood sodium increased | 1 | | (0.4) | 0 | | (0.0) | 1 | | (0.2) |
| Gamma-glutamyltransferase increased | 1 | | (0.4) | 1 | | (0.4) | 2 | | (0.4) |
| Platelet count decreased | 1 | | (0.4) | 0 | | (0.0) | 1 | | (0.2) |
| **Metabolism and nutrition disorders** | **2** | | **(0.8)** | **1** | | **(0.4)** | **3** | | **(0.6)** |
| Hypokalemia | 2 | | (0.8) | 1 | | (0.4) | 3 | | (0.6) |
| Hypomagnesaemia | 1 | | (0.4) | 0 | | (0.0) | 1 | | (0.2) |
| Hyponatremia | 1 | | (0.4) | 0 | | (0.0) | 1 | | (0.2) |
| **Nervous system disorders** | **0** | | **(0.0)** | **2** | | **(0.7)** | **2** | | **(0.4)** |
| Dizziness | 0 | | (0.0) | 1 | | (0.4) | 1 | | (0.2) |
| Dysgeusia | 0 | | (0.0) | 1 | | (0.4) | 1 | | (0.2) |
| Generalized tonic-clonic seizure | 0 | | (0.0) | 1 | | (0.4) | 1 | | (0.2) |
| **Psychiatric disorders** | **0** | | **(0.0)** | **1** | | **(0.4)** | **1** | | **(0.2)** |
| Hallucination, visual | 0 | | (0.0) | 1 | | (0.4) | 1 | | (0.2) |
| **Renal and urinary disorders** | **0** | | **(0.0)** | **1** | | **(0.4)** | **1** | | **(0.2)** |
| Renal impairment | 0 | | (0.0) | 1 | | (0.4) | 1 | | (0.2) |
| **Skin and subcutaneous tissue disorders** | **6** | | **(2.3)** | **2** | | **(0.7)** | **8** | | **(1.5)** |
| Petechiae | 1 | | (0.4) | 0 | | (0.0) | 1 | | (0.2) |
| Pruritus generalized | 0 | | (0.0) | 1 | | (0.4) | 1 | | (0.2) |
| Rash | 3 | | (1.1) | 1 | | (0.4) | 4 | | (0.7) |
| Rash generalized | 1 | | (0.4) | 0 | | (0.0) | 1 | | (0.2) |
| **Skin and subcutaneous tissue disorders** | **6** | | **(2.3)** | **2** | | **(0.7)** | **8** | | **(1.5)** |
| Urticaria | 1 | | (0.4) | 0 | | (0.0) | 1 | | (0.2) |

Every patient is counted a single time for each applicable row and column.

IMI/REL, imipenem/cilastatin with relebactam. PIP/TAZ, piperacillin/tazobactam.

# Supplementary Table 11: Number of patients randomized and treated by country and investigational site (with primary site investigator).

| **Primary Investigator** | **Site Name** | **Number of Patients Randomized** |
| --- | --- | --- |
| **Argentina** | | **5** |
| Lorena Ravera | Hospital Dr Guillermo Rawson Cordoba | 1 |
| Analia Mykietiuk | Instituto Medico Platense La Plata | 4 |
| **Brazil** | | **44** |
| Antonio Tarcisio de Faria Freire | Santa Casa de Misericordia de Belo Horizonte Belo Horizonte | 18 |
| Maria Patelli Juliani Souza Lima | Hospital e Maternidade Celso Pierro  Campinas | 1 |
| Alvaro Rea-Neto | Hospital do Trabalhador Curitiba | 3 |
| Suzana Margareth Ajeje Lobo | Hospital de Base - São José do Rio Preto São José do Rio Preto | 12 |
| Alvaro Rea-Neto | Hospital Universitario Cajuru Curitiba | 10 |
| **Bulgaria** | | **21** |
| Vasil Tsenov | MHAT City Clinic Sv. Georgi EOOD Montana | 6 |
| Nikolay Ignatov | UMHAT Deva Maria Burgas | 2 |
| Rumen Tiholov | MHAT Sv. Ivan Rilski EOOD Kozloduy | 5 |
| Blanche Angelova-Kotova | MBAL Medica Ruse EOOD Bulgaria | 1 |
| Hristo Metev | SHATPPD Dr. Dimitar Gramatikov - Ruse EOOD Ruse | 3 |
| Vanushka Petrova | MHAT - Sliven at MMA Sofia Sliven | 1 |
| Nikolay Uvaliev | SHAT of Cardiosurgery City Clinic Burgas OOD Burgas | 3 |
| **Canada** | | **1** |
| Martin Girard | Centre Hospitalier de l Universite de Montreal - CHUM Soins Intensifs | 1 |
| **Colombia** | | **21** |
| Wilder Castano Osorio | Fundacion Cardiomet CEQUIN  Armenia | 13 |
| Leonardo Brochado | Clinica de la Costa Ltda. Atlantico | 8 |
| **Croatia** | | **1** |
| Iveta Mercep | Klinicki Bolnicki Centar Zagreb Zagreb | 1 |
| **Czech Republic** |  | **2** |
| Jiri Vyhnal | Nemocnice Kyjov  Kyjov | 2 |
| **Estonia** | | **12** |
| Mait Altmets | North Estonia Medical Centre Tallinn | 8 |
| Veronika Reinhard | Tartu University Hospital Tartu | 4 |
| **France** | | **34** |
| Antoine Roquilly | CHU de Nantes - Hotel Dieu Nantes | 18 |
| Herve Dupont | CHU Amiens Picardie Site Sud Amiens Amiens | 4 |
| Saad Nseir | C.H.R.U. de Lille, Hopital Roger Salengro Lille | 2 |
| Gaetan Plantefeve | Centre Hospitalier Victor Dupouy Argenteuil | 8 |
| Nadia Anguel | CHU - Hopital de Bicetre Le Kremlin Bicetre | 2 |
| **Georgia** | | **18** |
| Vakhtang Kaloiani | New Hospitals Ltd Tbilisi | 2 |
| Nino Babunashvili | Evex Medical Corporation JSC Kutaisi | 7 |
| Irakli Panchulidze | Kutaisi Central Hospital LTD Kutaisi | 2 |
| Mamuka Nemsitsveridze | West Georgian National Centre of Interventional Medicine Kutaisi | 4 |
| Tamar Gogichaishvili | Tbilisi Central Hospital Tbilisi | 3 |
| **Guatemala** | | **5** |
| Luis Demetrio Gonzalez Patzan | Hospital Centro Medico Militar Santa Rosita | 5 |
| **Italy** | | **2** |
| Matteo Bassetti | Azienda Ospedaliera Universitaria Santa Maria della Misericordia  Udine | 1 |
| Antonio Corcione | Azienda Ospedaliera R. N. V. Monaldi Napoli | 1 |
| **Japan** | | **43** |
| Takafumi Miyachi | Yanai Medical Center Yamaguchi | 1 |
| Shuichi Yano | National Hospital Organization Matsue Medical Center Shimane | 1 |
| Kenji Kono | Fukuoka Kinen Hospital Fukuoka | 2 |
| Tetsuro Hamasaki / Ikkou Higashimoto | Kagoshima City Hospital Kagoshima | 1 |
| Takeo Endo | National Hospital Organization Mito Medical Center Ibaraki | 2 |
| Hiroki Yamanoue | Shizuoka Tokushukai Hospital Shizuoka | 3 |
| Yuichi Fukuda | Sasebo City General Hospital Nagasaki | 1 |
| Takeshi Takahashi | National Hospital Organization Kumamoto Medical Center Kumamoto | 1 |
| Taiga Miyazaki | Nagasaki University Hospital Nagasaki | 1 |
| Hiroshi Hayakawa | National Hospital Organization Tenryu Hospital Shizuoka | 3 |
| Tsutomu Kobayashi | Sasebo Chuo Hospital Nagasaki | 1 |
| Reiki Kuroki / Takayasu Fukudome | National Hospital Organization Nagasaki Kawatana Medical Center Nagasaki | 2 |
| Toshiyuki Kita | National Hospital Organization Kanazawa Medical Center Ishikawa | 1 |
| Masafumi Masuda | Shizuoka City Shimizu Hospital Shizuoka | 1 |
| Masako Kadowaki | National Hospital Organization Fukuoka Hospital Fukuoka | 2 |
| Shigeki Hashimoto | Ikeda Municipal Hospital Osaka | 2 |
| Kiyotaka Ito | Kumamoto Rosai Hospital Kumamoto | 1 |
| Hidenori Ibata | National Hospital Organization Mie Chuo Medical Center Mie | 2 |
| Yasuyuki Tanabe | National Hospital Organization Minami-Okayama Medical Center Okayama | 3 |
| Hiroyasu Ishikura | Fukuoka University Hospital Fukuoka | 1 |
| Takahiro Ashikawa / Tadayuki Hashimoto | National Hospital Organization Minami Wakayama Medical Center Wakayama | 3 |
| Hiroshi Yamamoto | Tokyo Metropolitan Geriatric Hospital Tokyo | 1 |
| Hirokazu Tojima | Japan Labour Health and Safety Organization Tokyo Rosai Hospital Tokyo | 2 |
| Kazunori Tobino | Aso Iizuka Hospital Fukuoka | 1 |
| Kensuke Nakamura | Hitachi General Hospital Ibaraki | 3 |
| Hiroshi Takahashi | Saka General Hospital Miyagi | 1 |
| **Latvia** | | **3** |
| Dainis Krievins / Eva Strike | Pauls Stradins Clinical University Hospital Riga | 2 |
| Nikolajs Novikov | Daugavpils Regional Hospital Daugavpils | 1 |
| **Lithuania** | | **7** |
| Gintautas Kekstas | Vilniaus Universiteto Ligonine Santaros Klinikos Vilnius | 7 |
| **Mexico** | | **34** |
| Luis Alfredo Ponce de Leon G | Instituto Nacional de Ciencias Medicas y Nutricion Salvador Zubiran Ciudad de Mexico | 2 |
| Daniel Rodriguez Gonzalez | Hospital Civil de Guadalajara Dr. Juan I. Menchaca Guadalajara | 22 |
| Eduardo Rodriguez Noriega | Hospital Civil de Guadalajara Fray Antonio Alcalde Guadalajara | 8 |
| Martin Magana Aquino | Hospital Central Dr. Ignacio Morones Prieto San Luis Potosi | 2 |
| **Norway** | | **6** |
| Jan Erik Berdal | Akershus Universitetssykehus HF Loerenskog | 6 |
| **Philippines** | | **27** |
| Joven Roque Verzosa Gonong | Lung Center of the Philippines Quezon City | 1 |
| Ronnie Zamora Samoro | West Visayas State University Medical Center Iloilo City | 4 |
| Aileen David-Wang | University of the Philippines-Philippine General Hospital Manila | 13 |
| Arnold D. Germar | Veterans Memorial Medical Center Quezon City | 1 |
| Marie Grace Dawn Tindog Isidro | West Visayas State University Medical Center Iloilo City | 6 |
| Ronald Allan Payumo | Mary Johnston Hospital Manilla | 2 |
| **Portugal** | | **3** |
| Ana Margarida Araujo | Hospital Vila Franca Xira Vila Franca de Xira | 1 |
| Rosa Jorge | Centro Hospitalar do Baixo Vouga E.P.E. Aveiro | 1 |
| Anabela Salgueiro Marques / Jana Zelinova | Hospital de Cascais Dr. Jose de Almeida-HPP Alcabideche | 1 |
| **Romania** | | **30** |
| Dan Corneci | Spitalul Universitar de Urgenta Elias Bucuresti | 1 |
| Ovidiu Horea Bedreag | Spitalul Clinic Judetean de Urgenta Pius Branzeu Timisoara | 27 |
| Luminita Ionica Neagoe | Spitalul Clinic de Urgenta Bagdasar-Arseni Bucharest | 2 |
| **Russia** | | **35** |
| Vladimir Simanenkov | City Clinical Hospital #26 Saint Petersburg | 8 |
| Yury Uspenskiy | Saint Elizabeth the Martyr City Hospital SPb SHI Saint Petersburg | 3 |
| Mikhail Nikolaevich Zamyatin | National Medical and Surgical Center n.a. N.I.Pirogov Moscow | 5 |
| Tatiana Rudolfovna Kameneva | City Hospital #3 Zelenograd | 2 |
| Natalia Anatolevna Petrochenkova | Smolensk State Medical Academy Smolensk | 8 |
| Alina Agafina | City Hospital #40 Saint Petersburg | 5 |
| Julia Shapovalova | Railway Clinical Hospital Chelyabinsk | 2 |
| Yury Spesivtsev | Mariinskaya City Hospital Saint-Petersburg | 2 |
| **Serbia** | | **7** |
| Jovan Matijasevic | Institut za plucne bolesti Vojvodine Sremska Kamenica Sremska Kamenica | 4 |
| Ivan Golub Palibrk | Klinicki centar Srbije. Beograd | 3 |
| **South Korea** | | **7** |
| Jang Wook Sohn | Korea University Anam Hospital Seoul | 1 |
| Hong Bin Kim | Seoul National University Bundang Hospital Gyeonggi-do | 1 |
| Seok Chan Kim | The Catholic University of Korea. Seoul St. Mary s Hospital Seoul | 1 |
| Sang Haak Lee | The Catholic University of Korea Eunpyeong St Mary s Hospital Seoul | 4 |
| **Spain** | | **7** |
| Juan Jose Caston Osorio | Hospital Universitario Reina Sofia Cordoba | 7 |
| **Turkey** | | **13** |
| Iftihar Koksal | Karadeniz Teknik Universitesi Tip Fakultesi Farabi Hastanesi Trabzon | 4 |
| Oguz Karabay | Sakarya Universitesi Egitim ve Arastirma Hastanesi Sakarya | 3 |
| Gokhan Methan | Hacettepe University Medical Faculty Ankara | 3 |
| Yasar Bayindir | Inonu University Faculty of Medicine Malatya | 3 |
| **Ukraine** | | **128** |
| Sergii Dubrov | Kiyv City Municipal Hospital 17 Kiev | 8 |
| Ivan Titov | Ivano-Frankivsk Regional Clinical Hospital Ivano-Frankivsk | 60 |
| Oleksandr Pavlov | Institute of General and Emergency Surgery n.a Zaitsev NAMS of Ukraine Kharkiv | 35 |
| Viktor Blazhko | Kharkiv City Clinical Hospital #13 Kharkiv | 14 |
| Olena Klygunenko | ME Dnipropetrovsk Clinical Joinder of Emergency Care of DRC Dnipro | 7 |
| Artur Melnichenko | CI Central District Hospital of Zhytomyr District Council Stanyshivka | 4 |
| **USA** | | **21** |
| Nicholas Namias | University of Miami Miami, FL | 2 |
| Stefan N. Chock | Estudy Site San Diego, CA | 1 |
| Richard G. Wunderink | Northwestern University Feinberg School of Medicine Chicago, IL | 3 |
| Kent Choi / Patrick McGonagill | University of Iowa Hospital and Clinics Iowa CIty, IA | 6 |
| Gyorgy Frendl | Brigham & Women's Hospital Boston, MA | 2 |
| David Evans /Jonathan Wisler | The Ohio State University Wexner Medical Center Columbus, OH | 5 |
| Eyad Almasri | University of California San Francisco Fresno, CA | 1 |
| Jana Hudcova | Lahey Hospital & Medical Center Burlington, MA | 1 |
