# Supplementary material for: A Randomized, Double-blind, Multicenter Trial Comparing Efficacy and Safety of Imipenem/Cilastatin/Relebactam Versus Piperacillin/Tazobactam in Adults With Hospital-acquired or Ventilator-associated Bacterial Pneumonia (RESTORE-IMI 2 Study)
Source: Clin Infect Dis. 2020 Aug 12;73(11):e4539–48. doi: 10.1093/cid/ciaa803 (PMC8662781; doi:10.1093/cid/ciaa803)
Supplement: ciaa803_suppl_Supplementary_Protocol [file ciaa803_suppl_supplementary_protocol.pdf]

Merck & Co., Inc. policy on posting of redacted study protocols on journal websites is described in Guidelines for Publication of Clinical Trials in the Scientific Literature on the www.merck.com website.

For publicly posted protocols, the Company redacts content that contains proprietary and private information.

This report may include approved and non-approved uses, formulations, or treatment regimens. The results reported may not reflect the overall profile of a product. Before prescribing any product mentioned in this report, healthcare professionals should consult local prescribing information for the product approved in their country.

Copyright © 2020 Merck Sharp & Dohme Corp., a subsidiary of Merck & Co., Inc. All Rights Reserved. Not for regulatory or commercial use.

THIS PROTOCOL AMENDMENT AND ALL OF THE INFORMATION RELATING TO IT ARE CONFIDENTIAL AND PROPRIETARY PROPERTY OF MERCK SHARP & DOHME CORP., A SUBSIDIARY OF MERCK & CO., INC., WHITEHOUSE STATION, NJ, U.S.A.

This protocol amendment is applicable only to Portugal, Russia, United States.

SPONSOR:

Merck Sharp & Dohme Corp., a subsidiary of Merck & Co., Inc.
(hereafter referred to as the Sponsor or Merck)
One Merck Drive
P.O. Box 100
Whitehouse Station, NJ 08889-0100, U.S.A.

Protocol-specific Sponsor Contact information can be found in the Investigator Trial File Binder (or equivalent).

TITLE:

A Phase III, Randomized, Double-Blind, Active Comparator-Controlled Clinical Trial to Study the Safety, Tolerability, and Efficacy of Imipenem/Cilastatin/Relebactam (MK-7655A) Versus Piperacillin/Tazobactam in Subjects with Hospital-Acquired Bacterial Pneumonia or Ventilator-Associated Bacterial Pneumonia

IND NUMBER: [108,754]
EudraCT NUMBER: [2015-000246-34]

TABLE OF CONTENTS

SUMMARY OF CHANGES 10

1.0 TRIAL SUMMARY 13

2.0 TRIAL DESIGN 14

2.1 Trial Design 14

2.1.1 Early Consent Option 15

2.1.2 Standard Consent Option 15

2.2 Trial Diagram 16

3.0 OBJECTIVE(S) & HYPOTHESIS(ES) 17

3.1 Primary Objective(s) & Hypothesis(es) 17

3.2 Secondary Objective(s) & Hypothesis(es) 17

3.2.1 Key Secondary Objective 17

3.2.2 Other Secondary Objectives 17

3.3 Exploratory Objectives 18

4.0 BACKGROUND & RATIONALE 18

4.1 Background 18

4.1.1 Pharmaceutical and Therapeutic Background 19

4.1.2 Ongoing Clinical Trials 21

4.1.3 Information on Other Trial-Related Therapy 21

4.1.3.1 Comparator Therapy 21

4.1.3.2 Co-administration of Open-label Therapy with Linezolid for MRSA 21

4.2 Rationale 22

4.2.1 Rationale for the Trial and Selected Subject Population 22

4.2.2 Rationale for Dose Selection/Regimen/Modification 23

4.2.2.1 IMI (500 mg)/REL (250 mg) 23

4.2.2.2 Comparator –PIP (4000 mg)/TAZ (500 mg) 25

4.2.2.3 Duration of Therapy 25

4.2.2.4 Rationale for the Use of Comparator 25

4.2.3 Rationale for Endpoints 26

4.2.3.1 Efficacy Endpoints 26

4.2.3.1.1 Definition of Efficacy Endpoints 27
4.2.3.1.1.1 Survival/All-cause Mortality 28
4.2.3.1.1.2 Clinical Response 28
4.2.3.1.1.3 Microbiological Response 30
4.2.3.2 Safety Endpoints 33
4.2.3.3 Pharmacokinetic Endpoints 34
4.2.3.4 Planned Exploratory Biomarker Research 34
4.2.3.5 Future Biomedical Research 34
4.3 Benefit/Risk 34
5.0 METHODOLOGY 35
5.1 Entry Criteria 35
5.1.1 Diagnosis/Condition for Entry into the Trial 35
5.1.2 Subject Inclusion Criteria 35
5.1.3 Subject Exclusion Criteria 38
5.2 Trial Treatment(s) 41
5.2.1 Dose Selection/Modification 43
5.2.1.1 Dose Selection (Preparation) 43
5.2.1.1.1 IMI/REL 43
5.2.1.1.2 PIP/TAZ 43
5.2.2 Timing of Dose Administration 44
5.2.3 Trial Blinding/Masking 45
5.3 Randomization or Treatment Allocation 45
5.4 Stratification 45
5.5 Concomitant Medications/Vaccinations (Allowed & Prohibited) 46
5.6 Rescue Medications & Supportive Care 46
5.7 Diet/Activity/Other Considerations 46
5.8 Subject Withdrawal/Discontinuation Criteria 47
5.9 Subject Replacement Strategy 48
5.10 Beginning and End of the Trial 48
5.11 Clinical Criteria for Early Trial Termination 48
6.0 TRIAL FLOW CHART 49

7.0 TRIAL PROCEDURES ... 54
7.1 Trial Procedures ... 54
7.1.1 Administrative Procedures ... 54
7.1.1.1 Informed Consent ... 54
7.1.1.1.1 General Informed Consent ... 54
7.1.1.1.2 Consent and Collection of Specimens for Future Biomedical Research ... 55
7.1.1.2 Inclusion/Exclusion Criteria ... 55
7.1.1.3 Subject Identification Card ... 55
7.1.1.4 Medical History ... 55
7.1.1.5 Prior and Concomitant Medications Review ... 55
7.1.1.5.1 Prior Medications ... 55
7.1.1.5.2 Concomitant Medications ... 56
7.1.1.6 Assignment of Screening Number ... 56
7.1.1.7 Assignment of Randomization Number ... 56
7.1.1.8 Trial Compliance (Medication/Diet/Activity/Other) ... 57
7.1.1.9 Study Therapy Administration ... 57
7.1.1.10 Linezolid Therapy Administration ... 58
7.1.2 Clinical Procedures/Assessments ... 58
7.1.2.1 APACHE II Score ... 58
7.1.2.2 Clinical Pulmonary Infection Score (CPIS) ... 58
7.1.2.3 Full and Directed Physical Examinations ... 58
7.1.2.4 Vital Signs ... 58
7.1.2.5 Height and Weight ... 59
7.1.2.6 Adverse Event Monitoring and Local Infusion Tolerability Monitoring ... 59
7.1.2.7 Clinical Signs and Symptoms ... 59
7.1.2.8 Chest X-Ray ... 60
7.1.2.9 PaO_2/FiO_2 OR O_2 Saturation ... 61
7.1.2.10 Infection Source Control Review ... 61
7.1.3 Laboratory Procedures/Assessments ... 61
7.1.3.1 Laboratory Safety Evaluations (Hematology, Chemistry and Urinalysis) ... 62
7.1.3.2 Culture and Susceptibility Testing ... 63
7.1.3.2.1 Lower Respiratory Tract Sample Collected at Baseline ... 63

7.1.3.2.2 Other Lower Respiratory Tract Samples Following Randomization ... 64
7.1.3.2.3 Blood Cultures ... 64
7.1.3.2.4 Other Relevant Culture Samples ... 65
7.1.3.3 Pharmacokinetic/Pharmacodynamic Evaluations ... 65
7.1.3.3.1 Blood Collection for Plasma for REL, Imipenem, and Cilastatin ... 65
7.1.3.4 Planned Genetic Analysis Sample Collection ... 65
7.1.3.5 Future Biomedical Research ... 65
7.1.3.6 Efficacy Evaluation ... 66
7.1.3.6.1 Survival Assessment ... 66
7.1.3.6.2 Clinical Response ... 66
7.1.3.6.3 Microbiological Response ... 66
7.1.3.6.4 Disease-Related Complications ... 66
7.1.3.6.5 Health Resource Utilization Measures ... 67
7.1.3.6.5.1 Length of Stay in Hospital ... 67
7.1.3.6.5.2 Duration of Mechanical Ventilation ... 67
7.1.3.6.5.3 ICU Length of Stay ... 67
7.1.4 Other Procedures ... 67
7.1.4.1 Withdrawal/Discontinuation ... 67
7.1.4.1.1 Withdrawal From Future Biomedical Research ... 67
7.1.4.2 Blinding/Unblinding ... 68
7.1.4.3 Calibration of Equipment ... 68
7.1.5 Visit Requirements ... 68
7.1.5.1 Screening ... 69
7.1.5.2 Treatment Period ... 69
7.1.5.3 Post-Therapy ... 70
7.2 Assessing and Recording Adverse Events ... 70
7.2.1 Definition of an Overdose for This Protocol and Reporting of Overdose to the Sponsor ... 71
7.2.2 Reporting of Pregnancy and Lactation to the Sponsor ... 71
7.2.3 Immediate Reporting of Adverse EventsAdverse Events and Incidents to the Sponsor ... 72
7.2.3.1 Serious Adverse EventsAdverse Events and Incidents ... 72
7.2.3.2 Events of Clinical Interest ... 73

7.2.4 Evaluating Adverse Events 74
7.2.5 Sponsor Responsibility for Reporting Adverse Events 77
7.3 TRIAL GOVERNANCE AND OVERSIGHT 77
7.3.1 Scientific Advisory Committee 77
7.3.2 Executive Oversight Committee 77
7.3.3 Data Monitoring Committee 77
8.0 STATISTICAL ANALYSIS PLAN 78
8.1 Statistical Analysis Plan Summary 78
8.2 Responsibility for Analyses/In-House Blinding 80
8.3 Hypotheses/Estimation 80
8.4 Analysis Endpoints 80
8.4.1 Efficacy Endpoints 81
8.4.2 Safety Endpoints 81
8.5 Analysis Populations 82
8.5.1 Efficacy Analysis Populations 82
8.5.2 Safety Analysis Populations 83
8.6 Statistical Methods 83
8.6.1 Statistical Methods for Efficacy Analyses 83
8.6.2 Statistical Methods for Safety Analyses 84
8.6.3 Summaries of Baseline Characteristics, Demographics, and Other Analyses 86
8.7 Interim Analyses 86
8.8 Multiplicity 88
8.9 Sample Size and Power Calculations 88
8.10 Subgroup Analyses and Effect of Baseline Factors 89
9.0 LABELING, PACKAGING, STORAGE AND RETURN OF CLINICAL SUPPLIES 89
9.1 Investigational Product 89
9.2 Packaging and Labeling Information 90
9.3 Clinical Supplies Disclosure 90
9.4 Storage and Handling Requirements 90
9.5 Discard/Destruction/Returns and Reconciliation 90
9.6 Standard Policies 91

10.0 ADMINISTRATIVE AND REGULATORY DETAILS 91
10.1 Confidentiality 91
10.1.1 Confidentiality of Data 91
10.1.2 Confidentiality of Subject Records 91
10.1.3 Confidentiality of Investigator Information 91
10.1.4 Confidentiality of IRB/IEC Information 92
10.2 Compliance with Financial Disclosure Requirements 92
10.3 Compliance with Law, Audit and Debarment 92
10.4 Compliance with Trial Registration and Results Posting Requirements 94
10.5 Quality Management System 95
10.6 Data Management 95
10.7 Publications 95
11.0 LIST OF REFERENCES 97
12.0 APPENDICES 99
12.1 Merck Code of Conduct for Clinical Trials 99
12.2 Collection and Management of Specimens for Future Biomedical Research 101
12.3 Pharmacogenetics Informational Brochure for IRBs/IECs & Investigational Site Staff 107
12.4 Approximate Blood/Tissue Volumes Drawn/Collected by Trial Visit and by Sample Types 116
12.5 APACHE II Severity of Disease Classification System – APACHE II Score Form 117
12.6 Clinical Pulmonary Infection Score (CPIS) 119
12.7 List of Abbreviations 120
13.0 SIGNATURES 122
13.1 Sponsor's Representative 122
13.2 Investigator 122

LIST OF TABLES

Table 1 Summary of Efficacy Endpoints and Components of a Favorable Response 27
Table 2 Definitions of the Clinical Response Rating at the OTX Visits (Day 3, Day 6 and Day 10 of IV Study Therapy) 28
Table 3 Definitions of the Clinical Response Rating at the EOT Visit 29
Table 4 Definitions of the Clinical Response Rating at the EFU Visit and Day 28 Post-Randomization Visit 30
Table 5 Definitions of the By-Pathogen Microbiological Response Rating at the EOT Visit 32
Table 6 Definitions of the By-Pathogen Microbiological Response at the EFU Visit 33
Table 7 Trial Treatment 41
Table 8 Administration Dosage of IMI/REL According to Renal Function 43
Table 9 Recommended Dosage of PIP/TAZ in Subjects According to Renal Function (Total PIP/TAZ dosage provided first, followed by individual components in parentheses 44
Table 10 Infection-Site Clinical Signs and Symptoms 60
Table 11 Laboratory Tests 62
Table 12 Evaluating Adverse Events 75
Table 13 Summary of Analysis Strategy for Primary and Key Secondary Efficacy Endpoints 84
Table 14 Analysis Strategy for Safety Parameters 86
Table 15 Sample Size Adjustments based on Interim Blinded Review of Day 28 All-Cause Mortality 87
Table 16 Product Descriptions 89

LIST OF FIGURES

Figure 1 Trial Design 16

SUMMARY OF CHANGES

PRIMARY REASON FOR THIS AMENDMENT:

Section Number (s)	Section Title(s)	Description of Change (s)	Rationale
4.2.3.1.1.3<br>8.6.1	Microbiological Response<br>Statistical Methods for Efficacy Analyses	Added a fifth Indeterminate response option: e) No specimen taken because subject is deemed clinically cured or improved. Added/edited associated footnotes b and c linking this new indeterminate response category to "Presumed Eradication" in Tables 5 and 6 Definitions of Microbiological Response at the End-of-Therapy (EOT) and Early Follow-up (EFU) Visits, respectively, and added text description of this option for the efficacy analyses.<br>This update is in alignment with the way the data are already being collected in the study.	This change was made to provide an additional option of indeterminate microbiological response so that a "presumed eradication" outcome may be derived based on patients who are clinically cured or improved and from whom a respiratory specimen could not be obtained. The outcome of "presumed eradication" will be considered a favorable overall microbiological response in the efficacy analyses.

ADDITIONAL CHANGE(S) FOR THIS AMENDMENT:

**Section Number (s)**	**Section Title (s)**	**Description of Change (s)**	**Rationale**
2.1<br>5.4<br>7.1.1.7<br><br>8.1	Trial Design<br>Stratification<br>Assignment of Randomization Number<br>Statistical Analysis Plan Summary - Treatment Assignment	The randomization requirement was modified to allow for "approximately" 50% of subjects with ventilated HABP or VABP.	This change was made to allow flexibility in the number and proportion of ventilated HABP or VABP subjects randomized.
5.1.3	Subject Exclusion Criteria<br>Exclusion #1<br>Exclusion #12	Added list of acceptable Gram stain patterns to clarify what constitutes an exclusionary pattern.<br>Added a note clarifying that the use of seizure prophylaxis among patients with no active or prior history of seizure disorder is acceptable for the specified uses, considering Exclusion #12.	These clarifications were added to better define these exclusion criteria.
5.5	Concomitant Medications/Vaccinations (Allowed & Prohibited)	Clarified that medications specifically prohibited in the protocol are not allowed during IV study therapy, rather than during the whole study.	This text was updated to align with current Merck standard protocol template.
6.0<br><br>7.1.3.3	Trial Flow Chart - Whole blood to obtain plasma for REL, imipenem, and cilastatin assay, Footnote "t"<br>Pharmacokinetic/ Pharmacodynamic Evaluations	Clarified the timing of PK sampling to post-"start of" Dose 3.	This text was added to clarify PK sample collection timing relative to the start of infusion.

Section Number (s)	Section Title (s)	Description of Change (s)	Rationale
7.1.3.2.2	Other Lower Respiratory Tract Samples Following Randomization	Removed the acceptable sputum sample criterion for post-baseline specimens of "greater than 25 neutrophils on low power microscopy review of the Gram stain".	This text was removed; it had been included in error in the previous protocol version.
7.1.4.2	Blinding/Unblinding	Updated the text on participant blinding/unblinding for blinded trials (IVRS/IWRS) using the emergency unblinding call center	This text was updated to align with the Merck standard protocol template language in the latest general protocol amendment (-014-04) for this study.
7.1.4.3	Calibration of Equipment	Removed "critical" throughout and deleted the list of equipment.	This text was updated to align with current Merck standard protocol template.
9.3	Clinical Supplies Disclosure	Updated the text for blinded trials (IVRS/IWRS) using the emergency unblinding call center with no disclosure envelopes provided.	This text was updated to align with current Merck standard protocol template.
8.6.1	Statistical Methods for Efficacy Analyses	Added specification that Cochran-Mantel-Haenszel (CMH) weights will be used for efficacy analyses by baseline pneumonia type and APACHE II score.	This text was added to provide the methods to be used for the stratified efficacy analyses.

1.0 TRIAL SUMMARY

Abbreviated Title	IMI/REL (MK-7655A) vs. PIP/TAZ for Treatment of Subjects with HABP/VABP
Trial Phase	III
Clinical Indication	Treatment for hospital-acquired/ventilator-associated bacterial pneumonia (HABP/VABP)
Trial Type	Interventional
Type of control	Active control
Route of administration	Intravenous
Trial Blinding	Double-blind
Treatment Groups	Treatment Group 1: Imipenem/cilastatin/relebactam (IMI/REL) administered intravenously (IV) as a fixed-dose combination<br>Treatment Group 2: Piperacillin/tazobactam (PIP/TAZ) administered IV as a fixed-dose combination.<br>In both treatment groups, the use of initial empiric treatment with open-label IV linezolid (600 mg every 12 hours) for methicillin-resistant *S. aureus* (MRSA) infection is required and will be administered in an open-label fashion.
Number of trial subjects	Approximately 536 subjects will be enrolled.
Estimated duration of trial	The sponsor estimates that the trial will require approximately 25 months [(~ 107 weeks) of enrollment + up to 14 days (2 weeks) of IV study therapy + at least 14 days (2 weeks) of follow-up = 111 weeks] from the time the first subject signs the informed consent until the last subject's last visit.
Duration of Participation	Subjects who are consented under the standard consent option will participate in the trial for approximately 28 to 31 days from the time the subject signs the Informed Consent Form (ICF) through the final contact. After a screening visit, each subject will be receiving assigned treatment for approximately 7 to up to 14 days. After the end of treatment each subject will be followed for 14 days. All subjects must also have a study visit at 28 days following randomization. The total duration for each subject in the study will be up to 31 days.<br>Subjects who are consented under the early consent option will participate in the trial for approximately 42 to 45 days due to a prolonged screening period of up to 14 days. The total duration for these subjects will be up to 45 days.
Randomization Ratio	1:1 for Treatment Group 1 IMI/REL: Treatment Group 2 PIP/TAZ

A list of abbreviations used in this document can be found in Section 12.7.

2.0 TRIAL DESIGN

2.1 Trial Design

This is a randomized, double-blind (with in-house blinding), active-controlled, parallel-group, multi-site trial of imipenem/cilastatin/relebactam (also known as MK-7655A; hereafter referred to as IMI/REL) compared with piperacillin/tazobactam (PIP/TAZ) in subjects with hospital-acquired bacterial pneumonia (HABP) or ventilator-associated bacterial pneumonia (VABP). Subjects with HABP may either be ventilated (ventilated HABP) or non-ventilated (non-ventilated HABP). There are 2 consent options for this trial: an early consent option (Section 2.1.1) and a standard consent option (Section 2.1.2).

Approximately 536 subjects will be randomized in a 1:1 ratio to one of two treatment arms of the study, Treatment Group 1 (IMI/REL) or Treatment Group 2 (PIP/TAZ). Both treatments will be provided as fixed-dose combinations, administered intravenously (IV) every six hours. Subjects who are consented under the early consent option will have a maximum 14-day screening period following signing of consent. Subjects who are consented under the standard consent option will receive study treatment after a maximum 48-hour screening period following signing of consent. All randomized subjects in each treatment group will receive a minimum of 7 days to up to a maximum of 14 days of IV study therapy. Subjects with evidence of concurrent bacteremia or with *P. aeruginosa* infection should receive 14 days of IV study therapy. While on IV study therapy, study visits will be performed on Day 1 (randomization), Day 3 (on-therapy visit #1, OTX1), Day 6 (on-therapy visit #2, OTX2), Day 10 (on-therapy visit #3, OTX3, if applicable) and at the end of therapy (EOT). Following the completion of IV study therapy, all subjects will have a study visit 7 to 14 days following completion of therapy (at the early follow-up visit, EFU). In addition, a Day 28 post-randomization visit will be performed in all subjects (this visit may be performed on the same day as the EFU visit, depending on the duration of IV study therapy). All subjects will remain in the study for a total of up to 31 days after study randomization.

Since the study will enroll subjects without confirmed microbiological (culture) evidence of the HABP/VABP pathogen from a lower respiratory tract specimen at study entry, the use of initial empiric treatment with open-label IV linezolid (600 mg every 12 hours [q12h]) for methicillin-resistant *S. aureus* (MRSA) infection is required. The recommended treatment duration for a confirmed MRSA infection is a minimum of 7 days, with a minimum of 14 days for MRSA bacteremia. Empiric linezolid treatment will be stopped if the final culture results from the baseline lower respiratory tract sample do not demonstrate the presence of MRSA.

Randomization will be stratified based on pneumonia type (non-ventilated HABP vs. ventilated HABP/VABP) and Acute Physiology and Chronic Health Evaluation II (APACHE II) score at baseline ($<$ 15 vs. $\geq$ 15). Approximately 50% of subjects will have ventilated HABP or VABP.

Safety and tolerability will be carefully monitored throughout the study by an external Data Monitoring Committee (eDMC).

2.1.1 Early Consent Option

An early consent option is permitted for subjects who are considered at risk by the investigator for developing VABP but have not yet exhibited any or all of the clinical or radiographic signs or symptoms of VABP when they enter the study. Subjects who provide early consent must meet the following 3 early screening criteria and then have up to 2 weeks to fulfill all remaining inclusion and exclusion criteria and be randomized to receive study medication:

- are 18 years or older on the day of signing informed consent (ie, Inclusion Criterion #1),
- are anticipated to require extended intubation based on the opinion of the investigator, which can potentially put the subject at risk of developing VABP (ie, Inclusion Criterion #9), and
- understand (or have a legal representative that understands) the study procedures, alternative treatments available, and risks involved with the study, and voluntarily agree to participate by giving written informed consent for the trial (ie, Inclusion Criterion #7).

If these 3 early screening criteria are fulfilled and the subject goes on to develop signs and symptoms of pneumonia, the subject will qualify to be assessed for disease eligibility. If disease eligibility and all other study entry criteria are met, the subject may be randomized within 48 hours after meeting study entry criteria and will proceed into the study.

2.1.2 Standard Consent Option

The standard consent option is for subjects who meet all study entry criteria at the time of providing consent. These subjects will be randomized within 48 hours after meeting study entry criteria.

Specific procedures to be performed during the trial, as well as their prescribed times and associated visit windows, are outlined in the Trial Flow Chart - Section 6.0. Details of each procedure are provided in Section 7.0 – Trial Procedures.

2.2 Trial Diagram

The trial design is depicted in Figure 1.

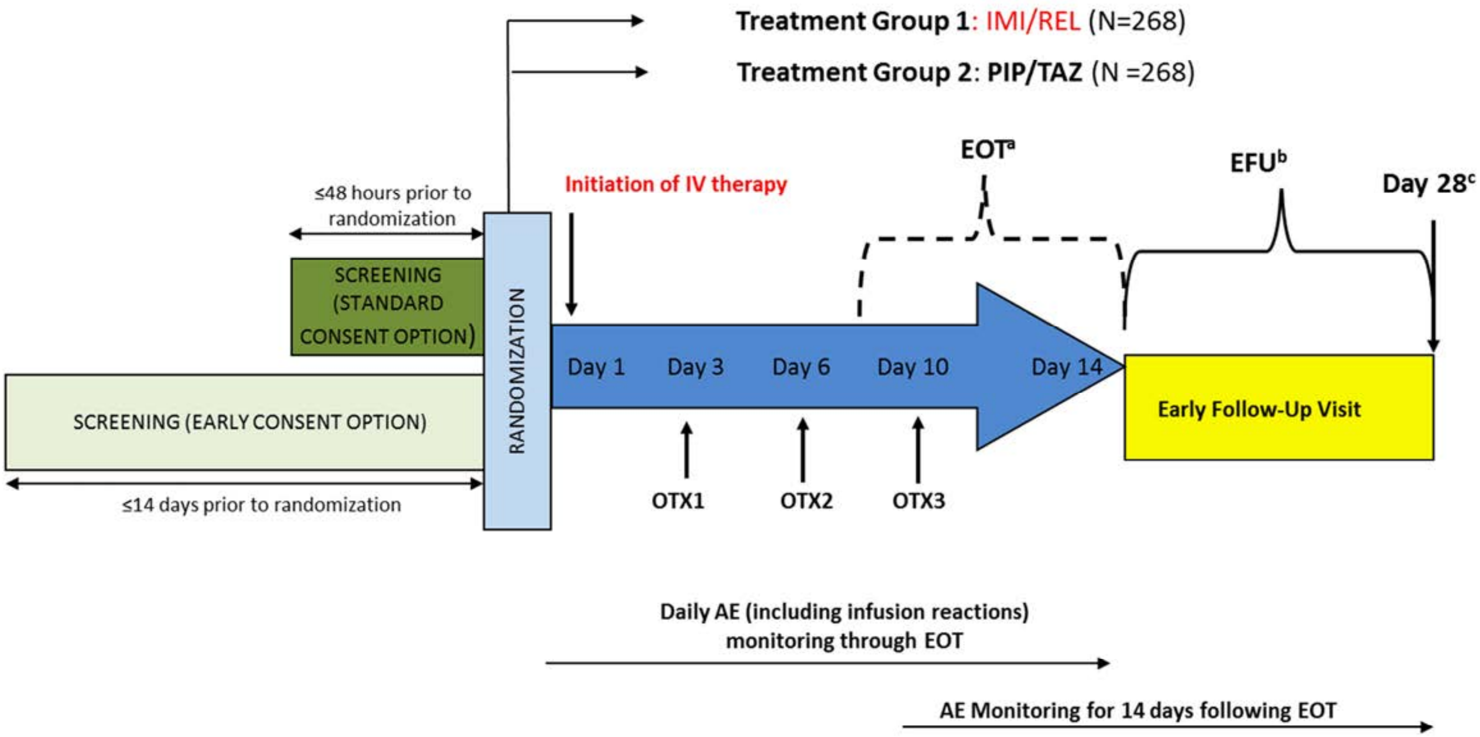

OTX= on therapy, **EOT**= end of therapy; **EFU**= early follow-up , **Day 28** = Day 28 post-randomization.
[a] The EOT visit must occur ≤24 hours after the last dose of IV study therapy. Minimum duration of IV therapy is 7 full days. Maximum duration must not exceed 14 days. Subjects with bacteremia or with *Pseudomonas aeruginosa* infection will receive 14 days of IV therapy.
[b] 7 to 14 days (up to an additional 2 days) following EOT. The EFU and Day 28 visits may be combined as long as compliance with the visit windows is maintained for both visits.
[c] 28 days (up to an additional 3 days) following randomization.

Figure 1 Trial Design

3.0 OBJECTIVE(S) & HYPOTHESIS(ES)

3.1 Primary Objective(s) & Hypothesis(es)

1) **Objective:** To determine the incidence rate of all-cause mortality through Day 28 post-randomization associated with treatment with IMI/REL compared to treatment with PIP/TAZ in subjects diagnosed with HABP/VABP in the modified intention-to-treat (MITT) population.

   Hypothesis: IMI/REL is non-inferior to PIP/TAZ in subjects with HABP/VABP, as measured by the incidence rate of all-cause mortality through Day 28 post-randomization in the MITT population. A non-inferiority margin of 10% will be used. Further details for determining non-inferiority are described in Section 8.0 (Statistical Analysis Plan). If non-inferiority is established, the following will be evaluated: IMI/REL is superior to PIP/TAZ in subjects with HABP/VABP, as measured by the incidence rate of all-cause mortality through Day 28 post-randomization in the MITT population.

3.2 Secondary Objective(s) & Hypothesis(es)

3.2.1 Key Secondary Objective

1) **Objective**: To evaluate the efficacy of IMI/REL versus PIP/TAZ with respect to clinical response at the early follow-up (EFU) visit 7 to 14 days after the end of therapy (EOT) for subjects diagnosed with HABP/VABP in the MITT population.

   Hypothesis: IMI/REL is non-inferior to PIP/TAZ with respect to the proportion of subjects with a favorable clinical response at the EFU visit in subjects with HABP/VABP in the MITT population. A non-inferiority margin of 12.5% will be used. Further details for determining non-inferiority are described in Section 8.0 (Statistical Analysis Plan). If non-inferiority is established, the following will be evaluated: IMI/REL is superior to PIP/TAZ with respect to the proportion of subjects with a favorable clinical response at the EFU visit in subjects with HABP/VABP in the MITT population.

3.2.2 Other Secondary Objectives

1) **Objective**: To evaluate the safety and tolerability profile of IMI/REL vs. PIP/TAZ in subjects with HABP/VABP.

2) **Objective**: To determine the incidence rate of all-cause mortality through Day 28 post-randomization for IMI/REL versus PIP/TAZ in subjects diagnosed with HABP/VABP in the microbiological modified intention-to-treat (mMITT) population.

3) **Objective**: To determine the incidence rate of all-cause mortality at the EFU visit for IMI/REL versus PIP/TAZ in subjects diagnosed with HABP/VABP in the MITT population and mMITT population.

4) **Objective**: To determine the incidence rate of all-cause mortality through Day 28 post-randomization in subjects treated with IMI/REL versus PIP/TAZ based on pneumonia type (non-ventilated HABP, ventilated HABP/VABP) in the MITT population and mMITT population.
5) **Objective**: To evaluate the efficacy of IMI/REL versus PIP/TAZ with respect to clinical response at the EFU visit for subjects diagnosed with HABP/VABP in the clinically evaluable (CE) population.
6) **Objective**: To evaluate the efficacy of IMI/REL versus PIP/TAZ with respect to clinical response on Day 3 of IV study therapy (OTX1), Day 6 of IV study therapy (OTX2), Day 10 of IV study therapy (OTX3, if applicable), at the EOT visit, and at Day 28 post-randomization for subjects diagnosed with HABP/VABP in the MITT population and CE population.
7) **Objective**: To evaluate the efficacy of IMI/REL versus PIP/TAZ with respect to microbiological response at the EOT visit and at the EFU visit for subjects diagnosed with HABP/VABP in the mMITT population and microbiologically evaluable (ME) population.

3.3 Exploratory Objectives

1) **Objective:** To summarize the pharmacokinetics of relebactam, imipenem, and cilastatin and to evaluate the pharmacokinetic-pharmacodynamic (PK/PD) association of relebactam and imipenem.
2) **Objective:** To determine the incidence of all-cause mortality and disease-related complications (e.g., development of empyema; onset of acute respiratory distress syndrome; bacteremia) through Day 28 post-randomization in subjects treated with IMI/REL versus PIP/TAZ in the MITT population.
3) **Objective**: To evaluate the efficacy of IMI/REL versus PIP/TAZ with respect to the following key health resource utilization measures: (a) number of days spent in the hospital post-randomization (i.e., length of stay [LOS]) in the MITT population and mMITT population; (b) the number of days spent on mechanical ventilation post-randomization for the subset of subjects with VABP and ventilated HABP in the MITT population and mMITT population; (c) the number of days spent in the intensive care unit (ICU) post-randomization for the subset of subjects with VABP and ventilated HABP in the MITT population and mMITT population.
4) **Objective:** To explore the relationship between genetic variation and response to the treatment(s) administered. Variation across the human genome will be analyzed for association with clinical data collected in this study.

4.0 BACKGROUND & RATIONALE

4.1 Background

Refer to the MK-7655 Investigator's Brochure (IB) for detailed background information on MK-7655A

4.1.1 Pharmaceutical and Therapeutic Background

Relebactam (REL, MK-7655) is a parenteral (IV), small-molecule β-lactamase inhibitor (BLI) which is being developed as a fixed-dose combination in a single vial with imipenem/cilastatin (referred to as IMI) for the treatment of infections caused by gram-negative bacteria. Throughout this document the fixed-dose combination (MK-7655A) of imipenem/cilastatin (IMI) + REL will be referred to as IMI/REL.

β-lactam antibiotics (penicillins, cephalosporins, carbapenems, and monobactams) are among the most frequently used antimicrobial agents in clinical practice. The unrelenting development of resistance to β-lactam antibiotics by the production of β-lactamases is the most important resistance mechanism among gram-negative bacteria and poses an ongoing threat to the clinical utility of all β-lactams. Therefore, there is an urgent need for new BLIs that can be combined with existing β-lactam antibiotics to protect against hydrolysis by one or more of the four classes (A, B, C and D) of β-lactamase enzymes.

IMI, a potent broad spectrum β-lactam antibacterial agent from the carbapenem class, has been used clinically for the treatment of serious infections since 1985. The bactericidal activity of imipenem results from inhibition of cell wall synthesis. Imipenem, when administered alone, is metabolized in the kidneys by dehydropeptidase I, resulting in relatively low levels in the urine. Cilastatin sodium is an inhibitor of this enzyme and effectively prevents renal metabolism of imipenem so that, when given together, adequate antibacterial levels of imipenem are achieved. Imipenem is active against a broad range of gram-positive and gram-negative organisms and is approved for use in a variety of infections, including lower respiratory tract infections, urinary tract infections (complicated and uncomplicated), intra-abdominal infections, gynecologic infections, bacterial septicemia, bone and joint infections, skin and skin structure infections, endocarditis, and polymicrobial infections. REL represents a new generation of BLIs to combat evolving clinical resistance and to maintain the usefulness of the β-lactam class of antibiotics. REL is a dual Ambler Class A/Class C BLI that is highly potent against AmpC, a common Class C β-lactamase encountered in many bacteria, most predominantly *Pseudomonas aeruginosa*. REL is also active against the Class A β-lactamases, including the *Klebsiella pneumoniae* carbapenemase (KPC) present in some Enterobacteriaceae, including *Klebsiella* strains. REL has no activity against the Class B metallo-β-lactamases (including NDM-1, IMP, or VIM-containing strains) or Class D β-lactamases (including OXA-producing strains).

Preclinical data, including *in vitro* microbiological studies with imipenem-resistant clinical isolates of *P. aeruginosa* and KPC-producing organisms, as well as *in vivo* infection models with imipenem-resistant *P. aeruginosa* and *K. pneumoniae*, suggest that REL, in combination with IMI, has the potential to fulfill a significant and growing medical need by providing a next-generation BLI to combat severe gram-negative bacterial infections. Preclinical toxicity studies in rats and monkeys have demonstrated that REL is generally well-tolerated. There was no evidence of adverse effects of REL as a single agent on cardiovascular, central nervous system, and respiratory function in well-characterized preclinical safety pharmacology models. Toxicity of REL in combination with IMI has been evaluated for up to one month in monkeys with no adverse effects noted at 1.3 times the targeted human exposure of 344.4 uM.hr. Evidence of renal toxicity was observed when MK-7655 was administered alone at levels 8 times the target human exposure; however, no clinically

relevant findings associated with renal function have been identified in the Phase I studies or in the completed Phase II study in humans.

As of 30-Nov-2017, REL has been evaluated in approximately 265 individuals, 232 of whom have received at least one dose of REL, across seven completed Phase I studies (PN001, PN002, PN005, PN007, PN009, PN012, and PN019). Healthy young and elderly male and female adults as well as patients with varying degrees of renal insufficiency have been studied, including patients with end stage renal disease (ESRD) on hemodialysis.

Unblinded safety data from the Phase I studies have demonstrated that single and multiple intravenous doses of REL have been generally safe and well tolerated throughout the dose ranges tested. In PN001, generally mild elevations in hepatic transaminases above the upper limit of normal range (ULN) have been observed in the multiple-dose treatment arms in which REL was co-administered with IMI. Elevations were also seen in subjects receiving IMI alone. None of the liver transaminase elevations in these subjects were associated with clinical findings. The elevations were not dose related and were reversible after discontinuation of dosing. Elevations have not been observed in subjects administered single or multiple doses in subsequent Phase I or Phase II trials.

The pharmacokinetics of REL, imipenem, and cilastatin were evaluated following single and multiple doses of REL in combination with 500 mg IMI, administered every 6 hours for 7 to 14 days in PN001 and PN002. Data from these studies demonstrated that REL exposures increase proportionally with dose, doses at 125 mg and above exceeding the identified REL PK target of $AUC_{0-\infty} \geq 37.5$ μM.hr. The pharmacokinetics of REL, imipenem, and cilastatin were also evaluated in renally impaired subjects (PN005). Pharmacokinetic data from PN005 were consistent with expectations given that REL, imipenem and cilastatin are cleared almost entirely renally in healthy subjects. The plasma clearance (CL_{plasma}), terminal half-life ($t_{1/2}$) and area under the concentration time curve ($AUC_{0-\infty}$) all were significantly and similarly altered for each of these three analytes when comparing subjects with renal impairment to their healthy matched subjects. These data are consistent with the expected change in magnitude of glomerular filtration rate (GFR). In addition, in subjects with ESRD, REL, imipenem and cilastatin were efficiently removed by hemodialysis. The pharmacokinetics of REL was also studied in healthy volunteers in an intrapulmonary lung penetration study (PN007). In PN007, the intrapulmonary pharmacokinetic profiles of REL and imipenem were assessed after administration of REL and IMI administered every 6 hours over 5 doses. Data in these subjects showed comparable penetration of REL and imipenem into the epithelial lung fluid (ELF), with relative exposures (AUC_{0-inf} in ELF compared to plasma) based on mean profiles of 43% for REL and 42% for IMI. In PN009, a thorough QTc study in 36 healthy subjects, a supratherapeutic dose of REL did not prolong the QTcP interval to a clinically significant degree.

Two Phase II comparator-controlled clinical trials of IMI + REL (Protocol 003 [PN003] and Protocol 004 [PN004]) have been completed. Both trials were randomized, double-blind, multicenter, comparative studies evaluating the safety, tolerability, and efficacy of IMI + REL versus IMI alone in 302 adults with complicated urinary tract infection (cUTI, PN003) and 351 adults with complicated intra-abdominal infection (cIAI, PN004). In both trials, subjects were randomized in a 1:1:1 ratio to one of 3 treatment groups (1) IMI (500 mg) + REL (250 mg), (2) IMI (500 mg) + REL (125 mg), or (3) IMI (500 mg) + placebo. The primary efficacy analysis for each study indicates that treatment with either dose of IMI +

REL is as effective as IMI alone as measured by the proportion of subjects with cUTI with a favorable microbiological response at completion of IV study therapy (DCIV, PN003) and the proportion of subjects with cIAI with a favorable clinical response at DCIV (PN004). Overall, both doses of REL (125 mg and 250 mg) were generally well tolerated.

Additional details regarding the preclinical, Phase I, and Phase II clinical studies completed to date are summarized in the MK-7655 IB.

4.1.2 Ongoing Clinical Trials

A Phase III clinical trial of IMI/REL has recently completed enrollment, and data are undergoing analysis as of 30-Nov-2017. PN013 is a randomized, double-blind, comparator-controlled study to estimate the safety and efficacy of IMI/REL versus colistin as colistimethate sodium (CMS) + IMI in subjects with imipenem-non-susceptible bacterial infections, including HABP/VABP, cIAI, and cUTI. Forty-seven subjects were randomized in a 2:1 ratio to receive one of the following double-blind treatments: 1) IMI/REL + placebo to CMS, or 2) CMS + IMI (500 mg). In addition, 3 subjects were enrolled into a third, open-label treatment arm in subjects with imipenem- and colistin-non-susceptible infections who received IMI/REL.

4.1.3 Information on Other Trial-Related Therapy

4.1.3.1 Comparator Therapy

Subjects in the comparator arm of this trial will receive intravenous (IV) infusions of piperacillin/tazobactam (PIP/TAZ).

PIP/TAZ is a combination product consisting of a penicillin-class antibacterial, piperacillin, and a β-lactamase inhibitor, tazobactam. It is indicated for the treatment of subjects with moderate to severe infections caused by susceptible gram-negative and gram-positive bacterial isolates: (β-lactamase producing isolates of *Escherichia coli*, *Staphylococcus aureus*, *Haemophilus influenzae*), members of the *Bacteriodes fragilis* group (*B. fragilis*, *B. ovatus*, *B. thetaiotaomicron*, or *B. vulgatus*), piperacillin/tazobactam-susceptible *Acinetobacter baumannii*, *Haemophilus influenzae*, *Klebsiella pneumoniae*, and *Pseudomonas aeruginosa*) for conditions such as intra-abdominal infections, skin and skin structure infections, female pelvic infections, community-acquired pneumonia and nosocomial pneumonia [1].

PIP/TAZ acts by inhibiting septum formation and cell wall synthesis of susceptible bacteria.

4.1.3.2 Co-administration of Open-label Therapy with Linezolid for MRSA

Beginning at randomization, all subjects will receive initial empiric treatment with IV linezolid (600 mg q12h) for methicillin-resistant *S. aureus* (MRSA) infection.

Linezolid is a synthetic antibacterial agent of a new class of antibiotics, the oxazolidinones. It is used for the treatment of infections caused by gram-positive bacteria. The *in vitro* spectrum of activity of linezolid also includes certain gram-negative bacteria and anaerobic bacteria. Linezolid is a bacterial protein synthesis inhibitor. Its mechanism of action is prevention of the formation of the ribosomal initiation complex thus affecting the translation

process. Results from time-kill studies have shown linezolid to be bacteriostatic against enterococci and staphylococci.

Linezolid is indicated for the treatment of the following infections caused by susceptible gram-positive bacteria: nosocomial pneumonia; community-acquired pneumonia; complicated skin and skin structure infections, including diabetic foot infections, without concomitant osteomyelitis; uncomplicated skin and skin structure infections; vancomycin-resistant *Enterococcus faecium* infections. [2]

Both linezolid or vancomycin are recommended for the treatment of MRSA infections when there is a proven or suspected infection due to MRSA, as per the American Thoracic Society (ATS)/Infectious Diseases Society of America (IDSA) guidelines on the treatment of HABP/VABP and the IDSA guidelines on treatment of MRSA infections. Of note, these guidelines recognize that linezolid might be preferred in certain situations, including (a) subjects with evidence of renal insufficiency due to potential for vancomycin under dosing in this patient population and the potential risk of nephrotoxicity associated with vancomycin; and (b) institutions where vancomycin resistance (i.e., MRSA isolates with vancomycin MIC ≥2 mcg/mL) is common. [3] [4]

Linezolid was compared to vancomycin for the treatment of nosocomial pneumonia due to MRSA in a randomized double-blind trial. In this study, the efficacy responses were numerically higher with linezolid than vancomycin. Overall, 58% of linezolid-treated patients and 47% of vancomycin-treated patients had achieved clinical cure at end of study. Additionally, 83% of linezolid-treated patients and 70% of vancomycin-treated patients had achieved clinical cure at the end of treatment. The incidence of documented microbiological persistence was also lower in the linezolid group, with 17% of linezolid-treated patients and 46% of vancomycin-treated patients having a positive culture result for MRSA at the end of treatment. The incidence of all-cause mortality at Day 60 was similar between treatment groups. Although the overall incidence of adverse events were similar in the 2 treatment groups, nephrotoxicity occurred more commonly on vancomycin than linezolid (18% vs. 8%, respectively). [5]

4.2 Rationale

4.2.1 Rationale for the Trial and Selected Subject Population

Details regarding specific benefits and risks for subjects participating in this clinical trial may be found in the accompanying MK-7655 Investigators Brochure (IB) and Informed Consent documents.

HABP and VABP are important problems in the care of critically ill hospitalized subjects despite advances in antimicrobial therapy, improvements in supportive care and the use of a wide range of preventative measures. In subjects with HABP, mortality rates are significant and range from 12-22% [6] [7] [8]. The risk of mortality increases approximately two-fold in subjects with hospital-acquired pneumonia associated with mechanical ventilation (VABP) and can be even higher when the lung infection is caused by multidrug-resistant (MDR) bacteria [9]. These infections are among the most frequently occurring infections in ICUs and account for a large majority of the antibacterial utilization within ICUs [6]. Appropriate antibiotic therapy significantly improves survival for subjects with HABP or VABP. In these vulnerable subjects, additional increases in morbidity and mortality can be attributed to the

empiric use of antimicrobial therapy that is not active against causative pathogens [10]. New, well-tolerated drugs with more definitive and rigorously tested dosing schemes showing proven activity against both susceptible and MDR bacteria without need for combination therapy are urgently needed, especially in subjects with HABP or VABP. IMI/REL has the potential to fulfill a significant and growing unmet medical need by providing a next generation BL/BLI with which to combat severe gram-negative bacterial infections.

The purpose of this study is to evaluate the efficacy and safety of IMI/REL with the intention of demonstrating that IMI/REL is non-inferior to PIP/TAZ in the treatment of adult subjects diagnosed with HABP or VABP. If non-inferiority is established, then superiority of IMI/REL compared to PIP/TAZ will be evaluated.

The primary endpoint for this study, all-cause mortality through Day 28 post-randomization, was selected based upon the expected large antibacterial treatment effect on survival. In this study, secondary endpoints will focus on the clinical response and microbiological response in an effort to provide additional information with regard to the effect of IMI/REL vs. PIP/TAZ in HABP/VABP subjects.

4.2.2 Rationale for Dose Selection/Regimen/Modification

4.2.2.1 IMI (500 mg)/REL (250 mg)

Based on data from *in vivo* animal models of imipenem-resistant gram-negative infections, the target pharmacokinetic (PK) parameter for REL has been defined as a plasma $AUC_{0\text{-}24hr}$ following four-times daily dosing of 150 µM*hr (or an $AUC_{0\text{-}\infty} \geq 37.5$ µM*hr following single dose administration). Extensive PK/PD *in vitro* and *in vivo* modeling work, together with multiple-dose safety data for REL from the Phase I program, supports doses of REL administered IV at or above 125 mg every 6 hours. However, preclinical microbiology data indicate that there are some highly resistant strains of *P. aeruginosa* that may require higher concentrations of REL. To this end, it is appropriate to target a safe dose of REL that exceeds the anticipated PK target in order to appropriately cover a broader range of resistant bacteria. This is particularly important in HABP/VABP infections as MDR gram-negative organisms are more common in these infections. REL doses that are associated with plasma exposures exceeding the PK target of REL, such as 250 mg administered IV once every 6 hours, would support this evaluation.

Per the ATS/IDSA treatment guidelines for nosocomial pneumonia, a recommended IMI dose for HABP/VABP is 500 mg every 6 hours. [3].

In order to assess drug penetration at the site of action (pulmonary penetration), an open-label study was conducted to evaluate the pharmacokinetics (PK) of REL and imipenem in the pulmonary epithelial lining fluid (ELF) and alveolar cells (AC) after administration of multiple doses of REL and IMI in 16 healthy young male or female subjects. The intrapulmonary pharmacokinetic profiles of REL and imipenem were assessed after subjects received multiple administrations of REL (250 mg) and IMI (500 mg) IV every 6 hours (q6h) over 5 doses. Penetration of both REL and imipenem into the epithelial lining fluid (ELF) was similar, with relative exposures ($AUC_{0\text{-}inf}$ in ELF compared to plasma) based on mean profiles of 43% for REL and 42% for imipenem. Thus, given the PK target based on plasma

PK was exceeded at the 125 mg dose, this target is also projected to be met in the ELF at the selected 250 mg clinical dose, accounting for differences in ELF versus plasma exposure.

Both 125 mg and 250 mg doses of REL have been evaluated in the Phase II clinical studies (PN003 and PN004). In order to inform the choice of dose for the current proposed study, results of an interim analysis of combined safety data from PN003 and PN004 were reviewed by a standing internal Data Management Committee (siDMC). The interim analysis included an evaluation of the safety and tolerability of the 250 mg dose of REL given in combination with IMI in comparison to the control regimen (IMI alone) and in comparison to the 125 mg dose of REL + IMI. The analysis was performed after 50% of planned subjects across PN003 and PN004 (N=331) were followed through the early follow-up visit (5 to 9 days following completion of study therapy). The data from the interim analysis supported the use of a 250 mg dose of REL with IMI. The safety profile of the 250 mg dose is further supported by unblinded data from PN004 which showed that the safety and tolerability profile of the 250 mg dose was similar to the 125 mg dose of REL as well as to IMI alone (See Section 4.1.1 and the MK-7655 IB for further details). The 250 mg dose was selected for this study to achieve exposures above the PK target that may be required for the treatment of highly resistant organisms.

As previously discussed, REL, imipenem and cilastatin are primarily renally excreted, with similar increases observed for each analyte in a Phase I renal insufficiency study (PN005). Thus, dose adjustments can be made in the same proportion for both REL and IMI, and all can be dosed in a fixed ratio. IMI and REL will be provided together in a single vial as a fixed-dose combination product in this study. Dose adjustment is required in subjects with renal impairment. Depending on the individual subject’s renal function (as determined by actual or estimated creatinine clearance), the total daily dose of IMI/REL may be adjusted. The IMI package circular also includes reduced daily doses for patients with lower body weight. However, the total daily dose for IMI-containing therapy (IMI/REL and IMI) for subjects in this study will not be adjusted for weight. This approach is consistent with general clinical practice and other recent clinical trials using IMI as a comparator. For example, clinical trials for HABP/VABP published since 2006 have administered IMI at standard dosages without an adjustment for weight. Efficacy rates in these trials have ranged from ~60 to 80% without significant toxicity [6] [11] [12] [13]. The few published clinical trials in which IMI dosage was adjusted for weight describe similar efficacy and safety [14] [15]. Furthermore, weight is already a component of calculated creatinine clearance, used for determination of renal function and need for dose adjustment, thus raising the theoretical concern of under-dosing in subjects with low body weight. Population PK-based simulations were conducted to evaluate the impact of removal of weight-based adjustments for IMI. Results of these simulations indicate that in individuals of lower weight, exposures of IMI/REL are significantly lower than those in higher weight ranges when doses are adjusted based on weight, and are supportive of dose adjustments based solely on renal function, as has been described above in clinical practice. Specifically, simulation results indicate that in subjects with normal renal function, the dose of 250 mg REL and 500 mg IMI given every 6 hours IV is appropriate. For subjects with mild renal insufficiency, the dose should be reduced to 200 mg REL and 400 mg IMI given every 6 hours IV; for moderate renal insufficiency, 150 mg REL and 300 mg IMI given every 6 hours IV; and for severe renal insufficiency, 100 mg REL and 200 mg IMI given every 6 hours IV. These doses result in a

consistent percentage of subjects achieving the PK targets for both REL and IMI across the range of weights and creatinine clearances, and maintain exposures in a range demonstrated to be safe and well-tolerated for both IMI and REL. The specific dosing guidelines are included in Section 5.2.1.1.

4.2.2.2 Comparator –PIP (4000 mg)/TAZ (500 mg)

The chosen dose for PIP/TAZ in this study is 4500 mg (4000 mg piperacillin/ 500 mg tazobactam), administered IV once every 6 hours. This represents the currently recommended dose in the current ATS/IDSA treatment guidelines [3] for nosocomial pneumonia. The chosen dose is routinely administered to subjects in clinical practice for this indication.

4.2.2.3 Duration of Therapy

IV study therapy will be administered for a minimum of 7 days up to a maximum of 14 days. Of note, subjects with evidence of concurrent bacteremia or with *P. aeruginosa* infection should receive 14 days of treatment. The duration of therapy is consistent with practice guidelines and data from clinical trials.

Guidelines published by ATS/IDSA recommend treatment of HABP/VABP for as few as 7 days, provided that the patient has a good clinical response and the infection is not caused by *P. aeruginosa* [3]. If deemed clinically appropriate, subjects may receive up to 14 days of treatment for non-*Pseudomonas* infections. In the setting of *Pseudomonas* infection, a treatment duration of 14 days is predicated on data from several studies and meta-analyses which show a lesser incidence of persistence or recurrence with ~2 weeks vs. ~1 week of therapy [16] [17]. Lower responses and higher mortality were also recently seen following treatment with a shorter course of therapy (1 week of doripenem) vs. a longer course of therapy (10 days of IMI) in *P. aeruginosa* VABP cases [6]. A treatment duration of 14 days for concurrent bacteremia is supported by current IDSA guidelines on the treatment of bloodstream infections and by standard clinical practice.

Preclinical toxicology data for REL support administration of study therapy for up to 14 days in this study. Refer to the MK-7655 IB for more detailed information on preclinical toxicity studies REL.

4.2.2.4 Rationale for the Use of Comparator

PIP/TAZ is one of several empiric regimens for HABP and VABP recommended by the IDSA and ATS in their current treatment guidelines. It is approved for use in nosocomial pneumonia in the United States, EU, and other countries. Significant clinical experience, from both clinical trials and post-marketing data, exists for the use of PIP/TAZ in the treatment of HABP and VABP. It is the optimal choice for use as a comparator for IMI/REL as both regimens are routinely administered at an every 6-hour interval.

4.2.3 Rationale for Endpoints

4.2.3.1 Efficacy Endpoints

The primary efficacy endpoint in this study is all-cause mortality at Day 28 post-randomization in the MITT population (see Section 8.4.1). Given the severity of illness in hospitalized subjects with HABP/VABP whom routinely require in-hospital treatment with IV antibiotics, evaluation of mortality is appropriate. A one-month mortality endpoint has commonly been used for evaluation of efficacy of antibacterial therapy against HABP/VABP infections [6] [15] [18]. Based on the results of recently conducted trials, approximately 15% of subjects will die even though they receive antibacterial drug therapy for HABP/VABP [14] [12] [19] [15]. The goal of this trial is to demonstrate that IMI/REL is non-inferior to piperacillin/tazobactam (PIP/TAZ) in subjects with HABP/VABP, as measured by the incidence of all-cause mortality through Day 28 post-randomization. If non-inferiority is established, then superiority of IMI/REL compared to PIP/TAZ will be evaluated.

The key secondary measurement for efficacy in this study is:

- Clinical response for each subject evaluated at the early follow-up (EFU) visit, which will be performed 7 to 14 days after the end of therapy (EOT) visit. Assessments will be made in the MITT population. The evaluation of clinical response at this time point will provide valuable data to fully characterize the response profile of the test product relative to the comparator.

There are several other secondary measurements of efficacy in this study:

- All-cause mortality will also be evaluated through Day 28 post-randomization in the mMITT population. This mMITT population for this endpoint will add supportive information to the primary efficacy endpoint in the MITT population.
- All-cause mortality will also be evaluated at the EFU visit in the MITT population and the mMITT population.
- All-cause mortality will also be evaluated through Day 28 post-randomization based on pneumonia type (non-ventilated HABP, ventilated HABP, or VABP) in the MITT population and the mMITT population.
- Clinical response for each subject will also be evaluated at the Day 3, Day 6, and Day 10 on-therapy visits (OTX1, OXT2, and OTX3 [if applicable]), at the EOT visit, and at Day 28 post-randomization in both the MITT and CE populations. In addition, clinical response will be evaluated at the EFU visit in the CE population alone.
- Microbiological response for each subject will also be evaluated at the EOT and EFU visits in both the mMITT and ME populations.

Other exploratory efficacy endpoints in this study include post-randomization hospital LOS, LOS in the ICU (for ventilated HABP and VABP subjects), and number of days spent on mechanical ventilation (for ventilated HABP and VABP subjects). These additional endpoints will provide further information on infection-associated health outcomes important in estimating cost effectiveness of IMI/REL vs PIP/TAZ. Another exploratory endpoint is

the pharmacokinetic evaluation of study therapy (imipenem, REL, and cilastatin). PK/PD evaluations will be incorporated into IMI/REL population PK models to evaluate intrinsic and extrinsic factors that may impact the relationship of PK to PD.

4.2.3.1.1 Definition of Efficacy Endpoints

Refer to Table 1 for a summary favorable response by efficacy endpoint and analysis population.

Table 1 Summary of Efficacy Endpoints and Components of a Favorable Response

Objective	Endpoint	Timing	Favorable Response	Analysis Population	References (Section/Table)
Primary Endpoint	All-Cause Mortality	Day 28	Survival	MITT	Section 4.2.3.1.1.1
Key Secondary	Clinical Response	EFU	• Sustained cure<br>• Cure	MITT	Section 4.2.3.1.1.2<br>Table 4
Other Secondary	All-Cause Mortality	Day 28	Survival	mMITT	Section 4.2.3.1.1.1
	All-Cause Mortality	EFU	Survival	MITT and mMITT	Section 4.2.3.1.1.1
	All-Cause Mortality (non-ventilated HABP, ventilated HABP, or VABP)*	Day 28	Survival	MITT and mMITT	Section 4.2.3.1.1.1
	Clinical response	OTX1, OTX2, OTX3	• Improved	MITT and CE	Section 4.2.3.1.1.2<br>Table 2
		EOT	• Cure<br>• Improved		Section 4.2.3.1.1.2<br>Table 3
		EFU, Day 28	• Sustained cure<br>• Cure		Section 4.2.3.1.1.2<br>Table 4
	Microbiological response	EOT, EFU	Eradication	mMITT and ME	Section 4.2.3.1.1.3<br>Table 5 and Table 6
Exploratory	All-cause mortality or disease-related complications (e.g., empyema, onset of acute respiratory distress syndrome, bacteremia)	Day 28	Similar survival and disease-related complications compared to PIP/TAZ	MITT	Section 4.2.3.1.1.1
	Number of days spent in the hospital post-randomization	NA	NA	MITT and mMITT	Section 7.1.3.6.5
	Number of days spent on mechanical ventilation post-randomization	NA	NA	MITT and mMITT	Section 7.1.3.6.5
	Number of days spent in ICU post-randomization	NA	NA	MITT and mMITT	Section 7.1.3.6.5

Day 28 = Day 28 post-randomization; EFU = early follow-up visit; EOT = end of therapy visit; MITT = modified intention to treat population; mMITT = microbiological modified intention to treat population; CE = clinically evaluable population; NA= not applicable
*Sub-group analysis of all-cause mortality will be conducted for each of the sub-groups.

4.2.3.1.1.1 Survival/All-cause Mortality

Survival status (i.e., whether the subject is alive or dead) through Day 28 post-randomization and EFU visits will be evaluated for all subjects in support of the primary and key secondary objectives, respectively.

4.2.3.1.1.2 Clinical Response

Clinical response will be assessed for all subjects based on evaluation by the investigator at the OTX1, OTX2, OTX3 (if applicable), EOT, EFU, and Day 28 post-randomization visits. Based on comparison to baseline clinical signs and symptoms of the subject's infection, the investigator will determine the clinical response rating at each visit as described in Table 2 (for OTX1, OTX2 and OTX3 visits, If applicable), Table 3 (for EOT visit), and Table 4 (for EFU and Day 28 post-randomization visits).

The clinical response rating determined by the investigator at each visit will be categorized as "favorable" or "unfavorable". Details regarding determination of the category of clinical response ("favorable" or "unfavorable") in support of relevant study endpoints are provided in the tables describing clinical response and in the full protocol in Section 8.2.

Table 2 Definitions of the Clinical Response Rating at the OTX Visits (Day 3, Day 6 and Day 10 of IV Study Therapy)

Clinical Response[a]	Response Definition
Improved	The majority of pre-therapy signs and symptoms[b] of the index infection have improved or resolved (or returned to "pre-infection status")
Persistence	Little apparent response to IV study therapy in pre-study signs and symptoms[b] of the index infection(s): persistence of the majority of or all pre-therapy signs and symptoms.
Progression	Worsening of response while on IV study therapy in pre-study signs and symptoms[b] of the index infection(s): progression of the majority of or all pre-therapy signs and symptoms.
Indeterminate	Study data are not available for evaluation of clinical response for any reasons at the OTX1, OTX2 or OTX3 visit, including:<br>a) Complication related to underlying medical condition; **OR**<br>b) Subject was withdrawn for any reason before sufficient data had been obtained to permit evaluation for any reason; **OR**<br>c) Extenuating circumstances (e.g., a major protocol violation) preclude classification as "improved," "persistence," or "progression;" **OR**<br>d) Death occurred during the study period and the index infection was clearly noncontributory.

a. A favorable clinical response at OTX1, OTX2, or OTX3 requires an assessment of "improved".
b. Refer to Inclusion #3 and Table 10 for a description of relevant clinical signs and symptoms.

Table 3 Definitions of the Clinical Response Rating at the EOT Visit

**Clinical Response**[a]	**Response Definition**
Cure	All pre-therapy signs and symptoms[b] of the index infection have resolved (or returned to "pre-infection status") **AND** no additional antibiotic therapy is required for the index infection.
Improved	The majority of pre-therapy signs and symptoms[b] of the index infection have improved or resolved (or returned to "pre-infection status") **AND** no additional antibiotic therapy is required.
Failure	No apparent response to IV study therapy in pre-study signs and symptoms[b] of the index infection: persistence or progression of the majority of or all pre-therapy signs and symptoms.
Indeterminate	Study data are not available for evaluation of clinical response for any reasons at the EOT visit, including:<br>a) Complication related to underlying medical condition; **OR**<br>b) Subject was withdrawn for any reason before sufficient data had been obtained to permit evaluation for any reason; **OR**<br>c) Extenuating circumstances (e.g., a protocol violation) preclude classification as "cure," "improved," or "failure;" **OR**<br>d) Death occurred during the study period and the index infection was clearly noncontributory.

a. A favorable clinical response at EOT requires an assessment of "cure" or "improved".
b Refer to Inclusion #3 and Table 10 for a description of relevant clinical signs and symptoms.

Table 4 Definitions of the Clinical Response Rating at the EFU Visit and Day 28 Post-Randomization Visit

**Clinical Response**[a,]	**Response Definition**
Sustained Cure	All pre-therapy signs and symptoms[b] of the index infection have resolved (or returned to "pre-infection status") with no evidence of resurgence **AND** no additional antibiotic therapy was required for the index infection.
Cure	All pre-therapy signs and symptoms[b] of the index infection have resolved (or returned to "pre-infection status") **AND** no additional antibiotic therapy is required for the index infection.
Failure	No apparent or insufficient response to IV study therapy in pre-study signs and symptoms of the index infection: persistence, progression, or improvement (without full resolution) of all pre-therapy signs and symptoms[b]
Relapse	Subjects with a favorable clinical response (cure or improved) at the EOT visit have worsening signs and symptoms[b] of the index infection by the EFU or Day 28 post-randomization visit.
Indeterminate	Study data are not available for evaluation of efficacy for any reasons, including:<br>a) Complication related to underlying medical condition; **OR**<br>b) Subject was withdrawn for any reason before sufficient data had been obtained to permit evaluation of clinical response; **OR**<br>c) Extenuating circumstances (e.g., a major protocol violation) preclude classification as "sustained cure," "failure," or "relapse;" **OR**<br>d) Death occurred during the study period and the index infection was clearly noncontributory.

[a] A favorable clinical response at EFU or Day 28 post-randomization requires an assessment of “cure” or “sustained cure”. To be considered “sustained cure”, the clinical response for the prior visit (EOT or EFU) must have been considered “cure”.
[b] Refer to Inclusion #3 and Table 10 for a description of clinical signs and symptoms.

4.2.3.1.1.3 Microbiological Response

In addition to clinical response assessments, subjects will be evaluated for microbiological response. Microbiological response will be evaluated separately for each lower respiratory tract pathogen isolated in the baseline culture (i.e., by-pathogen). The by-pathogen response rating determined by the investigator will be assessed based on local laboratory results.

A by-pathogen microbiological response rating will be determined by the investigator at EOT, and EFU visits based on the local laboratory results of lower respiratory tract cultures collected for subjects at each of these visit, when available, relative to the pathogen(s) isolated at baseline/admission as described in Table 5 (for EOT visit) and Table 6 (for EFU visit). Lower respiratory tract cultures would preferentially include samples collected from a tracheostomy or endotracheal aspirates, or bronchoscopy specimens. Expectorated or induced sputum samples are also accepted provided the sample does not represent oropharyngeal contamination (i.e., sample contains fewer than 10 squamous epithelial cells and greater than 25 neutrophils on low power microscopy review of the Gram stain).

Antibiotic susceptibility results from the Central Microbiology Lab, as well as microbiological response, will be summarized by pathogen. The by-pathogen microbiological response rating determined by the investigator at each EOT and EFU visits will be utilized to categorize.

The overall microbiological response (i.e., overall microbiological response for the subject based on the response of all pathogens present in the baseline lower respiratory tract culture) will be assessed as "favorable" or "unfavorable." For subjects from whom only one pathogen is isolated in the baseline lower respiratory tract culture, the overall microbiological response assessment will be based on the microbiological response rating for that pathogen. For randomized subjects from whom more than one baseline pathogen is isolated in the baseline lower respiratory tract culture, the overall microbiological response outcome will be based on microbiological culture results for all pathogens (i.e., a "favorable" overall microbiological response requires eradication of all baseline pathogens).

Details regarding determination of the category of overall microbiological response outcome ("favorable" or "unfavorable") in support of relevant study endpoints are provided in the tables describing microbiological response and in the full protocol in Section 8.4.1.

Table 5 Definitions of the By-Pathogen Microbiological Response Rating at the EOT Visit

**Microbiological Response**[a,b,c]	**Response Definition**
Eradication	A lower respiratory tract culture taken at the EOT visit[d] shows eradication of the pathogen found at study entry.
Persistence[e]	A lower respiratory tract culture taken at the EOT visit[d] grows the pathogen found at study entry.
Superinfection	An infection-site culture grows a pathogen other than a baseline pathogen during the course of IV study therapy **OR** emergence during IV study therapy of a new pathogen at a distant sterile site along with worsening signs and symptoms of infection.
Indeterminate	a) Follow-up culture is not available at the EOT visit[d] due to subject death or withdrawal from study; **OR**<br>b) Available microbiological data are incomplete; **OR**<br>c) Extenuating circumstances (e.g., a major protocol violation) preclude microbiological assessment; **OR**<br>d) Any other circumstance which makes it impossible to define the microbiological response<br>e) No specimen taken because subject is deemed clinically cured or improved

a. A microbiological response rating must be completed separately for each pathogen isolated at baseline. If a new/emergent pathogen is identified at this visit which was not identified at baseline, the microbiological response rating should be recorded as "superinfection" for any new/emergent pathogen isolated after initiation of IV study therapy.
b. The response of "No specimen taken because subject is deemed clinically cured or improved" will be considered to be "presumed eradication".
c. A favorable by-pathogen microbiological response at EOT requires "eradication" or "presumed eradication" (i.e., Indeterminate - No specimen taken because subject is deemed clinically cured or improved) of the baseline pathogen.
d. If a culture is not available at EOT, an assessment at this visit can be made from the last available culture which was collected after at least 72 hours of IV study therapy.
e. If a subject is discontinued from IV study therapy due to clinical failure (i.e., unfavorable clinical response), but persistence of the admission pathogen is not confirmed by culture results or no culture is obtained at the time of clinical failure, the admission pathogen will be presumed to have persisted.

Table 6 Definitions of the By-Pathogen Microbiological Response at the EFU Visit

Microbiological Response[a,b,c]	Response Definition
Eradication	A lower respiratory tract culture taken at the EFU[d] visit shows eradication of the pathogen found at study entry.
Persistence	A lower respiratory tract culture taken at the EFU visit grows the pathogen found at study entry.
New Infection	A pathogen, other than an original microorganism found at baseline is present in the lower respiratory tract specimen any time after IV study therapy is finished; **OR**<br>A pathogen is isolated from a distant sterile site <u>after</u> IV study therapy has been completed.
Recurrence	A lower respiratory tract specimen grows the baseline pathogen taken any time after documented eradication.
Indeterminate	a) Follow-up culture is not available at the EFU visit due to subject death or withdrawal from study; **<u>OR</u>**<br>b) Available microbiological data are incomplete; **<u>OR</u>**<br>c) Extenuating circumstances (e.g., a major protocol violation) preclude microbiological assessment; **<u>OR</u>**<br>d) Any other circumstance which makes it impossible to define the microbiological response<br>e) No specimen taken because subject is deemed clinically cured or improved

a. A microbiological response rating must be completed separately for each pathogen isolated at baseline. If a new/emergent pathogen is identified at this visit which was not identified at baseline, the microbiological response rating should be recorded as "new infection" for any new/emergent pathogen isolated after initiation of IV study therapy.
b. The response of "No specimen taken because subject is deemed clinically cured or improved" will be considered to be "presumed eradication".
c. A favorable by-pathogen microbiological response at EFU visit requires "eradication" or "presumed eradication" (i.e., Indeterminate - No specimen taken because subject is deemed clinically cured or improved) of the baseline pathogen.
d. If a culture is not available at EFU, an assessment at this visit can be made based on the culture collected at EOT as long as it was collected at least 24 hours after the end of IV therapy and before the EFU visit and provided the subject had fully resolved clinical symptoms/signs of the index infection at the EFU visit.

4.2.3.2 Safety Endpoints

In support of the secondary objective to evaluate the safety and tolerability profile of IMI/REL, the safety and tolerability of IMI/REL (as well as the safety of the comparator, PIP/TAZ) will be assessed by clinical evaluation of adverse events and inspection of other study parameters including vital signs, physical examinations, and standard laboratory safety tests at time points specified in the Trial Flow Chart. Adverse events are graded and recorded according to Section 7.2. Subjects may be asked to return for unscheduled visits in order to perform additional safety monitoring.

4.2.3.3 Pharmacokinetic Endpoints

At the time points specified in the Trial Flow Chart (Section 6.0), whole blood samples will be collected for determination of plasma concentrations of REL, imipenem, and cilastatin. These samples will support further evaluation of the pharmacokinetic profiles of these drugs by confirming that subjects achieve expected exposures, and will also aid in further assessment of the clinical relationship between IMI and REL plasma concentrations and efficacy.

4.2.3.4 Planned Exploratory Biomarker Research

Planned Genetic Analysis

Understanding genetic determinants of drug response is an important endeavor during medical research. This research will evaluate whether genetic variation within a clinical trial population correlates with response to the treatment(s) under evaluation. If genetic variation is found to predict efficacy or adverse events, the data might inform optimal use of therapies in the patient population. This research contributes to understanding genetic determinants of efficacy and safety associated with the treatments in this study.

4.2.3.5 Future Biomedical Research

The Sponsor will conduct Future Biomedical Research on DNA specimens collected for future biomedical research during this clinical trial.

Such research is for biomarker testing to address emergent questions not described elsewhere in the protocol (as part of the main trial) and will only be conducted on specimens from appropriately consented subjects. The objective of collecting specimens for Future Biomedical Research is to explore and identify biomarkers that inform the scientific understanding of diseases and/or their therapeutic treatments. For instance, exploratory pharmacogenetic (PGt) studies may be performed if significant Pharmacokinetic/Pharmacodynamic (PK/PD) relationships are observed or adverse events are identified. Genomic markers of disease may also be investigated. Such retrospective pharmacogenetic studies will be conducted with appropriate biostatistical design and analysis and compared to PK/PD results or clinical outcomes. Any significant PGt relationships to outcome would require validation in future clinical trials. The overarching goal is to use such information to develop safer, more effective drugs/vaccines, and/or to ensure that subjects receive the correct dose of the correct drug/vaccine at the correct time. The details of this Future Biomedical Research sub-trial are presented in Section 12.2 - Collection and Management of Specimens for Future Biomedical Research. Additional informational material for institutional review boards/ethics committees (IRBs/ERCs) and investigational site staff is provided in Section 12.3.

4.3 Benefit/Risk

Subjects enrolled in this trial are hospitalized patients with infections requiring treatment with IV therapy. If randomized to receive IMI/REL, subjects will receive treatment, in part, with an agent, IMI, recommended and commonly used for the treatment of HABP/VABP, and in addition can potentially benefit from treatment with the investigational agent REL.

The combination, IMI/REL, is specifically targeted for treatment of gram-negative imipenem-resistant infections. Subjects randomized to the comparator arm will also receive a treatment regimen expected to be efficacious against HABP/VABP (see Section 4.2.2 and 4.2.2.1).

Although potentially more frequent than standard of care, the study procedures described in Section 6.0 (Trial Flow Chart) are generally typical procedures performed for this hospitalized patient population. Additional burden may be incurred due to visits following release from the hospital. However, the procedures performed at these visits are generally not likely to lead to significant harm (e.g., blood draws, urine collection, physical exam, vital signs). These procedures are necessary to support a robust evaluation of the safety and efficacy of the investigational drug.

Additional details regarding specific benefits and risks for subjects participating in this clinical trial may be found in the accompanying MK-7655A Investigators Brochure (IB) and Informed Consent documents.

5.0 METHODOLOGY

5.1 Entry Criteria

5.1.1 Diagnosis/Condition for Entry into the Trial

Male and female subjects who are at least 18 years of age and have been diagnosed with either hospital-acquired (HABP) or ventilator-associated bacterial pneumonia (VABP) will be enrolled in this trial.

5.1.2 Subject Inclusion Criteria

In order to be eligible for participation in this trial, the subject must:

1. be ≥ 18 years of age on the day of signing informed consent.

   NOTE: Adults are the intended study population for this protocol. Subjects under the age of legal consent per a specific country's regulation should be excluded from participation in this study.

2. require treatment with IV antibiotic therapy for hospital-acquired bacterial pneumonia (HABP) or ventilator-associated bacterial pneumonia (VABP).

   NOTE: HABP is an acute infection of the pulmonary parenchyma that is associated with clinical signs and symptoms accompanied by presence of new or progressive infiltrate on chest radiograph *occurring in a subject after being hospitalized for more than 48 hours or within 7 days after discharge from a hospital stay of at least 48 hours duration (includes patients institutionalized in a skilled nursing or other long-term care facility)*. Of note, such subjects may or may not require mechanical ventilation (ventilated HABP and non-ventilated HABP). VABP is an acute infection of the pulmonary parenchyma that is associated with clinical signs and symptoms accompanied by presence of new or progressive infiltrate on chest radiograph

occurring in a subject already receiving mechanical ventilation via an endotracheal tube for a minimum of 48 hours.

3. fulfill the clinical and radiographic criteria described below, with onset of criteria occurring after more than 48 hours of hospitalization or within 7 days after discharge from a hospital (for HABP) or at least 48 hours after mechanical ventilation (for VABP):

(a) **Subject has at least one of the following clinical features:**

- New onset or worsening pulmonary symptoms or signs, such as cough, dyspnea, tachypnea (e.g., respiratory rate greater than 25 breaths per minute), expectorated sputum production, or requirement for mechanical ventilation
- Hypoxemia [e.g., a partial pressure of oxygen less than 60 millimeters of mercury while the subject is breathing room air, as determined by arterial blood gas (ABG) or worsening of the ratio of the partial pressure of oxygen to the fraction of inspired oxygen (PaO_2/FiO_2)]
- Need for acute changes in the ventilator support system to enhance oxygenation, as determined by worsening oxygenation (ABG or PaO_2/FiO_2) or needed changes in the amount of positive end-expiratory pressure
- New onset of suctioned respiratory secretions

AND

(b) Subject has at least one of the following signs:

- Documented fever (e.g., body temperature ≥ 38 degrees Celsius)
- Hypothermia (e.g., core body temperature ≤ 35 degrees Celsius)
- Total peripheral white blood cell (WBC) count ≥ 10,000 cells/cubic millimeter (mm^3)
- Leukopenia with total WBC ≤4,500 cells/mm^3
- Greater than 15 percent immature neutrophils (bands) noted on peripheral blood smear

AND

(c) Subject has a chest radiograph showing the presence of a new or progressive infiltrate(s) suggestive of bacterial pneumonia

4. have an adequate baseline (at or within 48 hours of screening) lower respiratory tract specimen obtained for Gram stain and culture.

NOTE: Microscopic examination of Gram stained smears must be performed prior to randomization to ensure the adequacy of the specimen. The low-power microscopic view of the Gram stain can be used to ascertain the quality of the respiratory specimen which helps to ensure that the respiratory specimen sent for culture does not represent oropharyngeal contamination. For specimens obtained by direct sampling of the lower respiratory tract [the ONLY direct sampling techniques accepted in the study are the following 3 methods: i.e., sampling via bronchoalveolar lavage (BAL),

mini BAL or protected brush specimen (PBS)], no predefined requirements are required to ascertain the quality of the respiratory specimen. However, for specimens not obtained by direct sampling of the lower respiratory tract, such as those obtained by expectorated sputum, an adequate lower respiratory tract specimen is defined as having fewer than 10 squamous epithelial cells and greater than 25 neutrophils. In addition, a high-power microscopic view of the Gram stain can be used to characterize the general type of bacteria causing the pneumonia. Specimens should be processed for culture according to recognized methods.

5. have an infection known or thought to be, in the opinion of the investigator, caused by microorganisms susceptible to the IV study therapy.
6. agree to allow any bacterial isolates obtained from protocol-required specimens related to the current infection to be provided to the Central Microbiology Reference Laboratory for study-related microbiological testing, long-term storage, and other future testing.
7. understand (or have a legal representative that understands) the study procedures, alternative treatments available, and risks involved with the study, and voluntarily agree to participate by giving written informed consent for the trial. The subject may also provide consent for Future Biomedical Research. However, the subject may participate in the main trial without participating in Future Biomedical Research.
8. meet one of the following categories:

a) The subject is a male who is not of reproductive potential. A male subject who is not of reproductive potential is defined as a male whom is not sexually active or has undergone a successful vasectomy. A successful vasectomy is defined as: (1) microscopic documentation of azoospermia, or (2) a vasectomy more than 2 years ago with no resultant pregnancy despite sexual activity post-vasectomy.

NOTE: A male subject who is not of reproductive potential is eligible without requiring the use of contraception.

OR

b) the subject is a female who is not of reproductive potential. Female subjects meeting at least one of the following criteria are considered not of reproductive potential (1) women age $\geq$ 50 years AND no menses for $\geq$12 months OR FSH >40 mIU/mL; (2) women age $\geq$ 45 years AND no menses for $\geq$18 months OR FSH >40 mIU/mL; (3) women with documented congenital/acquired condition that prevents childbearing; OR (4) women with documented total abdominal hysterectomy (TAH) and/or bilateral salpingo-oophorectomy (BSO).

NOTE: A female subject who is not of reproductive potential is eligible without requiring the use of contraception.

OR

c) the subject is a female or a male who is of reproductive potential and agrees to avoid becoming pregnant or impregnating a partner from the time of consent through completion of the study by complying with one of the following: (1) practice abstinence† from heterosexual activity OR (2) use (or have their partner use)

acceptable contraception during heterosexual activity. Acceptable methods of contraception are‡:

Single method (one of the following is acceptable):

- non-hormonal intrauterine device (IUD)
- vasectomy of a female subject's male partner
- male condom or female condom (cannot be used together)
- diaphragm with spermicide (cannot be used in conjunction with cervical cap/spermicide)
- cervical cap with spermicide (nulliparous women only)
- contraceptive sponge (nulliparous women only)

†True abstinence (relative to heterosexual activity) starting from the time of consent through completion of the study: abstinence is in line with the preferred and usual lifestyle of the subject and if considered acceptable by local regulatory agencies and ERCs/IRBs. Periodic abstinence (e.g., abstinence only on certain calendar days, abstinence only during ovulation period, use of symptom-thermal method, is of post-ovulation methods) and withdrawal are not acceptable methods of contraception.

‡If a contraceptive method listed above is restricted by local regulations/guidelines, then it does not qualify as an acceptable method of contraception for subjects participating at sites in this country/region.

For Early Consenting Option ONLY

9. is anticipated to require extended intubation based on the opinion of the investigator, which puts the subject at risk of developing VABP. Subject must have been intubated for at least 48 hours prior to developing signs and symptoms of pneumonia in order to qualify for assessment of disease eligibility at ≤48 hours prior to randomization.

5.1.3 Subject Exclusion Criteria

The subject must be excluded from participating in the trial if the subject:

1. has a baseline lower respiratory tract specimen Gram stain that shows the presence of gram-positive cocci **only**.

   NOTE: For example, the following Gram stain patterns are acceptable:

   - only gram-negative organisms;
   - only gram-positive rods;
   - a mixture of gram-positive and gram-negative organisms;
   - a mixture of gram-positive cocci and gram-positive rods; or
   - no organisms seen on Gram stain.

2. has confirmed or suspected community-acquired bacterial pneumonia (CABP).

3. has confirmed or suspected pneumonia of viral, fungal, or parasitic etiology.
4. has HABP/VABP caused by an obstructive process, including lung cancer (or other malignancy metastatic to the lungs resulting in pulmonary obstruction) or other known obstruction.
5. has a carcinoid tumor or carcinoid syndrome.
6. has active immunosuppression, defined as either receiving immunosuppressive medications or having a medical condition associated with immunodeficiency.

   NOTE: Short-term treatment with systemic (IV or oral) steroids of ≤1 week duration (e.g., treatment for an acute asthma exacerbation or acute skin condition) is allowed. Topical steroids for the treatment of skin conditions are also allowed.
7. is expected to survive < 72 hours.
8. has a concurrent condition or infection that, in the investigator's judgment, would preclude evaluation of therapeutic response (e.g. active tuberculosis, cystic fibrosis, granulomatous disease, a disseminated fungal infection, invasive fungal pulmonary infection or endocarditis).

   NOTE: Subjects in whom fungal pathogens are isolated are eligible for participation provided that treatment of the fungus is not planned.
9. has received effective antibacterial drug therapy for the index infection of HABP/VABP for a continuous duration of more than 24 hours during the previous 72 hours.

   NOTE: Subjects are only allowed to have received such antibacterial agents for up to 24 hours prior to study treatment.

   Exceptions:

   - Subjects who have failed prior antibiotic therapy for the current episode of HABP/VABP (i.e., have persistent/worsening signs and/or symptoms of HABP/VABP at screening which are still present despite >48 hours on the prior antibacterial regimen), provided the prior respiratory or blood culture did not grow only *S. aureus* (methicillin-susceptible *S. aureus* [MSSA] or methicillin-resistant S. aureus [MRSA]).
   - Prior therapy with a non-absorbed antibiotic therapy used for gut decontamination (e.g., low dose erythromycin or polymyxin) or to eradicate *C. difficile* (oral vancomycin or fidaxomicin).
10. has a history of serious allergy, hypersensitivity (e.g., anaphylaxis), or any serious reaction to any of the following:
    - any penicillin (including PIP/TAZ, carbapenems, cephalosporins, or other β-lactam agents)

- β-lactamase inhibitors (e.g., tazobactam, sulbactam, clavulanic acid, avibactam)

NOTE: Subjects with a history of mild rash to penicillins or other β-lactams may be enrolled and closely monitored.

11. is a female who is pregnant or is expecting to conceive (or is a male partner of a female who is expecting to conceive), is breastfeeding, or plans to breastfeed prior to completion of the study.

12. has a history of a seizure disorder which has required ongoing treatment with anti-convulsive therapy or prior treatment with anti-convulsive therapy within the last 3 years.

    NOTE: Among patients with no active or prior history of seizure disorder, it is acceptable to use non-valproate anticonvulsants for mood disorders, behavior disturbance, or to prevent seizures following stroke, neurosurgery, or head trauma (See Exclusion #13).

13. is anticipated to be treated with any of the following medications during the course of study therapy:

    - valproic acid or divalproex sodium (or has used valproic acid or divalproex sodium in the 2 weeks prior to screening)
    - serotonin re-uptake inhibitors, tricyclic antidepressants, or serotonin 5-HT1 receptor agonists (triptans)
    - monoamine oxidase inhibitors (MAOIs) (or has used MAOIs during the 2 weeks prior to screening)
    - meperidine
    - buspirone
    - concomitant systemic (IV or oral) antibacterial agents in addition to those designated in the study treatment groups

      NOTE: As the study will enroll subjects without confirmed microbiological (culture) evidence of the HABP/VABP pathogen on a lower respiratory tract specimen at study entry, the use of initial empiric treatment with open-label IV linezolid for methicillin-resistant *S. aureus* (MRSA) infection is required. The recommended treatment duration for a confirmed MRSA infection is a minimum of 7 days, with a minimum of 14 days for MRSA bacteremia. Empiric linezolid treatment will be stopped if the final culture results from the baseline lower respiratory tract sample do not demonstrate the presence of MRSA.

    - Concomitant systemic (IV or oral) antifungal or antiviral therapy for the index infection of HABP/VABP.

14. has an estimated or actual creatinine clearance of < 15 mL/min at screening, based on the findings of local laboratory values. Creatinine clearance in mL/min may be calculated from serum creatinine concentration by the Cockcroft-Gault (C-G) equation:

    $$\text{Creatinine clearance (Males)} = \frac{\text{(weight in kg) X (140 minus age)}}{\text{(72) X (creatinine in mg/dL)}}$$

    Creatinine clearance (Females) = 0.85 X the value obtained using the formula above

15. is currently undergoing hemodialysis or peritoneal dialysis.
16. has a history or current evidence of any condition, therapy, laboratory abnormality, or other circumstance that, in the opinion of the investigator, might confound the results of the study, interfere with the subject's participation for the full duration of the study, or pose additional risk in administering the study drugs to the subject.
17. is currently participating in, or has participated in, any other clinical study involving the administration of investigational or experimental medication (not licensed by regulatory agencies) at the time of the presentation or during the previous 30 days prior to screening or is anticipated to participate in such a clinical study during the course of this trial.
18. has previously participated in this study at any time.
19. is or has an immediate family member (e.g., spouse, parent/legal guardian, sibling or child) who is investigational site or sponsor staff directly involved with this trial.

5.2 Trial Treatment(s)

The blinded IV study therapies to be used in this trial are outlined below in Table 7.

Table 7 Trial Treatment

Drug[a]	Dose/Potency[b]	Dose Frequency	Route of Administration	Regimen/ Treatment Period[c]	Use
**Treatment Group 1 (N=268)**					
Imipenem/ relebactam (IMI/REL)	Imipenem/relebactam 500 mg/250 mg	Every 6 hours	IV	7 to 14 days	Experimental
**Treatment Group 2 (N=268)**					
Piperacillin/ tazobactam (PIP/TAZ)	Piperacillin/ tazobactam 4000 mg/500 mg	Every 6 hours	IV	7 to 14 days	Active - Comparator

[a] IMI/ REL and PIP/TAZ are each provided as a fixed-dose combination (FDC) in a single vial. IMI/REL also includes 500 mg of cilastatin within the FDC.
[b] Adjustments to dosage of IMI/REL or PIP/TAZ are required for subjects with renal insufficiency (Please reference Table 8 and Table 9 in Section 5.2.1.1).
[c] IV study therapy should be administered for a minimum of 168 hours (7 full days). Seven full days of therapy corresponds to 28 doses of IMI/ REL (Treatment Group 1) and PIP/TAZ (Treatment Group 2) for every 6 hours (q6h). The total duration of IV study therapy should not exceed 14 days.

The first dose of prescribed study therapy should be administered at the Day 1 visit. The Unblinded Study Pharmacist (or qualified designee) will contact the IVRS/IWRS for assignment of the study therapy to be administered. Sites should not call IVRS/IWRS for drug administration until the subject has met all entry criteria for the study and ready to be randomized. Randomized subjects in each treatment group will receive a minimum of 7 days to up to a maximum of 14 days of intravenous (IV) study therapy. Of note, subjects with evidence of concurrent bacteremia or with *P. aeruginosa* involvement should receive 14 days of IV study therapy.

Detailed dosing guidelines, including dose selection and timing of dose administration, are outlined in Section 5.2.1 and Section 5.2.2, respectively.

Co-administration of Open-label Therapy with Linezolid:

As the study may enroll subjects without confirmed microbiological (culture) evidence of the HABP/VABP pathogen on a lower respiratory tract specimen at study entry, the use of initial empiric treatment with IV linezolid for methicillin-resistant *S. aureus* (MRSA) infection is required. The recommended treatment duration for a confirmed MRSA infection is a minimum of 7 days, with a minimum of 14 days for MRSA bacteremia. Empiric linezolid treatment will be stopped if the final culture results from the baseline lower respiratory tract sample do not demonstrate the presence of MRSA. Linezolid will be given open-label in both treatment groups. Linezolid will not be provided by the Sponsor.

The investigator shall take responsibility for and shall take all steps to maintain appropriate records and ensure appropriate supply, storage, handling, distribution and usage of trial treatments in accordance with the protocol and any applicable laws and regulations.

5.2.1 Dose Selection/Modification

5.2.1.1 Dose Selection (Preparation)

5.2.1.1.1 IMI/REL

The chosen dose for IMI/REL in subjects with normal renal function is IMI/REL 500mg/250 mg administered IV as an FDC once every 6 hours. **For subjects with renal insufficiency or whose creatinine clearance changes during treatment with study therapy, the dose must be adjusted by the Unblinded Study Pharmacist (or qualified designee) based upon the degree of renal function impairment, as determined by the estimated or actual creatinine clearance**. Subjects should be carefully monitored for renal function during IV treatment. Dose adjustments are included in Table 8.

Table 8 Administration Dosage of IMI/REL According to Renal Function

Creatinine Clearance (mL/min)	IMI/REL[a]
≥ 90	500/250 mg q6h
< 90 to ≥ 60	400/200 mg q6h
< 60 to ≥ 30	300/150 mg q6h
< 30 to ≥ 15	200/100 mg q6h

[a] IMI/REL is provided as a single vial in a fixed-dose combination; therefore, the dose for each component will be adjusted equally during preparation. For example, a subject who has a creatinine clearance of 50 mL/min should receive a 300/150 mg q6h dose of IMI/REL according to the table.

Creatinine clearance in mL/min may be calculated from serum creatinine concentration by the following equation:

$$\text{Creatinine clearance (Males)} = \frac{\text{(weight in kg) X (140 minus age)}}{\text{(72) X (creatinine in mg/dL)}}$$

Creatinine clearance (Females) = 0.85 X the value obtained using the formula above.

5.2.1.1.2 PIP/TAZ

Subjects in Treatment Group 2 will receive piperacillin/tazobactam (PIP/TAZ) as an FDC at a dosage of 4500 mg (4000 mg PIP/500 mg TAZ) every six hours, resulting in a daily total of 18.0 g (16.0 g PIP/2.0 g TAZ). **For subjects with renal insufficiency or whose creatinine clearance changes during treatment with study therapy, the dose must be adjusted by the Unblinded Study Pharmacist (or qualified designee) based upon the degree of renal function impairment, as determined by the estimated or actual creatinine clearance.** Subjects should be carefully monitored for renal function during IV treatment. The recommended daily doses for subjects with renal impairment per the US product label are included in Table 9.

<u>**NOTE**</u>: Assuming there are no market shortages, country requirements for local sourcing of comparator, or any additional sourcing issues, the study is planning on using an EU central source for the comparator throughout the life of the study. According to the EU product label, the recommended dosing for subjects with renal insufficiency (based on creatinine clearance) is based on the adjustment of the frequency of administration of PIP/TAZ from

every 6 hours to every 8 or every 12 hours (depending on creatinine clearance value) while maintaining the dose of PIP/TAZ the same. This would require the need to include placebo-matching infusion of IV normal saline to maintain study blind between treatment groups. The additional fluid overload to these critically ill subjects and the additional resources and operational challenges for healthcare personnel at the site would make the study unfeasible. As a result, dosing of PIP/TAZ in this study should be administered per Table 9 below based on the US product information.

Table 9 Recommended Dosage of PIP/TAZ in Subjects According to Renal Function (Total PIP/TAZ dosage provided first, followed by individual components in parentheses

Creatinine clearance (mL/min)	PIP/TAZ Dose[a] for Nosocomial Pneumonia
> 40	4500 mg (4000 mg PIP/500 mg TAZ) q6h
40 to ≥20	3375 mg (3000 mg PIP/375 mg TAZ) q6h
< 20 to ≥15	2250 mg (2000 mg PIP/250 mg TAZ) q6h

[a] PIP/TAZ is provided as a single vial in a fixed-dose combination; therefore, the dose for each component will be adjusted equally during preparation. For example, a subject who has a creatinine clearance of 30 mL/min should receive a 3375 mg dose (3000 mg PIP/375 mg TAZ) according to the table.

Creatinine clearance in mL/min may be calculated from serum creatinine concentration by the following equation:

$$\text{Creatinine clearance (Males)} = \frac{\text{(weight in kg) X (140 minus age)}}{\text{(72) X (creatinine in mg/dL)}}$$

Creatinine clearance (Females) = 0.85 X the value obtained using the formula above.

5.2.2 Timing of Dose Administration

As shown in Table 8 and Table 9, the dose of IMI/REL and PIP/TAZ may need to be adjusted based on the subject's renal function. The frequency of administration will not change. Each infusion should be administered within 60 minutes of the scheduled dose.

IMI/REL (Treatment Group 1) and PIP/TAZ (Treatment Group 2) should be administered over 30 minutes +/- 10 minutes. The study therapy must NOT be administered simultaneously through the same infusion line/lumen with any other drugs (including IV non-study drugs). If another IV drug is required either prior to or after study drug and only 1 line/lumen is available, an appropriate volume of saline flush must be used between IV infusions.

Additional details for preparation and administration of study drug are provided in a separate Pharmacy Manual.

5.2.3 Trial Blinding/Masking

A double-blind/masking technique will be used. IMI/REL and PIP/TAZ will be dispensed in a blinded fashion by an unblinded pharmacist or qualified trial site personnel. Study therapy supplies will be provided in an open-label fashion to the sites; hence an Unblinded Study Pharmacist (or qualified designee) at the study site will be responsible for preparing the IV study therapy for this study; this individual(s) must not be involved in any safety or efficacy evaluations of the study participants. Due to a visual difference in the appearance of study treatment solutions, the infusion bags will be covered with an opaque sleeve by the Unblinded Study Pharmacist (or qualified designee) to ensure that other study personnel and all subjects remain blinded to clinical material assignments. The intravenous line (through which the infusion is administered) does not require opaque covering since the differences between the clinical materials are not visually distinguishable within the tubing. Once each infusion bag is properly prepared and masked with an opaque sleeve, the study therapy will be administered by qualified trial site personnel. The personnel involved in the administration of the study infusion treatment should be unaware of the treatment group assignments. See Section 7.1.4.2, (Blinding/Unblinding), for a description of the method of unblinding a subject during the trial, should such action be warranted. The subject, the investigator and Sponsor personnel or delegate(s) who are involved in the treatment or clinical evaluation of the subjects are unaware of the group assignments.

As the study may enroll subjects without confirmed microbiological (culture) evidence of the HABP/VABP pathogen on a lower respiratory tract specimen at study entry, subjects will receive treatment with open-label IV linezolid for methicillin-resistant *S. aureus* (MRSA) infection beginning at randomization.

5.3 Randomization or Treatment Allocation

Randomization will occur centrally using an interactive voice response system / integrated web response system (IVRS/IWRS). There are 2 randomized treatment arms. Subjects will be assigned randomly in a 1:1 ratio to IMI/REL (Treatment Group 1; N=268) or PIP/TAZ (Treatment Group 2; N=268).

5.4 Stratification

Randomization will be stratified according to the following factors:

1. Pneumonia type at baseline: non-ventilated HABP vs. ventilated HABP/VABP
2. APACHE II score at baseline: <15 vs. ≥ 15

Subjects will be stratified by factors (as noted above) that are most likely to impact mortality outcomes. This will be done in order to balance treatment assignment within each stratum level and for increased efficiency of the statistical analysis.

5.5 Concomitant Medications/Vaccinations (Allowed & Prohibited)

Medications or vaccinations specifically prohibited in the exclusion criteria are not allowed during time periods specified by this protocol for that medication or vaccination. If there is a clinical indication for any medication or vaccination specifically prohibited during time periods specified by this protocol for that medication or vaccination, discontinuation from trial therapy or vaccination may be required. The investigator should discuss any questions regarding this with the Sponsor Clinical Director. The final decision on any supportive therapy or vaccination rests with the investigator and/or the subject's primary physician. However, the decision to continue the subject on trial therapy or vaccination schedule requires the mutual agreement of the investigator, the Sponsor and the subject.

The following concomitant medications/therapies <u>are not permitted</u> during IV study therapy:

1. Immunosuppressive agents

   <u>NOTE</u>: Short-term treatment with systemic (IV or oral) steroids of ≤1 week duration (e.g., treatment for an acute asthma exacerbation or acute skin condition) is allowed. Topical steroids for the treatment of skin conditions are also allowed.

2. Valproic acid or divalproex sodium (during treatment with study drug and the 2 weeks prior to screening)

3. Serotonin re-uptake inhibitors, tricyclic antidepressants, serotonin 5-HT1 receptor agonists (triptans) (during treatment with linezolid)
4. MAOIs (during treatment with linezolid and the 2 weeks prior to screening)
5. Meperidine (during treatment with linezolid)
6. Buspirone (during treatment with linezolid)

7. Non-study systemic (IV or oral) antibacterial treatments

   <u>NOTE</u>: As noted in Section 5.2, the use of initial empiric treatment with IV linezolid for MRSA infection is required. The recommended treatment duration for a confirmed MRSA infection is a minimum of 7 days, with a minimum of 14 days for MRSA bacteremia. Empiric linezolid treatment will be stopped if the final culture results from the baseline lower respiratory tract sample do not demonstrate the presence of MRSA.

8. Systemic (IV or oral) antifungal or antiviral therapy for the index infection of HABP/VABP

5.6 Rescue Medications & Supportive Care

No rescue or supportive medications are specified to be used in this trial.

5.7 Diet/Activity/Other Considerations

There are no dietary or activity restrictions in this study, except as medically indicated.

5.8 Subject Withdrawal/Discontinuation Criteria

Subjects may withdraw consent at any time for any reason or be dropped from the trial at the discretion of the investigator should any untoward effect occur. In addition, a subject may be withdrawn by the investigator or the Sponsor if enrollment into the trial is inappropriate, the trial plan is violated, or for administrative and/or other safety reasons. Specific details regarding discontinuation or withdrawal procedures; including specific details regarding withdrawal from Future Biomedical Research, are provided in Section 7.1.4 – Other Procedures.

Discontinuation from treatment is "permanent". Once a subject is discontinued, he/she shall not be allowed to restart treatment.

A subject must be discontinued from the trial for any of the following reasons:

- The subject or legal representative (such as a parent or legal guardian) withdraws consent.
- The subject has a medical condition or personal circumstance which, in the opinion of the investigator and/or Sponsor, places the subject at unnecessary risk through continued participation in the trial or does not allow the subject to adhere to the requirements of the protocol.

In this trial, a subject may discontinue from IV study therapy but should continue to participate in the regularly scheduled activities, as long as the subject does not withdraw consent. Discontinuation from IV study therapy is permanent. Once a subject has discontinued IV study therapy, even though he/she continues to be monitored in the trial, he/she shall not be allowed to begin treatment again.

A subject must be discontinued from IV study therapy (but **should** continue to be monitored in the trial) for any of the following reasons:

1. Any of the following post-baseline elevations in liver transaminase levels:

   In subjects without baseline transaminase elevations:

   - ALT or AST ≥8 X ULN
   - ALT or AST ≥3 X ULN, accompanied by total bilirubin > 2 X ULN OR INR > 1.5
   - ALT or AST ≥3 X ULN with the appearance of fatigue, nausea, vomiting, right upper quadrant pain or tenderness, fever (defined as oral or tympanic temperature ≥38.0°C [≥100.4°F]) rash, and/or eosinophilia (>5%)

   In subjects with pre-existing transaminase elevations:

   - Further increase in transaminases to ≥8X ULN **[with at least 50% increase from transaminase values collected at randomization (Day 1)]** that is not anticipated from their underlying medical condition
   - ALT or AST ≥3 X ULN **[with at least 50% increase from transaminase values collected at randomization (Day 1)]** and new onset of clinical signs and symptoms of fatigue, nausea, vomiting, right upper quadrant pain or

tenderness, fever (defined as oral or tympanic temperature ≥38.0°C [≥100.4°F]), rash, and/or eosinophilia (>5%)

- Clinically significant worsening of liver function associated with further transaminase elevations in a subject with abnormal transaminase levels already meeting the above criteria (i.e., ≥8X ULN **[with at least 50% increase from transaminase values collected at randomization (Day 1)]**; ALT or AST ≥3 X ULN **[with at least 50% increase from transaminase values collected at randomization (Day 1)]** and new onset of clinical signs and symptoms listed) at baseline.

NOTE: The subject will discontinue from study therapy, but will continue to participate in the study and will be assessed according to all study planned study procedures through the final study visit and will be evaluated for blood chemistry and hematology (including, at minimum, ALT, AST, alkaline phosphatase, bilirubin (direct + indirect) and creatine phosphokinase) until values return to within normal range. The trial site guidance for assessment and follow up of liver function test (LFT) elevations can be found in the Investigator Trial File Binder.

2. A post-baseline decline in estimated or actual creatinine clearance to a value of less than 15 mL/min.
3. The subject requires initiation of hemodialysis or peritoneal dialysis.
4. The subject has a confirmed positive serum or urine (sensitivity of < 25 mIU/L is required if using urine test) pregnancy test.
5. A physician investigator feels it is in best interest of the subject to discontinue for any reason, including, but not limited to, the need for alternative non-study antibacterial therapy.

5.9 Subject Replacement Strategy

A subject who discontinues from the trial will not be replaced.

5.10 Beginning and End of the Trial

The overall trial begins when the first subject signs the informed consent form. The overall trial ends when the last subject completes the last trial visit, discontinues from the trial or is lost to follow-up (i.e. the subject is unable to be contacted by the investigator).

5.11 Clinical Criteria for Early Trial Termination

The clinical trial may be terminated early if the extent (incidence and/or severity) of emerging effects/clinical endpoints is such that the risk/benefit ratio to the trial population as a whole is unacceptable. In addition, further recruitment in the trial or at (a) particular trial site(s) may be stopped due to insufficient compliance with the protocol, GCP and/or other applicable regulatory requirements, procedure-related problems or the number of discontinuations for administrative reasons is too high.

6.0 TRIAL FLOW CHART

Trial Period:	Screening			IV Study Treatment					Post-treatment	
Visit Title:	**Visit 1**			**Visit 2** Randomization/ Initiation of Therapy	**Visit 3** On Therapy #1 (OTX_1)	**Visit 4** On Therapy #2 (OTX_2)	**Visit 5** On Therapy #3 (OTX_3)	**Visit 6** [a] End of Treatment (EOT)	**Visit 7** [a] Early Follow-Up (EFU)	**Visit 8** [a] Day 28[b]
	Early Consent Option		Standard Consent Option							
Scheduled Day	Day -14 to -1	Day -2 to -1	Day -2 to -1	Day 1	Day 3	Day 6	Day 10	Anytime from Day 7 to Day 14[c]	7 to 14 days post EOT	Day 28 Post-Randomization
Scheduling Window:	≤ 2 weeks prior to randomiz-ation	≤ 48 hours prior to randomiz-ation	≤ 48 hours prior to randomization	N/A	N/A	N/A	N/A	≤ 24 hours after last dose of IV study therapy	+ 2 days	+ 3 days
**Administrative Procedures**										
Informed Consent (Early Consent Option)	X									
Informed Consent (Standard Consent Option)			X							
Informed Consent for Future Biomedical Research [d] (optional)	X		X							
**Inclusion/Exclusion Criteria (Early Consent Option)**										
Assess Inclusion Criteria #1, 7, 9[e]	X									
Assess Inclusion Criteria #2, 3, 4, 5, 6, 8		X								
Assess All Exclusion Criteria		X								
**Inclusion/Exclusion Criteria (Standard Consent Option)**										
Assess All Inclusion/Exclusion Criteria			X							
Subject Identification Card	X		X							
Medical History		X	X							
Prior or Concomitant Medication Review		X	X	X	X	X	X	X	X	X
Treatment Allocation/Randomization & Stratification				X						
Administration of IV Study Therapy [c]				Daily (administered IV q6h)						
Administration of Empirical IV Linezolid Therapy[f]				Daily (administered at 600 mg IV q12h)						
**Clinical Procedures/Assessments**										
APACHE II Score [g]		X	X							
Clinical Pulmonary Infection Score (CPIS)[g]		X	X							
Full Physical Examination				X						

Trial Period:	Screening			IV Study Treatment					Post-treatment	
Visit Title:	**Visit 1**			**Visit 2** Randomization/ Initiation of Therapy	**Visit 3** On Therapy #1 (OTX_1)	**Visit 4** On Therapy #2 (OTX_2)	**Visit 5** On Therapy #3 (OTX_3)	**Visit 6** [a] End of Treatment (EOT)	**Visit 7** [a] Early Follow-Up (EFU)	**Visit 8** [a] Day 28[b]
	Early Consent Option		Standard Consent Option							
Scheduled Day	Day -14 to -1	Day -2 to -1	Day -2 to -1	Day 1	Day 3	Day 6	Day 10	Anytime from Day 7 to Day 14[c]	7 to 14 days post EOT	Day 28 Post-Randomization
Scheduling Window:	≤ 2 weeks prior to randomiz-ation	≤ 48 hours prior to randomiz-ation	≤ 48 hours prior to randomization	N/A	N/A	N/A	N/A	≤ 24 hours after last dose of IV study therapy	+ 2 days	+ 3 days
Directed Physical Examination					X	X	X	X	X	X
Vital Signs (heart rate, blood pressure, respiratory rate, oral temperature)[h]				Daily during IV study therapy				X	X	X
Height[i]				X						
Weight[i]				X						
Adverse Event Monitoring [j]	X	X	X	Daily during IV study therapy				X	X	X
Local Infusion Tolerability Monitoring [j]				Daily during IV study therapy				X		
Review of Clinical Signs and Symptoms of HABP/VABP Infection [k]				Daily during IV study therapy				X	X	X
Chest X-ray [l]				X				X	X	X
PaO_2/FiO_2 Ratio or O_2 Saturation[m]				Daily during IV study therapy				X	X	X
Infection Source Control Review [n]				X	X	X	X			
**Laboratory Procedures/Assessments**										
Blood for Hematology [o]				X	X	X	X	X	X	X
Blood for Chemistry[o]				X	X	X	X	X	X	X
Blood for Local Laboratory Assessment of Creatinine[p]				X	X	X	X			
Urine for Urinalysis [q]				X				X		
Serum/urine for β-Human Chorionic Gonadotropin (β-hCG), in women of reproductive potential only [r]		X	X							X
Blood for Genetic Analysis[d]				X						
Lower Respiratory Tract Specimen for Gram stain, Culture and Susceptibility [s,t]				X [s]	X[t]	X[t]	X[t]	X	X	X[t]
Blood Specimen for Culture and Susceptibility [u]				X	As clinically indicated, or if pre-study blood culture was positive, repeat daily until negative on 2 consecutive cultures					
**Population Pharmacokinetics Analysis**										
Whole blood to obtain plasma for REL, impenem, and cilastatin assay [v]		X	X	X	X	X				

Trial Period:	Screening			IV Study Treatment					Post-treatment	
Visit Title:	**Visit 1**			**Visit 2** Randomization/ Initiation of Therapy	**Visit 3** On Therapy #1 (OTX_1)	**Visit 4** On Therapy #2 (OTX_2)	**Visit 5** On Therapy #3 (OTX_3)	**Visit 6** [a] End of Treatment (EOT)	**Visit 7** [a] Early Follow-Up (EFU)	**Visit 8** [a] Day 28[b]
	Early Consent Option		Standard Consent Option							
Scheduled Day	Day -14 to -1	Day -2 to -1	Day -2 to -1	Day 1	Day 3	Day 6	Day 10	Anytime from Day 7 to Day 14[c]	7 to 14 days post EOT	Day 28 Post-Randomization
Scheduling Window:	≤ 2 weeks prior to randomiz-ation	≤ 48 hours prior to randomiz-ation	≤ 48 hours prior to randomization	N/A	N/A	N/A	N/A	≤ 24 hours after last dose of IV study therapy	+ 2 days	+ 3 days
**Efficacy Evaluation**										
Survival Assessment									X	X
Clinical Response Assessment [w]					X	X	X	X	X	X
Microbiological Assessment (By-Pathogen) [x]								X	X	
Health Resource Utilization Measures										X

PaO_2 = partial pressure of oxygen in arterial blood; FiO_2 = Fraction of inspired oxygen

[a] Register completion of EOT, EFU, and Day 28 visits in IVRS.

[b] The Day 28 post-randomization visit may be combined with the EFU visit on a single day, as long as compliance with the visit windows is maintained for both visits. Specifically, if a subject receives 12 to 14 days of IV study therapy, the EFU visit may potentially be combined with the Day 28 post-randomization visit. For example, if 12 days of IV study therapy are provided, the EFU visit could be scheduled 16 days (14 days +2 day variance) following completion of IV study therapy, which would be 28 days (12 days of IV study therapy +16 days of follow-up) following randomization. It is required that for any case, the allowable visit window ranges are maintained.

[c] IV study therapy should be administered daily for a minimum of 7 full days. This translates to 28 doses of IMI/REL (Treatment Group 1) or PIP/TAZ (Treatment Group 2) for every 6 hour (q6h) dosing. Of note, subjects with evidence of concurrent bacteremia or with *P. aeruginosa* involvement should receive 14 days of IV study therapy.

[d] This sample should be drawn for planned analysis of the association between genetic variants in DNA and drug response. If there is either a documented law or regulation prohibiting collection, or if the IRB/IEC does not approve the collection of the sample for these purposes, then this sample will not be collected at that site. If the sample is collected, leftover extracted DNA will be stored for future biomedical research if the subject signs the FBR consent. If the planned genetic analysis is not approved, but FBR is approved and consent is given, this sample will be collected for the purpose of FBR.

[e] If early screening criteria are met and the subject goes on to develop signs and symptoms of pneumonia, the subject will qualify to be assessed for disease eligibility and all other study entry criteria, which must be assessed and confirmed within 48 hours prior to randomization.

[f] The recommended treatment duration of IV linezolid at the 600 mg q12h dose for a confirmed MRSA infection is a minimum of 7 days, with a minimum of 14 days for MRSA bacteremia. Empiric linezolid treatment will be stopped if the final culture results from the baseline lower respiratory tract sample do not demonstrate the presence of MRSA

[g] Please refer to Appendix 12.5 and Appendix 12.6 for the parameters and instructions for calculation of APACHE II score and CPIS, respectively.

[h] Vital signs should be assessed and recorded daily during IV study therapy. All findings should be documented on the appropriate electronic case report (eCRF) forms. Collection at randomization should be performed **prior to** initiation of IV study therapy

[i] Collection at randomization should be performed **prior to** initiation of IV study therapy.

[j] Monitor for adverse events and local infusion tolerability daily during IV study therapy and adverse events for 14 days after completion of IV study therapy (including through EFU visit and Day 28 post-randomization visit). Events that emerge during IV study therapy and are considered as related to IV study therapy by the investigator must be followed until resolved or stabilized. Serious adverse events (SAE) that are considered by the investigator to be possibly, probably, or definitely related (all considered related to study drug) to the investigational product that is brought to the attention of the investigator at any time outside of the EFU also must be reported immediately to the Sponsor. Please refer to Section 7.2 for further details, including reporting of select adverse events that occur after the consent form is signed but before treatment randomization.

k Infection-specific clinical signs and symptoms, radiographic (daily reporting not required for radiographic findings) and laboratory assessments (see Table 10, Section 7.1.2.7) will be collected daily during IV study therapy and the additional visits specified . All findings should be documented on the appropriate eCRF forms. Collection at randomization (Visit 2) should be performed **prior to** initiation of IV study therapy.

l A baseline chest x-ray (prior to initiation of IV study therapy) should only be performed if a prior chest x-ray has not been performed in association with the current infection within 48 hours of randomization. Relevant radiographic findings should be documented on the appropriate eCRF forms.

m PaO_2 and FiO_2 measured via ABG (in ventilated subjects who have an existing arterial line) or oxygen saturation via pulse oximetry (in all other subjects) must all be measured on Day 1, Day 3 (OTX1), Day 6 (OTX2), and Day 10 (OTX3, if applicable) of IV study therapy, and at the EOT, EFU, and Day 28 post-randomization visits. On all other days of IV study therapy, oxygen saturation via pulse oximetry should be measured. All measured values should be documented on the appropriate eCRF forms.

n Relevant information regarding infection source control should be documented on the appropriate eCRF. Source control includes the following: (a) details regarding intubation, extubation, reintubation, or replacement of the endotracheal tube, (b) details regarding lung procedures/surgeries performed to drain/remove a loculated pulmonary infection, or (c) details regarding a thoracentesis procedure to drain any accompanying pleural fluid.

o Laboratory safety tests collected from blood (hematology and chemistry) should be performed at the randomization visit (Day 1) prior to administration of the first dose of IV study therapy, on Day 3 of IV study therapy (OTX1), Day 6 of IV study therapy (OTX2), and Day 10 of IV study therapy (OTX3, if applicable),. Laboratory safety tests of blood (hematology and chemistry) should also be performed at the EOT, EFU, and Day 28 post-randomization visits. Laboratory safety tests of urine (urinalysis) should be performed at randomization and EOT. Specific safety laboratory tests of blood are included in Section 7.1.3.1, Table 11.

p For the purpose of monitoring renal function during IV treatment, a creatinine assessment should also be performed at the local laboratory on Days 1, 3, 6, and 10 prior to administration of IV study therapy. For subjects with renal insufficiency or whose creatinine clearance changes during treatment with study therapy (refer to Table 8 and Table 9 in Section 5.2.1.1), the dose of study drug must be adjusted based upon the degree of renal function impairment as determined by the estimated or actual creatinine clearance. Only those local laboratory abnormalities in serum creatinine that resulted in an adverse event or a clinically significant change in creatinine clearance which may or may not result in a dose adjustment should be collected on the appropriate eCRF. Please note only the unblinded pharmacist of designee will know if the change in creatinine clearance resulted in a dose adjustment. Blinded study staff will remain blinded.

q The urinalysis should be performed on mid-stream clean catch urine or catheter urine specimen, if possible. Specific safety laboratory tests performed as part of the urinalysis are included in Section 7.1.3.1, Table 11.

r Prior documentation of a negative serum β-HCG within 48 hours of enrollment is acceptable for women of reproductive potential. If documentation is not available, a rapid urine β-HCG (dipstick) may be used for screening; however, a serum β-HCG must be collected and sent to the Central Laboratory for confirmation of the dipstick result. If the serum β-HCG test comes back positive from the Central Laboratory, the subject must be discontinued. To conduct urine testing, sites must have individuals certified in administration and interpretation of test and the urine test utilized must have sensitivity of < 25 mIU/L. The sample collected at the Day 28 post-randomization visit should be a serum β-HCG.

s Obtain lower respiratory tract sample for Gram-stain, culture, and susceptibility testing from infection site **prior to** initiation of IV study therapy for all subjects. A previously obtained culture is acceptable if it was obtained within 48 hours of the screening visit. Microscopic examination of Gram stained smears must be performed prior to randomization to ensure the adequacy of the specimen. The subject must be excluded from participating in the trial if the Gram stain shows the presence of Gram-positive cocci **only**. All culture and susceptibility should be performed at the local microbiology laboratory per local standards; in addition, any suspected causative bacterial pathogen must be collected and available for submission to the Central Microbiology Reference Laboratory. Suspected causative bacterial pathogens should also be stored at the local microbiology laboratory if possible future testing is needed. In addition, the available data from the Gram-stain and culture specimen, including date of collection, specimen type (including method of collection), and pathogen identification (to the species level, if identified) must be collected on the appropriate eCRF forms.

t Culture from the lower respiratory tract site, including susceptibility testing of any identified pathogens on culture, should be performed at these visits if there is clinical or laboratory evidence of persistence or progression of the infectious process (including persistent fever, elevated white blood cell count, or significant changes in the subject's clinical condition). Of note, specimens should also be collected at any time of surgical or drainage procedure (if required). All culture and susceptibility should be performed at the local microbiology laboratory per local standards; in addition, any suspected causative bacterial pathogen must be collected and available for submission to the Central Microbiology Reference Laboratory. Suspected causative bacterial pathogens should also be stored at the local microbiology laboratory if possible future testing is needed. In addition, the available data from the culture specimen, including date of collection, specimen type (including method of collection), and pathogen identification (to the species level, if identified) must be collected on the appropriate eCRF forms.

u Two sets of blood cultures, from two separate venipuncture (or 1 venipuncture and 1 catheter) sites must be collected in all subjects prior to initiation of IV study therapy. Subjects with positive blood cultures at screening should have follow-up blood cultures collected daily until 2 consecutive cultures demonstrate no growth. Blood culture and susceptibility will be performed at the local microbiology laboratory per local standards; in addition, any suspected causative bacterial pathogen must be collected and available for submission to the Central Microbiology Reference Laboratory. Suspected causative bacterial pathogens should also be stored at the local microbiology laboratory if possible future testing is needed. Relevant culture data, including date of collection, specimen type (including method of collection), and pathogen identification (to the species level, if identified) must be collected on the appropriate eCRF forms.

v Whole blood (4 mL) to obtain plasma samples for determination of REL, imipenem, and cilastatin plasma concentrations will be collected at screening and on Day 1 (randomization), Day 3 (OTX1), and Day 6 (OTX2) of IV study therapy at 2 time points: (1) at approximately 30 minutes post-start of first IV drug infusion, and (2) at approximately 4 hours post-start of first IV drug infusion. If it is not feasible to collect PK samples post-start of first IV drug infusion on Day 3 and Day 6, samples may be collected with a later infusion within a 24 hour period. Both time points

should be collected after the same dose (i.e., 30 minutes and 4 hours post Dose #3). Whole blood sample collection procedures are provided in a separate Laboratory Manual. Sample handling, processing, and shipment procedures will be provided separately in a Laboratory Manual. Actual whole blood collection date and times should be recorded on the appropriate eCRF forms.

[w] The presence and/or absence of infection-specific clinical signs and symptoms will be evaluated by the investigator at each specified visit compared with baseline signs and symptoms to determine the clinical response assessment (see Section 4.2.3.1.1.2, Table 2,Table 3, and Table 4). Relevant clinical signs and symptoms are included in Inclusion Criterion #3.

[x] A by-pathogen microbiological response rating will be determined by the investigator at EOT and EFU visits based on the local laboratory results of lower respiratory tract cultures collected for subjects at each of these visit. Please refer to Section 4.2.3.1.1.3 for additional details.

7.0 TRIAL PROCEDURES

7.1 Trial Procedures

The Trial Flow Chart - Section 6.0 summarizes the trial procedures to be performed at each visit. Individual trial procedures are described in detail below. It may be necessary to perform these procedures at unscheduled time points if deemed clinically necessary by the investigator.

Furthermore, additional evaluations/testing may be deemed necessary by the investigator and or the Sponsor for reasons related to subject safety. In some cases, such evaluation/testing may be potentially sensitive in nature (e.g., HIV, Hepatitis C, etc.), and thus local regulations may require that additional informed consent be obtained from the subject. In these cases, such evaluations/testing will be performed in accordance with those regulations.

7.1.1 Administrative Procedures

7.1.1.1 Informed Consent

The investigator or qualified designee must obtain documented consent from each potential subject or each subject's legally acceptable representative prior to participating in a clinical trial or Future Biomedical Research.

7.1.1.1.1 General Informed Consent

Consent must be documented by the subject's dated signature or by the subject's legally acceptable representative's dated signature on a consent form along with the dated signature of the person conducting the consent discussion.

A copy of the signed and dated consent form should be given to the subject before participation in the trial.

The initial informed consent form, any subsequent revised written informed consent form and any written information provided to the subject must receive the IRB/ERC's approval/favorable opinion in advance of use. The subject or his/her legally acceptable representative should be informed in a timely manner if new information becomes available that may be relevant to the subject's willingness to continue participation in the trial. The communication of this information will be provided and documented via a revised consent form or addendum to the original consent form that captures the subject's dated signature or by the subject's legally acceptable representative's dated signature.

Specifics about a trial and the trial population will be added to the consent form template at the protocol level.

The informed consent will adhere to IRB/ERC requirements, applicable laws and regulations and Sponsor requirements.

7.1.1.1.2 Consent and Collection of Specimens for Future Biomedical Research

The investigator or qualified designee will explain the Future Biomedical Research consent to the subject, answer all of his/her questions, and obtain written informed consent before performing any procedure related to the Future Biomedical Research sub-trial. A copy of the informed consent will be given to the subject.

7.1.1.2 Inclusion/Exclusion Criteria

All inclusion and exclusion criteria will be reviewed by the investigator or qualified designee to ensure that the subject qualifies for the trial.

7.1.1.3 Subject Identification Card

All subjects will be given a Subject Identification Card identifying them as participants in a research trial. The card will contain trial site contact information (including direct telephone numbers) to be utilized in the event of an emergency. The investigator or qualified designee will provide the subject with a Subject Identification Card immediately after the subject provides written informed consent.

The subject identification card also contains contact information for the emergency unblinding call center so that a health care provider can obtain information about trial medication/vaccination in emergency situations where the investigator is not available.

7.1.1.4 Medical History

A medical history will be obtained by the investigator or qualified designee. In addition to the evaluation of a subject's medical history in terms of study eligibility, all medical conditions present during the 12 months prior to study entry will be documented at the screening visit on the appropriate eCRF.

Any history of prior HABP or VABP episodes or conditions that may predispose a subject to the development of a pulmonary infection will also be documented on the appropriate eCRF, even if the prior episode or predisposing condition was diagnosed more than 12 months prior to study entry.

A full evaluation of the current primary diagnosis (HABP/VABP) will also be performed. The details of the HABP/VABP diagnosis will be documented separately on the appropriate eCRF(s).

7.1.1.5 Prior and Concomitant Medications Review

7.1.1.5.1 Prior Medications

The investigator or qualified designee will review prior medication use, and record all prior medication taken by the subject within 14 days and any antimicrobial medications taken within 30 days before starting the trial.

Following a diagnosis HABP/VABP and prior to the receipt of first dose of IV study therapy, ***subjects may receive no more than 24 hours*** (for example, >1 dose of a once daily antibiotic, >2 doses of a twice daily antibiotic) of active non-study antibiotic therapy in the

72 hours preceding the first dose of IV study therapy for treatment of the current index infection.

Subjects who have failed prior antibiotic therapy for the current episode of HABP/VABP (i.e., have persistent/worsening signs and symptoms at the time of screening) may be considered for enrollment provided the prior antibiotic was given for at least 48 hours before a diagnosis of clinical failure was made and the prior respiratory or blood culture did not grow only *S. aureus* (methicillin-susceptible *S. aureus* [MSSA] or methicillin-resistant S. aureus [MRSA]). For such subjects, the baseline LRT culture must be obtained while the subject is on the failing antibiotic therapy and before the subject receives the first dose of IV study therapy. Following collection of the baseline LRT culture, only IV study therapy is permitted in these subjects.

Subjects receiving prior therapy with a non-absorbed antibiotic used for gut decontamination (example, low dose erythromycin or polymyxin) or to eradicate *C. difficile* (oral vancomycin or fidaxomicin) are eligible for enrollment, irrespective of duration of prior antibacterial therapy.

7.1.1.5.2 Concomitant Medications

The investigator or qualified designee will record medication, if any, taken by the subject during the trial.

As the study will enroll subjects without confirmed microbiological (culture) evidence of the HABP/VABP pathogen on a lower respiratory tract specimen at study entry, the use of initial empiric treatment with open-label IV linezolid (600 mg q12h) for methicillin-resistant *S. aureus* (MRSA) infection is required. The recommended treatment duration for a confirmed MRSA infection is a minimum of 7 days, with a minimum of 14 days for MRSA bacteremia. Empiric linezolid treatment will be stopped if the final culture results from the baseline lower respiratory tract sample do not demonstrate the presence of MRSA.

7.1.1.6 Assignment of Screening Number

All consented subjects will be given a unique screening number that will be used to identify the subject for all procedures that occur prior to randomization or allocation. Each subject will be assigned only one screening number. Screening numbers must not be re-used for different subjects.

Rescreening of an individual subject for enrollment is not expected to occur commonly. However, any subject who is screened multiple times will retain the original screening number assigned at the initial screening visit.

7.1.1.7 Assignment of Randomization Number

All eligible subjects will be randomly allocated and will receive a randomization number. The randomization number identifies the subject for all procedures occurring after randomization. Once a randomization number is assigned to a subject, it can never be re-assigned to another subject.

A single subject cannot be assigned more than 1 randomization number.

Randomization will be stratified according to the following factors, as described in Section 5.4: (1) Pneumonia type at baseline: non-ventilated HABP vs. ventilated HABP/VABP; and (2) APACHE II score at baseline: < 15 vs. ≥ 15. .

7.1.1.8 Trial Compliance (Medication/Diet/Activity/Other)

Interruptions from the protocol specified specified IV study therapy treatment regimen totaling greater than or equal to 4 doses of IV study therapy during the first 7 days of therapy OR greater than or equal to 4 doses of IV study therapy during days 8 to 14 of therapy require consultation between the investigator and the Sponsor and written documentation of the collaborative decision on subject management.

Administration of trial medication will be performed by the blinded qualified trial site personnel.

7.1.1.9 Study Therapy Administration

All IV study therapy will be reconstituted and administered according to the details provided in a separate Pharmacy Manual. Dosing regimens for IMI/REL (Treatment Group 1) and PIP/TAZ (Treatment Group 2) are described in Section 5.2. The specific dosage as well as frequency and timing of administration for IMI/REL and PIP/TAZ is provided in Sections 5.2.1.1.1 and 5.2.1.1.2, respectively.

An Unblinded Study Pharmacist (or qualified designee) at the study site will be responsible for preparing the IV study therapy for this study; this individual(s) must not be involved in any of the safety and efficacy evaluations of the study participants. Due to a visual difference in the appearance of study treatment solutions, the infusion bags will be covered with an opaque sleeve by the Unblinded Study Pharmacist (or qualified designee) to ensure that other study personnel and all subjects remain blinded to clinical material assignments.

All IV study therapy infusions should be administered by the blinded investigator and/or blinded trial staff over 30 minutes +/- 10 minutes. The IV study therapy must NOT be administered simultaneously through the same infusion line/lumen with any other drugs (including IV non-study drugs). If another IV drug is required either prior to or after study drug and only 1 line is available, an appropriate volume of saline flush must be used between IV infusions.

Further details on the preparation, storage, and administration of all intravenous study antibiotics by the Unblinded Study Pharmacist (or qualified designee) are provided in a separate Pharmacy Binder.

IV study therapy should be administered for a minimum of 7 full days to up to a maximum of 14 days. Subjects with evidence of concurrent bacteremia or with P. *aeruginosa* involvement should receive 14 days of treatment.

7.1.1.10 Linezolid Therapy Administration

Since the study will enroll subjects without confirmed microbiological (culture) evidence of the HABP/VABP pathogen from a lower respiratory tract specimen at study entry, the use of initial empiric treatment with IV open-label linezolid (600 mg q12h) for methicillin-resistant *S. aureus* (MRSA) infection is required. The recommended treatment duration for a confirmed MRSA infection is a minimum of 7 days, with a minimum of 14 days for MRSA bacteremia. Empiric linezolid treatment will be stopped if the final culture results from the baseline lower respiratory tract sample do not demonstrate the presence of MRSA.

Linezolid therapy can be administered concomitantly with IV study therapy, at those time points where there is administration overlap; however, the IV study therapy and IV linezolid therapy must NOT be administered simultaneously through the same infusion line/lumen.

7.1.2 Clinical Procedures/Assessments

7.1.2.1 APACHE II Score

Severity of illness in this study will be determined by APACHE II score at screening [20]. See Appendix 12.5 for details regarding the calculation of this score. Results of APACHE II score calculations must be entered on the appropriate eCRF(s).

7.1.2.2 Clinical Pulmonary Infection Score (CPIS)

The CPIS will be calculated based on clinical signs and symptoms of pneumonia at screening [21]. See Appendix 12.6 for details regarding the calculation of this score. Results of CPIS must be entered on the appropriate eCRF(s).

7.1.2.3 Full and Directed Physical Examinations

All physical examinations must be performed by the principal investigator or sub-investigator (physician, physician assistant or nurse practitioner).

A full physical examination, performed at randomization includes the following assessments: general appearance, head, eyes, ears/nose/ throat, neck, lymph nodes, skin, lungs, heart, abdomen, musculoskeletal, and neurologic evaluations. Breast, rectal, and genitourinary/pelvic exams should be performed when clinically indicated. If a physical examination was performed within 72 hours prior to screening, those results can be recorded and a repeat physical examination is not required. Any abnormal or clinically significant findings from the physical examinations must be recorded on the appropriate eCRF.

After the initial full physical exam, a physical exam targeted to the subject's illness and complaints will be performed at subsequent visits as specified in Section 6.0 (Trial Flow Chart).

7.1.2.4 Vital Signs

Vital signs should be collected daily while on IV study therapy and at other time points/visits as specified in Section 6.0 (Trial Flow Chart). Collection at randomization (Visit 2) should be performed **prior to** initiation of IV therapy.

For this study, vital signs include heart rate (HR), blood pressure (BP), respiratory rate (RR), and oral temperature. Subjects should be resting in a seated or semi-recumbent position for at least 10 minutes prior to having vital sign measurements obtained. For subjects who are intubated and cannot sit up, HR, BP, and RR may be taken in a supine or semi-recumbent position. Oral temperatures should be taken, but if oral is not possible, tympanic, rectal, or axillary methods are acceptable.

Any abnormal or clinically significant findings from the physical examinations must be recorded on the appropriate eCRF.

7.1.2.5 Height and Weight

The subject's height and weight should be measured prior to initiation of IV study therapy at the randomization (Day 1) visit. Collection at randomization (Visit 2) should be performed **prior to** initiation of IV treatment

7.1.2.6 Adverse Event Monitoring and Local Infusion Tolerability Monitoring

Clinical adverse events will be collected daily from the time of initiation of the first dose of IV study therapy through 14 days following completion of all IV study therapy (including through EFU visit and Day 28 post-randomization visit). All adverse events should be documented on the appropriate eCRF. In general, events that emerge during IV study therapy and are considered as related to IV study therapy by the investigator must be followed until resolved or stabilized. Serious adverse events (SAE) that are considered by the investigator to be possibly, probably, or definitely related (i.e., all considered as related to study drug) to the investigational product that is brought to the attention of the investigator at any time outside of the EFU also must be reported immediately to the Sponsor.

In addition, local infusion site tolerability will be evaluated daily during IV study therapy. The tolerability of all study therapy at the local IV infusion site will be based on investigator inspection and subject comments regarding signs and symptoms of intolerance. The IV infusion site should be observed daily during IV therapy to determine the presence/absence of erythema, induration, pain, tenderness, warmth, swelling, ulceration, local phlebitis, rash, or other reactions. All events should be documented on the appropriate eCRF.

Laboratory adverse events will be based on safety laboratory tests, including hematology and chemistry tests from blood and urinalysis from urine. Please refer to Section 6.0 and Section 7.1.3 for more details on type of tests and timing of collection.

Please refer to Section 7.2 for details regarding assessment and documentation of adverse events.

7.1.2.7 Clinical Signs and Symptoms

A detailed diagnosis as well as relevant clinical information associated with the HABP/VABP diagnosis including clinical signs and symptoms, radiographic and laboratory characteristics related to the subject's infection will be reviewed and documented on the appropriate eCRFs at time points specified in Section 6.0 (Trial Flow Chart).

In particular, clinical signs and symptoms specific to the index infection for this study are based predominantly on criteria outlined in Inclusion Criteria #3 (Section 5.1.2) and are summarized in Table 10 below. Presence or absence of these symptoms will be recorded daily while on IV study therapy and at visits as specified in Section 6.0 (Trial Flow Chart). Intensity of signs and symptoms will also be graded by the investigator as mild, moderate, or severe (See Section 7.2). Collection at randomization (Visit 2) should be performed **prior to** initiation of IV study therapy.

Table 10 Infection-Site Clinical Signs and Symptoms

**Infection Site**	**Clinical Signs and Symptoms**
HABP/VABP	• Cough<br>• Dyspnea<br>• Tachypnea (e.g., respiratory rate greater than 25 breaths per minute)<br>• Expectorated sputum production<br>• Requirement for mechanical ventilation<br>• New onset of suctioned respiratory secretions<br>• Chills/rigors<br>• Chest pain or chest tenderness<br>• Fever (body temperature ≥ 38 degrees Celsius)<br>• Hypothermia (core body temperature ≤35 degrees Celsius

7.1.2.8 Chest X-Ray

A baseline chest x-ray should be performed in all subjects prior to initiation of IV study therapy on the Day 1 (Randomization) visit and at other visits as specified in Section 6.0 (Trial Flow Chart). A baseline chest x-ray at randomization is not required if a prior chest x-ray was performed in association with the current infection within 48 hours of randomization.

Chest x-ray results at baseline (prior to initiation of IV study therapy) and at indicated follow-up visits including a description, location, and extent of infiltrates or consolidation must be documented on the appropriate eCRF(s). The presence of a pleural effusion and other abnormalities associated with disease-related complications should also be noted on the appropriate eCRF(s).

7.1.2.9 PaO_2/FiO_2 OR O_2 Saturation

PaO_2 and FiO_2 should be measured by arterial blood gas (ABG) determination and oxygen saturation should be determined by pulse oximetry or by ABG.

For ventilated subjects who have an existing arterial line in place, PaO_2 and FiO_2 should be measured by ABG on Day 1, Day 3 (OTX1), Day 6 (OTX2), and Day 10 (OTX3, if applicable) of IV study therapy, and at the EOT, EFU, and Day 28 post-randomization visits. On all other days while on IV study therapy, oxygen saturation via pulse oximetry should be measured daily.

For non-ventilated subjects or ventilated subjects who do not have an existing arterial line in place, oxygen saturation via pulse oximetry should be measured daily while on IV study therapy and at the EOT, EFU, and Day 28 post-randomization visits.

All measured values for PaO_2, FiO_2, or oxygen saturation should be documented on the appropriate eCRFs.

7.1.2.10 Infection Source Control Review

Information related to infection source control must be collected for all subjects at the visits specified in Section 6.0 (Trial Flow Chart).

Source control includes the following: (a) details regarding intubation, extubation, reintubation, or replacement of the endotracheal tube, (b) details regarding lung procedures/surgeries performed to drain/remove a loculated pulmonary infection, or (c) details regarding a thoracentesis procedure to drain any accompanying pleural fluid. Relevant information regarding infection source control should be documented on the appropriate eCRF.

7.1.3 Laboratory Procedures/Assessments

Details regarding specific laboratory procedures/assessments to be performed in this trial are provided below. The total amount of blood/tissue to be drawn/collected over the course of the trial (from pre-trial to post-trial visits), including approximate blood/tissue volumes drawn/collected by visit and by sample type per subject can be found in Appendix 12.4.

7.1.3.1 Laboratory Safety Evaluations (Hematology, Chemistry and Urinalysis)

Laboratory tests for hematology, chemistry and urinalysis are specified in Table 11.

Table 11 Laboratory Tests

Hematology	Chemistry	Urinalysis	Other
Hematocrit	Albumin	Blood	Rapid urine β-human chorionic gonadotropin (β-hCG)
Hemoglobin	Alkaline phosphatase	Glucose	Serum β-hCG
Platelet count	Alanine aminotransferase (ALT)	Protein	
WBC (total and differential)	Aspartate aminotransferase (AST)	Specific gravity	
	Bicarbonate	Microscopic exam, if abnormal results are noted	
	Blood Urea Nitrogen		
	Calcium		
	Chloride		
	Creatinine		
	Glucose		
	Phosphorus		
	Potassium		
	Sodium		
	Total Bilirubin (direct bilirubin also assessed if total bilirubin is elevated above the upper limit of normal range)		
	Total protein		

With the exception of the rapid urine β-hCG (dipstick), all blood and urine samples for safety laboratory testing (hematology, chemistry and urinalysis) will be sent to a central safety laboratory for testing. Additional details regarding biological specimen processing, handling, and shipment will be provided by the Sponsor in a separate laboratory manual.

The timing for the collection of blood and urine samples for safety monitoring is provided in Section 6.0 (Trial Flow Chart).

Local Laboratory Monitoring for Renal Function

Safety laboratory results from the central laboratory will likely not be available in a timely fashion to impact on an individual subject's medical management. Therefore, additional laboratory tests required for adequate medical management of individual study participants should be obtained as indicated by the primary physician and submitted to the local laboratory for testing in the medically appropriate timeframe.

Specifically, for the purpose of monitoring–an individual subject's renal function in "real time", a creatinine assessment should be performed at the local laboratory on Days 1, 3, 6, and 10 during IV study therapy in addition to the chemistry safety panel performed at the central laboratory. **For subjects with renal insufficiency or whose creatinine clearance changes during treatment with study therapy (refer to Table 8 and Table 9 in Section 5.2.1.1), the dose of study drug must be adjusted based upon the degree of renal function impairment as determined by the estimated or actual creatinine clearance.**

Results of these local laboratory tests must be documented in the appropriate eCRF. Laboratory abnormalities resulting in an adverse event or dose adjustment should also be collected on the appropriate eCRF. Any laboratory test abnormality that emerged during study therapy and was considered by the investigator to be an adverse event or event of clinical interest should be repeated until the abnormal value has normalized, stabilized, or returned to baseline.

7.1.3.2 Culture and Susceptibility Testing

7.1.3.2.1 Lower Respiratory Tract Sample Collected at Baseline

A lower respiratory tract sample will be obtained at baseline for Gram stain, culture and susceptibility testing from infection site **prior to** initiation of IV study therapy for all subjects. A previously obtained culture is acceptable if it was obtained within 48 hours of the screening visit. Culture and susceptibility should be performed at the local microbiology laboratory.

Microscopic examination of Gram stained smears must be performed prior to randomization to ensure the adequacy of the specimen and to exclude subjects with gram-positive cocci only detected. For specimens obtained by direct sampling of the lower respiratory tract (i.e., sampling via BAL, mini BAL or PBS), no predefined requirements are required to ascertain the quality of the respiratory specimen. However, for specimens not obtained by direct sampling of the lower respiratory tract, such as those obtained by expectorated sputum, the low-power microscopic view of the Gram stain can be used to ascertain the quality of the respiratory specimen which helps to ensure that the respiratory specimen sent for culture does not represent oropharyngeal contamination (e.g., fewer than 10 squamous epithelial cells and greater than 25 neutrophils is an example of an adequate expectorated/suctioned sputum specimen). In addition, a high-power microscopic view of the Gram stain can be used to characterize the general type of bacteria causing the pneumonia. Specimens should be processed for Gram stain and culture at the local microbiology laboratory according to recognized methods and per each laboratory's standard procedures. **In addition to local laboratory testing, a pure isolate of any suspected causative bacterial pathogen(s) must be submitted to the Central Microbiology Reference Laboratory. Suspected causative bacterial pathogens should also be stored at the local laboratory for possible future testing.**

The available data from the Gram stain and culture specimen, including date of collection, specimen type (including method of collection), and pathogen identification (to the species level, if identified) must be collected on the appropriate eCRF forms.

Additional details regarding bacterial specimen collection, processing, handling, and shipment will be provided by the Sponsor in a separate microbiology laboratory manual.

7.1.3.2.2 Other Lower Respiratory Tract Samples Following Randomization

Culture from the lower respiratory tract site, including susceptibility testing of any identified pathogens on culture, should also be performed at the EOT and EFU visits as outlined in Section 6.0 (Trial Flow Chart). Lower respiratory tract cultures would preferentially include samples collected from a tracheostomy or endotracheal aspirates, or bronchoscopy specimens. Expectorated or induced sputum samples are also accepted provided the sample does not represent oropharyngeal contamination (e.g., sample contains fewer than 10 squamous epithelial cells).

In addition, at other times during the study, lower respiratory tract samples may also be collected if there is clinical or laboratory evidence of persistence or progression of the infectious process (including persistent fever, elevated white blood cell count, or significant changes in the subject's clinical condition). Of note, specimens should also be collected at any time of surgical or drainage procedure (if required).

All culture and susceptibility should be performed at the local microbiology laboratory following the same standard procedures as used for the baseline lower respiratory tract sample. **In addition, a pure isolate of any suspected causative bacterial pathogen(s) must be submitted to the Central Microbiology Reference Laboratory. Suspected causative bacterial pathogens should also be stored at the local laboratory for possible future testing, if needed.**

The available data from the Gram stain and culture specimen, including date of collection, specimen type (including method of collection), and pathogen identification (to the species level, if identified) must be collected on the appropriate eCRF forms.

Additional details regarding bacterial specimen collection, processing, handling, and shipment will be provided by the Sponsor in a separate microbiology laboratory manual.

7.1.3.2.3 Blood Cultures

Two sets of blood cultures, from two separate venipuncture (or 1 venipuncture and 1 catheter) sites must be collected in <u>all</u> subjects prior to initiation of IV study therapy. Subjects with positive blood cultures at screening should have follow-up blood cultures collected daily until 2 consecutive cultures demonstrate no growth. Follow-up up blood cultures in subjects with no evidence of bacteremia (i.e., positive blood cultures) at study entry should also be performed, at the investigator discretion, as clinically indicated.

Blood culture and susceptibility will be performed at the local microbiology laboratory according to recognized methods and per each laboratory's standard procedures. In addition, any suspected causative bacterial pathogen must be collected and available for submission to the Central Microbiology Reference Laboratory. Suspected causative bacterial pathogens should also be stored at the local microbiology laboratory if possible future testing is needed.

Relevant culture data, including date of collection, specimen type (including method of collection), and pathogen identification (to the species level, if identified) must be collected on the appropriate eCRF forms.

7.1.3.2.4 Other Relevant Culture Samples

During the study, other pulmonary samples, including pleural fluid or direct lung samples, or samples from other distant sites of infection may also be collected if there is clinical or laboratory evidence of persistence or progression of the infectious process (including persistent fever, elevated white blood cell count, or significant changes in the subject's clinical condition). In general, for any pulmonary procedure, specimens should also be collected at any time of surgical or drainage procedure (if required).

In these situations, relevant culture data, including date of collection, specimen type (including method of collection), and pathogen identification (to the species level, if identified) must be collected on the appropriate eCRFs.

7.1.3.3 Pharmacokinetic/Pharmacodynamic Evaluations

Whole blood to obtain plasma samples will be collected for pharmacokinetic evaluation of REL, imipenem, and cilastatin concentrations. These samples may also be used for pharmacokinetic/pharmacodynamic analyses associated with efficacy and safety data.

As outlined in Section 6.0 (Trial Flow Chart), whole blood (4 mL) should be collected at screening and on Day 1 (randomization), Day 3 (OTX1), and Day 6 (OTX2) of IV study therapy at 2 time points: (1) at approximately 30 minutes post-start of first IV drug infusion, and (2) at approximately 4 hours post-start of first IV drug infusion. If it is not feasible to collect PK samples post-start of first IV drug infusion on Day 3 and Day 6, samples may be collected with a later infusion within a 24 hour period. Both time points should be collected after the same dose (i.e., 30 minutes and 4 hours post-start of Dose #3).

Actual date and time for the whole blood samples must be recorded in the eCRFs.

7.1.3.3.1 Blood Collection for Plasma for REL, Imipenem, and Cilastatin

Sample collection, storage, and shipment instructions for plasma samples will be provided in a separate operations/laboratory manual.

7.1.3.4 Planned Genetic Analysis Sample Collection

Sample collection, storage and shipment instructions for Planned Genetic Analysis samples will be provided in a separate laboratory manual.

7.1.3.5 Future Biomedical Research

The following specimens are to be obtained as part of Future Biomedical Research:

- Leftover DNA for future research

7.1.3.6 Efficacy Evaluation

7.1.3.6.1 Survival Assessment

For each subject, survival status (i.e., whether the subject is alive or dead) will be assessed at Day 28 post-randomization. Results of the assessment, including date and cause of death if relevant, will be recorded on the appropriate eCRF.

7.1.3.6.2 Clinical Response

Clinical signs and symptoms of infection (e.g., cough, dyspnea, fever, etc.) will be assessed at visits specified in Section 6.0 (Trial Flow Chart) in support of determination of a clinical response rating for each subject. A detailed list of disease-specific signs and symptoms in support of evaluation of the clinical response rating are included in Section 7.1.2.7, Table 10. Based on comparison to baseline clinical signs and symptoms of the subject's infection, the investigator will determine and record the clinical response rating at each visit as described in Section 4.2.3.1.1.2 (see Table 2, Table 3 and Table 4.)

7.1.3.6.3 Microbiological Response

Microbiological response will be evaluated separately for each lower respiratory tract pathogen isolated in the baseline culture (i.e., by-pathogen). The by-pathogen response rating determined by the investigator will be assessed based on local laboratory results.

A by-pathogen microbiological response rating will be determined by the investigator at EOT, and EFU visits based on the results of lower respiratory tract cultures collected for subjects at each of these visits relative to the pathogen(s) isolated at baseline/admission as described in Table 5 (for EOT visit) and Table 6 (for EFU visit) in Section 4.2.3.1.1.3. Lower respiratory tract cultures would preferentially include samples collected from a tracheostomy or endotracheal aspirates, or bronchoscopy specimens. Expectorated or induced sputum samples are also accepted provided the sample does not represent oropharyngeal contamination (i.e., sample contains fewer than 10 squamous epithelial cells and greater than 25 neutrophils on low power microscopy review of the Gram stain).

7.1.3.6.4 Disease-Related Complications

As a component of the second exploratory efficacy objective, disease-related complications (e.g., empyema, onset of acute respiratory distress syndrome, bacteremia) will be assessed through Day 28 post-randomization.

A full list of the complications is being developed in close collaboration with the Foundation of the National Institute of Health (FNIH) Biomarkers Consortium for HABP/VABP Efficacy Endpoints. The full list will be defined in a memo which will be authored prior to the database lock for the study. As a result, for this ongoing assessment, the disease-related complication terms will be identified based on serious adverse events (SAE) presented in the appropriate eCRF or based on microbiology data reported in the appropriate eCRF. Hence, no further data collection tools or study-related procedures will be anticipated or required for this assessment.

7.1.3.6.5 Health Resource Utilization Measures

In an effort to address an exploratory objective, several health resource utilization variables will be collected in the study, including duration in the hospital (length of stay), duration on mechanical ventilation, and duration in the ICU.

These measures will be collected at the Day 28 post-randomization visit on the appropriate eCRF; however, if the subject is discontinued from the study prior to this time point, these events would still be collected up through the time of study discontinuation.

7.1.3.6.5.1 Length of Stay in Hospital

The number of days spent in the hospital post-randomization will be recorded on the appropriate eCRF for each subject.

7.1.3.6.5.2 Duration of Mechanical Ventilation

The number of days spent on mechanical ventilation post-randomization will be recorded on the appropriate eCRF for those subjects who were randomized to the ventilated HABP/VABP stratum.

7.1.3.6.5.3 ICU Length of Stay

The number of days spent in the intensive care unit post-randomization will be recorded on the appropriate eCRF for those subjects who were randomized to the ventilated HABP/VABP stratum.

7.1.4 Other Procedures

7.1.4.1 Withdrawal/Discontinuation

Subjects who discontinue/withdraw from IV study therapy prior to completion of the IV study therapy regimen should continue to be followed for all remaining study visits.

When a subject discontinues/withdraws from participation in the trial, all applicable activities scheduled for the final trial visit should be performed at the time of discontinuation. Any adverse events which are present at the time of discontinuation/withdrawal should be followed in accordance with the safety requirements outlined in Section 7.2 - Assessing and Recording Adverse Events. Please refer to Section 5.8 for trial-specific subject discontinuation criteria.

7.1.4.1.1 Withdrawal From Future Biomedical Research

Subjects may withdraw their consent for Future Biomedical Research and have their specimens and all derivatives destroyed. Subjects may withdraw consent at any time by contacting the principal investigator for the main trial. If medical records for the main trial are still available, the investigator will contact the Sponsor using the designated mailbox (clinical.specimen.management@merck.com), and a form will be provided by the Sponsor to obtain appropriate information to complete specimen withdrawal. Subsequently, the subject's specimens will be removed from the biorepository and be destroyed. A letter will be sent from the Sponsor to the investigator confirming the destruction. It is the

responsibility of the investigator to inform the subject of completion of destruction. Any analyses in progress at the time of request for destruction or already performed prior to the request being received by the Sponsor will continue to be used as part of the overall research trial data and results. No new analyses would be generated after the request is received.

In the event that the medical records for the main trial are no longer available (e.g., if the investigator is no longer required by regulatory authorities to retain the main trial records) or the specimens have been completely anonymized, there will no longer be a link between the subject's personal information and their specimens. In this situation, the request for specimen destruction cannot be processed.

7.1.4.2 Blinding/Unblinding

When the investigator or sub-investigator needs to identify the drug used by a subject and the dosage administered in case of emergency e.g., the occurrence of serious adverse experiences, he/she will contact the emergency unblinding call center by telephone and make a request for emergency unblinding. As requested by the investigator or sub-investigator, the emergency unblinding call center will provide the information to him/her promptly and report unblinding to the Sponsor. The emergency unblinding call center will make a record promptly; however, the investigator or sub-investigator must enter the intensity of the adverse experiences observed, their relation to study drug, the reason thereof, etc., in the medical chart etc., before unblinding is performed.

Additionally, the investigator must go into the IVRS system and perform the unblind in the IVRS system to update drug disposition. In the event that the emergency unblinding call center is not available for a given site in this trial, IVRS/IWRS should be used for emergency unblinding in the event that this is required for subject safety.

In the event that unblinding has occurred, the circumstances around the unblinding (e.g., date and reason) must be documented promptly, and the Sponsor Clinical Director notified as soon as possible. Only the principal investigator, or delegate and the respective subject's code should be unblinded. Trial site personnel and Sponsor directly associated with the conduct of the trial should not be unblinded.

At the end of the trial, random code/disclosure envelopes or lists and unblinding logs are to be returned to the Sponsor or designee.

7.1.4.3 Calibration of Equipment

The investigator or qualified designee has the responsibility to ensure that any device or instrument used for a clinical evaluation/test during a clinical study that provides information about inclusion/exclusion criteria and/or safety or efficacy parameters shall be suitably calibrated and/or maintained to ensure that the data obtained is reliable and/or reproducible. Documentation of equipment calibration must be retained as source documentation at the study site.

7.1.5 Visit Requirements

Visit requirements are outlined in Section 6.0 - Trial Flow Chart. Specific procedure-related details are provided above in Section 7.1 - Trial Procedures.

7.1.5.1 Screening

Early Consent Option

Potential subjects enrolled under the early consent option will be evaluated against all inclusion and exclusion criteria through a 2-step process within a 2-week timeframe. The first step, at the time of early consent, entails evaluation against a subset of inclusion criteria (Inclusion Criteria #1, #7, and #9), as described in Section 6.0 (Trial Flow Chart). If these 3 early screening criteria are fulfilled and the subject goes on to develop signs and symptoms of pneumonia, the second step of evaluations will occur. The subject will be evaluated against all remaining inclusion criteria and all exclusion criteria as described in Section 5.1; if all remaining entry requirements are fulfilled, the subject will be randomized within 48 hours. Subjects who meet the early screening criteria but do not go on to meet the remaining study entry criteria will be considered screen failures.

Standard Consent Option

Within 48 hours prior to randomization, potential subjects will be evaluated to determine that they fulfill the entry requirements as set forth in Section 5.1.

Rescreening of an individual subject for enrollment is not expected to occur commonly. In Screening procedures may be repeated in some circumstances but only after consultation with the Sponsor.

7.1.5.2 Treatment Period

IV study therapy (with either IMI/REL or PIP/TAZ) will be administered in a blinded fashion. IV study therapy should be administered for a minimum of 7 full days to up to a maximum of 14 days. Subjects with evidence of concurrent bacteremia of with *P. aeruginosa* infections should receive 14 days of IV study therapy.

Since the study will enroll subjects without confirmed microbiological (culture) evidence of the HABP/VABP pathogen from a lower respiratory tract specimen at study entry, the use of initial empiric treatment with IV open-label linezolid (600 mg q12h) for methicillin-resistant *S. aureus* (MRSA) infection is required. The recommended treatment duration for a confirmed MRSA infection is a minimum of 7 days, with a minimum of 14 days for MRSA bacteremia. Empiric linezolid treatment will be stopped if the final culture results from the baseline lower respiratory tract sample do not demonstrate the presence of MRSA.

For subjects who receive 7 to 9 days of IV study therapy, Day 10 (OTX3 [Visit 5]) will not be completed. Assessments and procedures while on IV study therapy will be completed at the indicated times and intervals as per the Flow Chart in Section 6.0. The EOT visit for subjects who receive 7 to 9 days of therapy will be completed within 24 hours after the last dose of IV study therapy on Day 7, Day 8 or Day 9 followed by the subsequent post-treatment visits as indicated on the Flow Chart.

Subjects receiving IV study therapy through Day 10 and up to Day 14 will complete all OTX visits (OTX1 on Day 3, OTX2 on Day 6, and OTX3 on Day 10). Assessments and procedures while on IV study therapy will be completed at the indicated times and intervals as per the Flow Chart in Section 6.0. The EOT visit will be completed within 24 hours after the last dose of IV study therapy (i.e., on Day 10, 11 … Day 14) followed by the subsequent

post-treatment visits as indicated on the Flow Chart in Section 6.0. Subjects who end treatment on Day 10 will have a combined OTX3 and EOT visit. All procedures required for each visit must be completed for the combined visit. Procedures that are common to both visits would only be assessed once.

7.1.5.3 Post-Therapy

An EFU visit (7 to 14 days after the cessation of IV study therapy [i.e., 7 to 14 days after EOT]) and a Day 28 post-randomization visit must be completed for each subject. Depending on the total duration of IV study therapy, the EFU and Day 28 post-randomization visit may be combined into a single visit. These visits may only be combined as long as compliance with the protocol-specified visit windows is maintained for both visits.

Specifically, if a subject receives 12 to 14 days of IV study therapy, the EFU visit may potentially be combined with the Day 28 post-randomization visit. For example, if 12 days of IV study therapy are provided, the EFU visit could be scheduled 16 days (14+2) following completion of therapy which would be 28 days (12 days of IV therapy +16 days of follow-up) following randomization. It is required that for any case, the allowable visit window ranges are maintained and all procedures required for each visit must be completed for the combined visit.

7.2 Assessing and Recording Adverse Events

An adverse event is defined as any untoward medical occurrence in a patient or clinical investigation subject administered a pharmaceutical product and which does not necessarily have to have a causal relationship with this treatment. An adverse event can therefore be any unfavourable and unintended sign (including an abnormal laboratory finding, for example), symptom, or disease temporally associated with the use of a medicinal product or protocol-specified procedure, whether or not considered related to the medicinal product or protocol-specified procedure. Any worsening (i.e., any clinically significant adverse change in frequency and/or intensity) of a preexisting condition that is temporally associated with the use of the Sponsor's product, is also an adverse event.

Changes resulting from normal growth and development that do not vary significantly in frequency or severity from expected levels are not to be considered adverse events. Examples of this may include, but are not limited to, teething, typical crying in infants and children and onset of menses or menopause occurring at a physiologically appropriate time.

Sponsor's product includes any pharmaceutical product, biological product, device, diagnostic agent or protocol-specified procedure, whether investigational (including placebo or active comparator medication) or marketed, manufactured by, licensed by, provided by or distributed by the Sponsor for human use.

Adverse events may occur during clinical trials, or as prescribed in clinical practice, from overdose (whether accidental or intentional), from abuse and from withdrawal.

All adverse events that occur after the consent form is signed but before treatment allocation/randomization must be reported by the investigator if they cause the subject to be excluded from the trial, or are the result of a protocol-specified intervention, including but not limited to washout or discontinuation of usual therapy, diet, placebo treatment or a procedure. From the time of treatment allocation/randomization through 14 days following cessation of treatment, all adverse events must be reported by the investigator. Such events will be recorded at each examination on the Adverse Event case report forms/worksheets. The reporting timeframe for adverse events meeting any serious criteria is described in section 7.2.3.1. The investigator will make every attempt to follow all subjects with non-serious adverse events for outcome.

Electronic reporting procedures can be found in the EDC data entry guidelines. Paper reporting procedures can be found in the Investigator Trial File Binder (or equivalent).

7.2.1 Definition of an Overdose for This Protocol and Reporting of Overdose to the Sponsor

In this trial, an overdose in IV study therapy is defined as (1) administration of a total daily dose of IMI/REL in excess of 4 g (IMI) or 2 g (REL) OR (2) administration of a total daily dose of PIP/TAZ greater than 16g (PIP) and 2g (TAZ)..

If an adverse event(s) is associated with ("results from") the overdose of Sponsor's product or vaccine, the adverse event(s) is reported as a serious adverse event, even if no other seriousness criteria are met.

If a dose of Sponsor's product or vaccine meeting the protocol definition of overdose is taken without any associated clinical symptoms or abnormal laboratory results, the overdose is reported as a non-serious Event of Clinical Interest (ECI), using the terminology "accidental or intentional overdose without adverse effect."

All reports of overdose with and without an adverse event must be reported by the investigator within 24 hours to the Sponsor either by electronic media or paper. Electronic reporting procedures can be found in the EDC data entry guidelines. Paper reporting procedures can be found in the Investigator Trial File Binder (or equivalent).

7.2.2 Reporting of Pregnancy and Lactation to the Sponsor

Although pregnancy and lactation are not considered adverse events, it is the responsibility of investigators or their designees to report any pregnancy or lactation in a subject (spontaneously reported to them) that occurs during the trial.

Pregnancies and lactations that occur after the consent form is signed but before treatment allocation/randomization must be reported by the investigator if they cause the subject to be excluded from the trial, or are the result of a protocol-specified intervention, including but not limited to washout or discontinuation of usual therapy, diet, placebo treatment or a procedure. Pregnancies and lactations that occur from the time of treatment allocation/randomization through 14 days following cessation of Sponsor's product must be reported by the investigator. All reported pregnancies must be followed to the completion/termination of the pregnancy. Pregnancy outcomes of spontaneous abortion, missed abortion, benign hydatidiform mole, blighted ovum, fetal death, intrauterine death,

miscarriage and stillbirth must be reported as serious events (Important Medical Events). If the pregnancy continues to term, the outcome (health of infant) must also be reported.

Such events must be reported within 24 hours to the Sponsor either by electronic media or paper. Electronic reporting procedures can be found in the EDC data entry guidelines. Paper reporting procedures can be found in the Investigator Trial File Binder (or equivalent).

7.2.3 Immediate Reporting of Adverse EventsAdverse Events and Incidents to the Sponsor

7.2.3.1 Serious Adverse EventsAdverse Events and Incidents

serious adverse event is any adverse event occurring at any dose or during any use of Sponsor's product that:

- Results in death;
- Is life threatening;
- Results in persistent or significant disability/incapacity;
- Results in or prolongs an existing inpatient hospitalization;
- Is a congenital anomaly/birth defect;
- Is an other important medical event.

Note: In addition to the above criteria, adverse events meeting either of the below criteria, although not serious per ICH definition, are reportable to the Sponsor in the same timeframe as SAEs to meet certain local requirements. Therefore, these events are considered serious by the Sponsor for collection purposes.

- Is a cancer;
- Is associated with an overdose.

Refer to Table 12 for additional details regarding each of the above criteria.

For the time period beginning when the consent form is signed until treatment allocation/randomization, any serious adverse event, or follow up to a serious adverse event, including death due to any cause, that occurs to any subject must be reported within 24 hours to the Sponsor if it causes the subject to be excluded from the trial, or is the result of a protocol-specified intervention, including but not limited to washout or discontinuation of usual therapy, diet, placebo treatment or a procedure.

For the time period beginning at treatment allocation/randomization through 14 days following cessation of treatment, any serious adverse event, or follow up to a serious adverse event, including death due to any cause, whether or not related to the Sponsor's product, must be reported within 24 hours to the Sponsor either by electronic media or paper. Electronic reporting procedures can be found in the EDC data entry guidelines. Paper reporting procedures can be found in the Investigator Trial File Binder (or equivalent).

Additionally, any serious adverse event, considered by an investigator who is a qualified physician to be related to the Sponsor's product that is brought to the attention of the investigator at any time outside of the time period specified in the previous paragraph also must be reported immediately to the Sponsor.

All subjects with serious adverse events must be followed up for outcome.

7.2.3.2 Events of Clinical Interest

Selected non-serious and serious adverse events are also known as Events of Clinical Interest (ECI) and must be reported to the Sponsor.

For the time period beginning when the consent form is signed until treatment allocation/randomization, any ECI, or follow up to an ECI, that occurs to any subject must be reported within 24 hours to the Sponsor if it causes the subject to be excluded from the trial, or is the result of a protocol-specified intervention, including but not limited to washout or discontinuation of usual therapy, diet, placebo treatment or a procedure.

For the time period beginning at treatment allocation/randomization through 14 days following cessation of treatment, any ECI, or follow up to an ECI, whether or not related to the Sponsor's product, must be reported within 24 hours to the Sponsor, either by electronic media or paper. Electronic reporting procedures can be found in the EDC data entry guidelines. Paper reporting procedures can be found in the Investigator Trial File Binder (or equivalent).

Events of clinical interest for this trial include:

1. an overdose of Sponsor's product, as defined in Section 7.2.1 - Definition of an Overdose for This Protocol and Reporting of Overdose to the Sponsor, that is not associated with clinical symptoms or abnormal laboratory results.
2. post baseline laboratory test values that meet the following criteria: an elevated AST or ALT lab value that is greater than or equal to 3X the upper limit of normal and an elevated total bilirubin lab value that is greater than or equal to 2X the upper limit of normal and, at the same time, an alkaline phosphatase lab value that is less than 2X the upper limit of normal, as determined by way of protocol-specified laboratory testing or unscheduled laboratory testing.*

   <u>*Note:</u> These criteria are based upon available regulatory guidance documents. The purpose of the criteria is to specify a threshold of abnormal hepatic tests that may require an additional evaluation for an underlying etiology. The trial site guidance for assessment and follow up of these criteria can be found in the Investigator Trial File Binder (or equivalent).
3. a confirmed (i.e., verified by repeat testing) elevated AST or ALT laboratory value that is greater than or equal to 5 X ULN as a result of within-protocol-specific testing or unscheduled testing.

   <u>NOTE</u>: In subjects with pre-existing elevations in transaminase values, only a further elevation that is not anticipated from an underlying medical condition will be considered an ECI. These events may require an additional evaluation for an underlying etiology. The trial site guidance for assessment and follow up of these criteria can be found in the Investigator Trial File Binder.

7.2.4 Evaluating Adverse Events

An investigator who is a qualified physician will evaluate all adverse events with respect to the elements outlined in Table 12. The investigator's assessment of causality is required for each adverse event. Refer to Table 12 for instructions in evaluating adverse events.

Table 12 Evaluating Adverse Events

**Maximum Intensity**	**Mild**	awareness of sign or symptom, but easily tolerated (for pediatric trials, awareness of symptom, but easily tolerated)
	**Moderate**	discomfort enough to cause interference with usual activity (for pediatric trials, definitely acting like something is wrong)
	**Severe**	incapacitating with inability to work or do usual activity (for pediatric trials, extremely distressed or unable to do usual activities)
**Seriousness**	A serious adverse event (AE) is any adverse event occurring at any dose or during any use of Sponsor's product that:	
	†**Results in death**; or	
	†**Is life threatening; or** places the subject, in the view of the investigator, at immediate risk of death from the event as it occurred [Note: This does not include an adverse event that, had it occurred in a more severe form, might have caused death.]; or	
	†**Results in a persistent or significant disability/incapacity** (substantial disruption of one's ability to conduct normal life functions); or	
	†**Results in or prolongs an existing inpatient hospitalization** (hospitalization is defined as an inpatient admission, regardless of length of stay, even if the hospitalization is a precautionary measure for continued observation. (Note: Hospitalization for an elective procedure to treat a pre-existing condition that has not worsened is not a serious adverse event. A pre-existing condition is a clinical condition that is diagnosed prior to the use of a Merck product and is documented in the patient's medical history.); or	
	†**Is a congenital anomaly/birth defect (**in offspring of subject taking the product regardless of time to diagnosis); or	
	**Is a cancer** (although not serious per ICH definition, is reportable to the Sponsor within 24 hours to meet certain local requirements); **or**	
	**Is associated with an overdose** (whether accidental or intentional). Any adverse event associated with an overdose is considered a serious adverse event for collection purposes. An overdose that is not associated with an adverse event is considered a non-serious event of clinical interest and must be reported within 24 hours.	
	**Other important medical events** that may not result in death, not be life threatening, or not require hospitalization may be considered a serious adverse event when, based upon appropriate medical judgment, the event may jeopardize the subject and may require medical or surgical intervention to prevent one of the outcomes listed previously (designated above by a †).	
**Duration**	Record the start and stop dates of the adverse event. If less than 1 day, indicate the appropriate length of time and units	
**Action taken**	Did the adverse event cause the Sponsor's product to be discontinued?	
**Relationship to Sponsor's Product**	Did the Sponsor's product cause the adverse event? The determination of the likelihood that the Sponsor's product caused the adverse event will be provided by an investigator who is a qualified physician. The investigator's signed/dated initials on the source document or worksheet that supports the causality noted on the AE form, ensures that a medically qualified assessment of causality was done. This initialed document must be retained for the required regulatory time frame. The criteria below are intended as reference guidelines to assist the investigator in assessing the likelihood of a relationship between the test drug and the adverse event based upon the available information<br>**The following components are to be used to assess the relationship between the Sponsor's product and the AE**; the greater the correlation with the components and their respective elements (in number and/or intensity), the more likely the Sponsor's product caused the adverse event:	
	**Exposure**	Is there evidence that the subject was actually exposed to the Sponsor's product such as: reliable history, acceptable compliance assessment (pill count, diary, etc.), expected pharmacologic effect, or measurement of drug/metabolite in bodily specimen?
	**Time Course**	Did the AE follow in a reasonable temporal sequence from administration of the Sponsor's product?<br>Is the time of onset of the AE compatible with a drug-induced effect (applies to trials with investigational medicinal product)?
	**Likely Cause**	Is the AE not reasonably explained by another etiology such as underlying disease, other drug(s)/vaccine(s), or other host or environmental factors

<table>
<tr><td rowspan="3">Relationship to Sponsor's Product (continued)</td><td colspan="2">The following components are to be used to assess the relationship between the Sponsor's product and the AE: (continued)</td></tr>
<tr><td>Dechallenge</td><td>Was the Sponsor's product discontinued or dose/exposure/frequency reduced?
If yes, did the AE resolve or improve?
If yes, this is a positive dechallenge. If no, this is a negative dechallenge.
(Note: This criterion is not applicable if: (1) the AE resulted in death or permanent disability; (2) the AE resolved/improved despite continuation of the Sponsor's product; (3) the trial is a single-dose drug trial); or (4) Sponsor's product(s) is/are only used one time.)</td></tr>
<tr><td>Rechallenge</td><td>Was the subject re-exposed to the Sponsor's product in this trial?
If yes, did the AE recur or worsen?
If yes, this is a positive rechallenge. If no, this is a negative rechallenge.
(Note: This criterion is not applicable if: (1) the initial AE resulted in death or permanent disability, or (2) the trial is a single-dose drug trial); or (3) Sponsor's product(s) is/are used only one time.)
NOTE: IF A RECHALLENGE IS PLANNED FOR AN ADVERSE EVENT WHICH WAS SERIOUS AND WHICH MAY HAVE BEEN CAUSED BY THE SPONSOR'S PRODUCT, OR IF RE-EXPOSURE TO THE SPONSOR'S PRODUCT POSES ADDITIONAL POTENTIAL SIGNIFICANT RISK TO THE SUBJECT THEN THE RECHALLENGE MUST BE APPROVED IN ADVANCE BY THE SPONSOR CLINICAL DIRECTOR AND THE INSTITUTIONAL REVIEW BOARD/INDEPENDENT ETHICS COMMITTEE.</td></tr>
<tr><td></td><td>Consistency with Trial Treatment Profile</td><td>Is the clinical/pathological presentation of the AE consistent with previous knowledge regarding the Sponsor's product or drug class pharmacology or toxicology?</td></tr>
<tr><td colspan="3">The assessment of relationship will be reported on the case report forms /worksheets by an investigator who is a qualified physician according to his/her best clinical judgment, including consideration of the above elements.</td></tr>
<tr><td colspan="2">Record one of the following:</td><td>Use the following scale of criteria as guidance (not all criteria must be present to be indicative of a Sponsor's product relationship).</td></tr>
<tr><td colspan="2">Yes, there is a reasonable possibility of Sponsor's product relationship.</td><td>There is evidence of exposure to the Sponsor's product. The temporal sequence of the AE onset relative to the administration of the Sponsor's product is reasonable. The AE is more likely explained by the Sponsor's product than by another cause.</td></tr>
<tr><td colspan="2">No, there is not a reasonable possibility of Sponsor's product relationship</td><td>Subject did not receive the Sponsor's product OR temporal sequence of the AE onset relative to administration of the Sponsor's product is not reasonable OR the AE is more likely explained by another cause than the Sponsor’s product. (Also entered for a subject with overdose without an associated AE.)</td></tr>
</table>

7.2.5 Sponsor Responsibility for Reporting Adverse Events

All Adverse Events will be reported to regulatory authorities, IRB/IECs and investigators in accordance with all applicable global laws and regulations, i.e., per ICH Topic E6 (R1) Guidelines for Good Clinical Practice.

7.3 TRIAL GOVERNANCE AND OVERSIGHT

7.3.1 Scientific Advisory Committee

This trial was developed in collaboration with a Scientific Advisory Committee (SAC). The SAC comprises both Sponsor and non-Sponsor scientific experts who provide input with respect to trial design, interpretation of trial results and subsequent peer-reviewed scientific publications.

7.3.2 Executive Oversight Committee

The Executive Oversight Committee (EOC) comprises members of Sponsor Senior Management. The EOC will receive and decide upon any recommendations made by the external Data Monitoring Committee (eDMC) regarding the trial.

7.3.3 Data Monitoring Committee

To supplement the routine trial monitoring outlined in this protocol, an external Data Monitoring Committee (DMC) will monitor the interim data from this trial. The voting members of the committee are external to the Sponsor. The members of the DMC must not be involved with the trial in any other way (e.g., they cannot be trial investigators) and must have no competing interests that could affect their roles with respect to the trial.

The DMC will make recommendations to the EOC regarding steps to ensure both subject safety and the continued ethical integrity of the trial. Also, the DMC will review interim trial results, consider the overall risk and benefit to trial participants (see Section 8.2 - Interim Analyses) and recommend to the EOC if the trial should continue in accordance with the protocol.

Specific details regarding composition, responsibilities, and governance, including the roles and responsibilities of the various members and the Sponsor protocol team; meeting facilitation; the trial governance structure; and requirements for and proper documentation of DMC reports, minutes, and recommendations will be described in a separate charter that is reviewed and approved by the DMC. The DMC will monitor the trial at an appropriate frequency, as described in the detailed DMC charter. The DMC will also make recommendations to the Sponsor protocol team regarding steps to ensure both subject safety and the continued ethical integrity of the trial.

A DMC recommendation will be communicated to the Sponsor as agreed to in the Collaboration agreement.

8.0 STATISTICAL ANALYSIS PLAN

This section outlines the statistical analysis strategy and procedures for the study. If, after the study has begun, but prior to any unblinding/final database lock, changes are made to primary and/or key secondary hypotheses, or the statistical methods related to those hypotheses, then the protocol will be amended (consistent with ICH Guideline E-9). Changes to exploratory or other non-confirmatory analyses made after the protocol has been finalized, but prior to unblinding/final database lock, will be documented in a supplemental SAP (sSAP) and referenced in the Clinical Study Report (CSR) for the study. In addition, a separate EU-SAP will be prepared for this protocol specifically to document a revised ordering of the primary and secondary objectives from what is outlined in Section 3.0. This revised ordering of the primary and secondary objectives is designed to be consistent with the 2014 CHMP Addendum to the Guideline on the Evaluation of Medicinal Products Indicated for Treatment of Bacterial Infections as well as guidance received during the CHMP Scientific Advice Oral Hearing for MK-7655A held in February 2015. The analyses documented in the protocol SAP and the EU SAP will be executed simultaneously following the final database lock. The analyses described in the protocol SAP will be used to support U.S. FDA review (and other regions of the world); the analyses described in the EU-SAP will be used to support EU review. As such, issues of multiplicity and Type I error inflation do not arise [22]. The initial publication of the trial results will follow the ordering of the primary and secondary objectives that is outlined in Section 3.0. Post hoc exploratory analyses will be clearly identified in the CSR.

8.1 Statistical Analysis Plan Summary

Key elements of the statistical analysis plan are summarized below; the comprehensive plan is provided in Sections 8.0-8.12.

**Study Design Overview**	A randomized, double-blind (with in-house blinding), active-controlled, parallel-group, multi-site, trial of imipenem/cilastatin/relebactam (also known as MK-7655A; hereafter referred to as IMI/REL) compared with piperacillin/tazobactam (PIP/TAZ) in subjects with hospital-acquired bacterial pneumonia (HABP) or ventilator-associated bacterial pneumonia (VABP).
**Treatment Assignment**	This study will randomize subjects in a 1:1 ratio to the two treatment arms of the study (Group 1: IMI/REL and Group 2: PIP/TAZ). Randomization will be stratified by: 1) pneumonia type at baseline (non-ventilated HABP vs. ventilated HABP/VABP) and 2) APACHE II score at baseline (< 15 vs. ≥ 15).<br><br>A double-blind/masking technique will be used. IMI/REL and PIP/TAZ will be dispensed in a blinded fashion by an unblinded pharmacist. Due to a visual difference in the appearance of study treatment solutions, the infusion bags will be covered with an opaque sleeve by the unblinded pharmacist to ensure that other study personnel and all subjects remain blinded to clinical material assignments. The personnel involved in the administration of the study infusion treatment should be unaware of the treatment group assignments. The subject, the investigator and Sponsor personnel or delegate(s) who are involved in the treatment or clinical evaluation of the subjects are unaware of the group assignments.

**Analysis Populations**	Primary Efficacy Analysis: modified intention-to-treat (MITT) population (defined as all randomized subjects who receive at least one dose of IV study therapy and do not have the presence of Gram positive cocci **only** on baseline Gram stain).<br><br>Safety: All Subjects as Treated (ASaT)
**Primary Endpoint(s)**	Incidence of all-cause mortality through Day 28 post-randomization
**Key Secondary Endpoints**	Percentage of subjects achieving a favorable clinical response at EFU
**Statistical Methods for Key Efficacy/Immunogenicity/ Pharmacokinetic Analyses**	For the primary hypothesis (mortality), IMI/REL will be compared to PIP/TAZ using the stratified Miettinen and Nurminen method. IMI/REL will be considered non-inferior (NI) to PIP/TAZ if the upper bound of the 2-sided 95% confidence interval for the difference in incidence between the treatment groups (IMI/REL minus PIP/TAZ) is less than 10%. If non-inferiority is established, then superiority of IMI/REL compared to PIP/TAZ will be evaluated.<br><br>For the key secondary hypothesis (clinical response), IMI/REL will be compared to PIP/TAZ using the stratified Miettinen and Nurminen method. IMI/REL will be considered non-inferior (NI) to PIP/TAZ if the lower bound of the 2-sided 95% confidence interval (CI) for the difference in percentages between the treatment groups (IMI/REL minus PIP/TAZ) is greater than -12.5%. If non-inferiority is established, then superiority of IMI/REL compared to PIP/TAZ will be evaluated.
**Statistical Methods for Key Safety Analyses**	P-values (Tier 1 only) and 95% confidence intervals (Tier 1 and Tier 2) will be provided for between-treatment differences in the percentage of subjects with events; these analyses will be performed using the Miettinen and Nurminen method
**Interim Analyses**	A blinded review of the aggregate mortality rate will be ongoing during the study. The impact of the blinded aggregate rate on the assumptions underlying the power/sample size calculation will be formally assessed by the Sponsor when approximately 75% of the planned sample size (N=402) have completed 28 days of follow up. If Day 28 all-cause mortality is higher than the 15% assumed in the power calculation, consideration will be given to increasing the sample size.<br><br>There are no plans to conduct a formal interim analysis of unblinded efficacy data in the study.
**Multiplicity**	A sequential testing approach will be employed to strongly control Type 1 error across primary and key secondary endpoints.
**Sample Size and Power**	The planned sample size of 268 subjects per group will provide 90% power to reject the null hypothesis that the true difference in all-cause mortality exceeds the NI margin of 10% assuming true rates for both the control and experimental regimens of 15% (1-tailed alpha of 2.5%). The planned sample size of 268 subjects per group will provide 84% power to reject the null hypothesis that the true difference in favorable clinical response at the EFU visit exceeds the NI margin of 12.5% assuming true rates for both the control and experimental regimens of 60% (1-tailed alpha of 2.5%).

8.2 Responsibility for Analyses/In-House Blinding

The statistical analyses of the data obtained from this study will be the responsibility of the Clinical Biostatistics department of the SPONSOR. Certain specific analyses such as pharmacokinetics and the evaluation of pharmacokinetic-pharmacodynamic association will be the responsibility of the appropriate departments of the SPONSOR.

This study will be conducted as a double-blind study under in-house blinding procedures. The official, final database will not be unblinded until medical/scientific review has been performed, protocol deviations have been identified, and data have been declared final and complete.

Separate functional unblinding for bioanalysis will be conducted in support of pharmacokinetic evaluations. A small team as specified in a separate Modeling and Simulation (M&S) Analysis Plan, and who are separate from the study team, will be unblinded for the purpose of preparing the pharmacokinetic analyses. No interim data or results will be shared with the study team before the primary analyses have been completed, and the unblinded group will not be members of the study team. No decisions will be made based on this functional unblinding that could influence the conduct of the trial.

The Clinical Biostatistics department will generate the randomized allocation schedule(s) for study treatment assignment. Randomization will be implemented using an interactive voice response system (IVRS).

Planned interim analyses are described in Section 8.7. Study enrollment is likely to be ongoing at the time of any interim analyses. Blinding to treatment assignment will be maintained at all investigational sites. The results of interim analyses will not be shared with the investigators prior to the completion of the study. Subject-level unblinding will be restricted to an external unblinded statistician and scientific programmer performing the interim analysis, who will have no other responsibilities associated with the study.

There are no plans to conduct a formal interim analysis of unblinded efficacy data in the study. Periodic reviews of the safety data will be performed by the eDMC. Treatment-level results will be provided by the external unblinded statistician to the external Data Monitoring Committee (eDMC). Limited additional SPONSOR personnel (e.g., members of the EOC) may be unblinded to the treatment level results, if required, in order to act on the recommendations of the eDMC. The extent to which individuals are unblinded with respect to results will be documented by the unblinded statistician.

8.3 Hypotheses/Estimation

Objectives and hypotheses of the study are stated in Section 3.

8.4 Analysis Endpoints

Efficacy and safety endpoints that will be evaluated for within- and/or between-treatment differences are listed below.

8.4.1 Efficacy Endpoints

A full description of the efficacy measures is provided in Section 4.2.3.1. The primary efficacy endpoint is the incidence of all-cause mortality within 28 days after initiation of study therapy. IMI/REL will be considered non-inferior (NI) to PIP/TAZ if the upper bound of the 2-sided 95% confidence interval (CI) for the difference in incidence between the treatment groups (IMI/REL minus PIP/TAZ) is less than 10%. The 10% NI margin is selected to be consistent with guidance provided in the 2014 FDA Guidance for Industry *Hospital-Acquired Bacterial Pneumonia and Ventilator-Associated Bacterial Pneumonia: Developing Drugs for Treatment* [23]. If non-inferiority is established, then a subsequent test will be performed to determine whether or not IMI/REL is superior to PIP/TAZ (i.e., the upper bound of the 2-sided 97.5% CI for the difference in incidence between the treatment groups is less than 0%). The rationale for the 97.5% CI will be explained in Section 8.8 (Multiplicity). The key secondary endpoint is the percentage of subjects achieving a favorable clinical response at EFU (favorable is defined as an assessment of "cure" or "sustained cure") in the IMI/REL group will be compared to that in the PIP/TAZ group. IMI/REL will be considered non-inferior (NI) to PIP/TAZ if the lower bound of the 2-sided 95% confidence interval (CI) for the difference in percentages between the treatment groups (IMI/REL minus PIP/TAZ) is greater than -12.5%. An NI margin of 12.5% is selected for this endpoint based on the 2014 CHMP *Addendum to the Guideline on the Evaluation of Medicinal Products Indicated for Treatment of Bacterial Infections.* If non-inferiority is established, then a subsequent test will be performed to determine whether or not IMI/REL is superior to PIP/TAZ (i.e., the lower bound of the 2-sided 97.5% CI for the difference in percentages between the treatment groups is greater than 0%). The rationale for the 97.5% CI will be explained in Section 8.8 (Multiplicity).

IV study therapy will be administered for a minimum of 7 days up to a maximum of 14 days (see Section 4.2.2). The duration of therapy will be summarized by treatment group for the overall population. In addition, the impact of any difference in duration of therapy on the treatment group comparisons will be assessed.

8.4.2 Safety Endpoints

A description of safety measures is provided in Section 4.2.3.2. The analysis of safety endpoints will follow a tiered approach. For this protocol, the following are pre-specified events of interest (Tier 1 events).

1. An elevated AST or ALT laboratory value that is greater than or equal to 3 X ULN and an elevated total bilirubin laboratory value that is greater than or equal to 2 X ULN and, at the same time, an alkaline phosphatase laboratory value that is less than 2 X ULN, as a result of within-protocol-specific testing or unscheduled testing. It will not be counted as a Tier 1 event if the preceding conditions were present at randomization (Day 1).
2. A confirmed (i.e., verified by repeat testing) elevated AST or ALT laboratory value that is greater than or equal to 5 X ULN as a result of within-protocol-specific testing or unscheduled testing. It will not be counted as a Tier 1 event if the preceding conditions were present at randomization (Day 1).

The broad clinical and laboratory adverse event (AE) categories, consisting of the percentage of subjects with any AE, a drug-related AE, a serious AE, an AE which is both drug related and serious, who discontinued IV study therapy due to an AE, and who discontinued IV study therapy due to a drug-related AE, will be considered Tier 2 endpoints.

8.5 Analysis Populations

8.5.1 Efficacy Analysis Populations

The modified intention-to-treat (MITT) population will serve as the primary population for efficacy analyses in this study. The MITT population is defined as all randomized subjects who receive at least one dose of IV study therapy and do not have the presence of Gram positive cocci **only** on baseline Gram stain.

The microbiological modified intention-to-treat (mMITT) population is the secondary population for efficacy analyses. The mMITT population is defined as all randomized subjects who receive at least one dose of IV study therapy and do not have Gram positive cocci **only** on baseline Gram stain and who have a baseline bacterial pathogen identified as the cause of HABP/VABP against which IMI/REL has been shown to have antibacterial activity.

The clinically-evaluable (CE) and microbiologically-evaluable (ME) populations will serve as additional analysis populations for the some of the secondary and exploratory efficacy endpoints. The CE population is a subset of the MITT population who also meet the following criteria:

1. Meet important diagnostic criteria for entry into the study,
2. Have no significant deviation from the protocol that could impact the assessment of efficacy,
3. Receive the minimum duration of IV study therapy, and
4. Have an efficacy assessment at the time point of interest.

The CE population does not require a positive baseline culture for a bacterial pathogen identified as the cause of HABP/VABP against which IMI/REL has been shown to have antibacterial activity.

The ME population is a subset of the CE population who also meet the following criteria:

1. Have a baseline bacterial pathogen identified as the cause of HABP/VABP against which IMI/REL has been shown to have antibacterial activity, and
2. Have results from a lower respiratory tract culture obtained at the indicated time point.

The final determination on major protocol deviations, and thereby the composition of the CE and ME populations, will be made prior to the final unblinding of the database and will be documented in a separate memo.

Subjects will be included in the treatment group to which they are randomized for the analysis of efficacy data using both the MITT and mMITT populations. Details on the approach to handling missing data are provided in Section 8.6 (Statistical Methods)

8.5.2 Safety Analysis Populations

The All Subjects as Treated (ASaT) population will be used for the analysis of safety data in this study. The ASaT population consists of all randomized subjects who received at least one dose of IV study therapy. Subjects will be included in the treatment group corresponding to the IV study therapy they actually received for the analysis of safety data using the ASaT population. For most subjects this will be the treatment group to which they are randomized. Subjects who take incorrect study therapy for the entire treatment period will be included in the treatment group corresponding to the study therapy actually received.

At least one laboratory or vital sign measurement obtained subsequent to at least one dose of IV study therapy is required for inclusion in the analysis of each specific parameter. To assess change from baseline, a baseline measurement is also required.

For the analysis of pre-specified events of interest (Tier 1 events) described above in Section 8.4.2, subjects will be excluded from specific analyses if the conditions defining the event were present at randomization (Day 1).

Details on the approach to handling missing data for safety analyses are provided in Section 8.6 (Statistical Methods).

8.6 Statistical Methods

Efficacy results that will be deemed to be statistically significant after consideration of the Type I error control strategy are described in Section 8.8 Multiplicity. Nominal p-values will be computed for other efficacy analyses, but should be interpreted with caution due to potential issues of multiplicity, sample size, etc. Unless otherwise stated, all statistical tests of efficacy endpoints will be conducted at the $\alpha=0.050$ (2-sided) level (equivalent to the $\alpha=0.025$ (1-sided) level often associated with non-inferiority trials).

8.6.1 Statistical Methods for Efficacy Analyses

The analysis of between-treatment differences in the incidence (or percentage of subjects) with binary events will be performed using the Miettinen and Nurminen method (1985) [24], an unconditional, asymptotic method. The analyses will be stratified by: 1) pneumonia type at baseline (non-ventilated HABP vs. ventilated HABP/VABP) and 2) APACHE II score at baseline (< 15 vs. ≥ 15) and Cochran-Mantel-Haenszel (CMH) weights will be used.

Missing Values

Any subject missing an evaluation for a specific endpoint (clinical or microbiological) at any particular visit will be generally considered as being "indeterminate" for that endpoint in the MITT and mMITT populations. The following are exceptions to this rule:

- Subjects discontinuing IV study therapy due to lack of efficacy (i.e., withdrawals with subsequent non-study antibiotic therapy) will be considered as "failures" with respect to clinical response at the time of discontinuation and all subsequent time points.

- Subjects discontinuing IV study therapy due to lack of efficacy will be presumed to have persistence for the microbiological response at the time of discontinuation and all subsequent time points.

The primary and key secondary endpoints, primary analysis population, and statistical methods that will be employed for the efficacy analyses are presented in Table 13. Since a favorable clinical response at EFU requires an assessment of "cure" or "sustained cure", an assessment of "indeterminate" would be considered a failure to achieve a favorable clinical response.

For microbiological response, "Indeterminate - No specimen taken because subject is deemed clinically cured or improved" will be considered "presumed eradication". Since a favorable microbiological response requires an assessment of "eradication" or "presumed eradication", the response "Indeterminate - No specimen taken because subject is deemed clinically cured or improved" will be considered a favorable response, while other types of "indeterminate" response will be considered a failure to achieve a favorable microbiological response.

Table 13 Summary of Analysis Strategy for Primary and Key Secondary Efficacy Endpoints

Endpoint/Variable (Description, Time point)	Statistical Method	Analysis Population	Missing Data Approach[1]
Primary:			
Incidence of all-cause mortality through Day 28 post-randomization. NI comparison of IMI/REL to PIP/TAZ using 10% margin	Stratified M&N[1]	MITT[2]	M=F[3]
Secondary:			
Percentage of subjects achieving a favorable clinical response at EFU. NI comparison of IMI/REL to PIP/TAZ using 12.5% margin	Stratified M&N[1]	MITT[2]	M=F[3]

1 M&N is Miettinen and Nurminen method [24] stratified by pneumonia type at baseline (non-ventilated HABP vs. ventilated HABP/VAB and APACHE II score (< 15 vs. ≥ 15) using CMH weights.
2 MITT population includes all randomized subjects who receive at least one dose of IV study therapy and do not have the presence of Gram positive cocci **only** on baseline Gram stain.
3 M=F is missing=failure

8.6.2 Statistical Methods for Safety Analyses

Safety and tolerability will be assessed by clinical review of all relevant parameters including adverse events (AEs), laboratory tests, and vital signs.

The analysis of safety results will follow a tiered approach as shown in Table 14. The tiers differ with respect to the analyses that will be performed. Safety parameters or adverse events of special interest that are identified a priori constitute "Tier 1" safety endpoints that will be subject to inferential testing for statistical significance with p values and 95% confidence intervals provided for between-group comparisons. Other safety parameters will be considered Tier 2 or Tier 3. Tier 2 parameters will be assessed via point estimates with 95% confidence intervals provided for between-group comparisons; only point estimates by treatment group are provided for Tier 3 safety parameters. P-values (Tier 1 only) and 95%

confidence intervals (Tier 1 and Tier 2) will be provided for between-treatment differences in the percentage of subjects with events; these analyses will be performed using the Miettinen and Nurminen method (1985), an unconditional, asymptotic method. Since the stratification factors (pneumonia type at baseline and APACHE II score at baseline) are not considered to be related to safety endpoints, they will not be included as stratification factors in the safety analyses.

Adverse events (specific terms as well as system organ class terms) and predefined limits of change in laboratory and vital signs parameters that are not pre-specified as Tier-1 endpoints will be classified as belonging to "Tier 2" or "Tier 3", based on the number of events observed. Membership in Tier 2 requires that at least 4 subjects in at least one treatment group exhibit the event; all other adverse events and predefined limits of change will belong to Tier 3.

The threshold of at least 4 events was chosen because the 95% confidence interval for the between-group difference in percent incidence will always include zero when treatment groups of equal size each have less than 4 events and thus would add little to the interpretation of potentially meaningful differences. Because many 95% confidence intervals may be provided without adjustment for multiplicity, the confidence intervals should be regarded as a helpful descriptive measure to be used in review, not a formal method for assessing the statistical significance of the between-group differences in adverse events and predefined limits of change.

Continuous measures such as changes from baseline in laboratory and vital signs parameters that are not pre-specified as Tier 1 endpoints will be considered Tier 3 safety parameters. Summary statistics for baseline, on-treatment, and change from baseline values will be provided by treatment group in table format. In addition, summary statistics for the difference between treatment groups will also be provided.

Table 14 Analysis Strategy for Safety Parameters

Safety Tier	Safety Endpoint†	p-Value	95% CI for Treatment Comparison	Descriptive Statistics
Tier 1	Elevated AST or ALT ≥3 X ULN and elevated total bilirubin ≥2 X ULN and alkaline phosphatase <2 X ULN	X	X	X
	A confirmed elevated AST or ALT ≥5 X ULN	X	X	X
Tier 2	Any AE		X	X
	Any Serious AE		X	X
	Any Drug-Related AE		X	X
	Any Serious and Drug-Related AE		X	X
	Discontinuation due to AE		X	X
	Discontinuation due to Drug-Related AE			
	Specific AEs, SOCs, or PDLCs‡ (incidence ≥4 subjects in at least one of the treatment groups)		X	X
Tier 3	Specific AEs, SOCs or PDLCs‡ (incidence <4 subjects in all of the treatment groups)			X
	Change from Baseline Results (Labs, Vital Signs)			X

† Adverse Event references refer to both Clinical and Laboratory AEs.
‡ Includes only those endpoints not pre-specified as Tier 1 or not already pre-specified as Tier-2 endpoints.
Note: SOC=System Organ Class; PDLC=Pre-Defined Limit of Change; X = results will be provided.

8.6.3 Summaries of Baseline Characteristics, Demographics, and Other Analyses

Demographic and Baseline Characteristics

The comparability of the treatment groups for each relevant characteristic will be assessed by the use of descriptive statistics. No statistical hypothesis tests will be performed on these characteristics. The number and percentage of subjects screened, randomized, and the primary reason for discontinuation will be displayed. Demographic variables, baseline characteristics, primary and secondary diagnoses, and prior and concomitant therapies will be summarized by treatment either using descriptive statistics for continuous or categorical variables, as appropriate.

Population PK Analyses

Based on PK data and PD data obtained within this study, population PK and the relationship between PK and PD will be assessed. The results of these exploratory analyses will be included in a separate report performed by the Sponsor.

8.7 Interim Analyses

There are no plans to conduct a formal interim analysis of unblinded efficacy data in the study. Periodic reviews of the safety and efficacy data will be performed by the eDMC so as to assess the benefit vs. risk, as outlined in Section 7.3.3.

While there is no intention to stop the trial early based on a positive efficacy outcome, it is recognized that the eDMC might consider a recommendation to stop the trial should overwhelming efficacy in favor of either treatment be observed. To provide some guidance for the eDMC in this situation, a 2-sided p-value ≤0.001 is proposed for the test of superiority for the Day 28 all-cause mortality endpoint (stratified Miettinen and Nurminen method). This conservative criterion for the superiority test will have no impact on the overall multiplicity strategy described in Section 8.8.

Additional details will be provided in the detailed eDMC charter. An internal blinded sample size re-estimation will be conducted as described in the following paragraph.

Blinded review of the aggregate Day 28 all-cause mortality rate (referred to as the mortality rate for the remainder of this section) will be ongoing during the study. The impact of the mortality rate on the assumptions underlying the power/sample size calculation will be formally assessed when approximately 75% (N=402) of the planned sample size (N=536) have completed 28 days of follow up. If the observed mortality rate is higher than the 15% assumed in the power calculation, consideration will be given to increasing the overall sample size as outlined in Table 15. If the observed mortality rate is less than 15%, the overall sample size will be maintained at the planned N=536 and power for this endpoint/hypothesis will exceed 90%. The maximum sample size will not exceed N=768 (384 per group) regardless of the observed mortality rate. The accruing database will not be officially locked for this blinded sample size re-estimation; however, all data relating to the assessment of mortality will be cleaned and all queries resolved before the formal assessment of the mortality rate. As this sample size re-estimation will be done in a blinded fashion, there is no impact on type 1 error rates.

Table 15 Sample Size Adjustments based on Interim Blinded Review of Day 28 All-Cause Mortality

Observed Mortality Rate (%) †	Power based on Original Sample Size (N=536)	Revised Sample Size ‡	Percent Increase from Original Sample Size
16%	88.4%	566	5.4%
17%	86.9%	594	10.8%
18%	85.4%	622	15.9%
19%	83.9%	648	20.7%
20%	82.5%	674	25.6%
21%	81.1%	698	30.2%
22%	79.8%	722	34.7%
23%	78.5%	746	39.0%
24%	77.4%	768	43.1%

† This is the aggregate Day 28 all-cause mortality rate expressed as a percent and rounded to the nearest integer value.
‡ Calculated to provide 90% power based on the observed mortality rate

8.8 Multiplicity

A sequential testing approach will be employed to strongly control Type 1 error across the primary efficacy endpoint (incidence of Day 28 all-cause mortality) and the key secondary efficacy endpoint (percentage of subjects achieving a favorable clinical response at EFU). Testing will be performed in the following sequence:

Step 1: Non-inferiority for the Day 28 all-cause mortality endpoint will be evaluated using α=0.050, 2-sided (equivalent to α=0.025, 1-sided often associated with non-inferiority tests). If non-inferiority is met, then Step 2 will be performed.

Step 2: Non-inferiority for the favorable clinical response at EFU endpoint will be evaluated using α=0.050, 2-sided (equivalent to α=0.025, 1-sided often associated with non-inferiority tests). If non-inferiority is met, then Step 3 will be performed.

Step 3: Superiority will be evaluated simultaneously for both endpoints with the alpha divided equally between the two (α=0.025, 2-sided for each).

This multiplicity strategy strongly controls the overall Type I error rate at 0.050 across the primary and key secondary efficacy hypotheses.

8.9 Sample Size and Power Calculations

This study will randomize subjects in a 1:1 ratio to the two treatment arms of the study (Group 1: IMI/REL and Group 2: PIP/TAZ), in order to obtain approximately 536 subjects who meet the criteria for inclusion in the modified intention-to-treat (MITT) population. A sample size of 268 subjects per group will provide 90% power to reject the null hypothesis that the true difference in all-cause mortality exceeds the NI margin of 10% assuming true rates for both the control and experimental regimens of 15% (1-tailed alpha of 2.5%). A sample size of 337 subjects per group would be required to maintain 90% power assuming a 20 percent mortality rate for both groups. The power would fall to 82% with the original sample size of 268 subjects per group assuming a 20 percent mortality rate for both groups.

Another way to assess the precision of a non-inferiority trial is to consider the maximum observed difference that would just meet the criterion for non-inferiority (in this case, an upper bound of the 2-sided 95% CI for the difference in all-cause mortality [IMI/REL minus PIP/TAZ] that is just less than 10%). This maximum observed difference will increase as the observed mortality in the control group decreases. An observed difference of 3.0 percentage points will just meet the criterion for non-inferiority given an observed mortality of 19.8% (53/268) in the control group and 22.8% (61/268) in the experimental group. An observed difference of 4.1 percentage points will just meet the criterion for non-inferiority given an observed mortality of 10.1% (27/268) in the control group and 14.2% (38/268) in the experimental group.

A key secondary endpoint for this trial is to evaluate the efficacy of IMI/REL versus PIP/TAZ with respect to clinical response at the early follow-up visit 7 to 14 days after the end of therapy (EFU). In two publications of HABP/VABP trials, clinical response at a similar time point was reported as 60% and 55% for the PIP/TAZ group and 66% and 52% for the IMI group. Assuming true rates for the control and experimental regimens of 60%, a sample size of 268 subjects per group will provide 84% power to reject the null hypothesis

that the true difference in clinical response at the EFU visit exceeds the NI margin of 12.5% (1-tailed alpha of 2.5%). [12] [13]

8.10 Subgroup Analyses and Effect of Baseline Factors

To determine whether the treatment effect is consistent across various subgroups, the estimate of the between-group treatment effect (with a nominal 95% CI) for the Day 28 all-cause mortality and favorable clinical response at EFU endpoints (MITT population) will be estimated within each category of the following classification variables (assessed prior to or at the point of randomization) if there are at least 25 patients in each subgroup in each treatment group:

- Age category (<65, ≥65 years)
- Gender (female, male)
- Race (white, non-white)
- Stratification variable: non-ventilated HABP, ventilated HABP/VABP
- Pneumonia type at baseline (non-ventilated HABP, ventilated HABP, VABP)
- Stratification variable: APACHE II score at baseline (< 15, ≥ 15)
- Region (US, Ex-US)
- CPIS score: (<6 , ≥6)
- Concurrent bacteremia (Y, N)

9.0 LABELING, PACKAGING, STORAGE AND RETURN OF CLINICAL SUPPLIES

9.1 Investigational Product

The investigator shall take responsibility for and shall take all steps to maintain appropriate records and ensure appropriate supply, storage, handling, distribution and usage of investigational product in accordance with the protocol and any applicable laws and regulations.

Clinical Supplies will be provided by the Sponsor as summarized in Table 16.

Table 16 Product Descriptions

Product Name & Potency	Dosage Form
MK-7655A, MK-7655 250 mg / Imipenem 500 mg / Cilastatin 500 mg	Powder for Constitution
Piperacillin / Tazobactam 4g / 0.5g	Powder for Reconstitution

All other supplies not indicated in Table 16 above will be provided centrally by the Sponsor or locally by the trial site, subsidiary or designee, depending on local country operational or regulatory requirements.

For any commercially available product that is provided by the trial site, subsidiary or designee every attempt will be made to source these supplies from a single lot/batch number. The trial site will be responsible for recording the lot number, manufacturer and expiry date of any locally purchased product.

9.2 Packaging and Labeling Information

Clinical supplies will be affixed with a clinical label in accordance with regulatory requirements.

Open label, single-dose vials will be supplied to the unblinded individual(s) at the clinical site. No kitting is required.

9.3 Clinical Supplies Disclosure

This trial is blinded but provided open label; therefore, an unblinded pharmacist or qualified trial site personnel will be used to blind supplies. IV study therapy identity (name, strength or potency) is included in the label text and lists are provided.

The emergency unblinding call center will use the randomization schedule for the trial to unblind subjects and to unmask identity.

Treatment identification information is to be unmasked ONLY if necessary for the welfare of the subject. Every effort should be made not to unblind the subject unless necessary.

In the event that unblinding has occurred, the circumstances around the unblinding (e.g., date and reason) must be documented promptly, and the Sponsor Clinical Director notified as soon as possible. Only the principal investigator or delegate and the respective subject's code should be unblinded. Trial site personnel and Sponsor personnel directly associated with the conduct of the trial should not be unblinded.

9.4 Storage and Handling Requirements

Clinical supplies must be stored in a secure, limited-access location under the storage conditions specified on the label.

Receipt and dispensing of trial medication must be recorded by an authorized person at the trial site.

Clinical supplies may not be used for any purpose other than that stated in the protocol.

9.5 Discard/Destruction/Returns and Reconciliation

The investigator is responsible for keeping accurate records of the clinical supplies received from the Sponsor or designee, the amount dispensed to and returned by the subjects and the amount remaining at the conclusion of the trial. For all trial sites, the local country Sponsor personnel or designee will provide appropriate documentation that must be completed for drug accountability and return, or local discard and destruction if appropriate. Where local

discard and destruction is appropriate, the investigator is responsible for ensuring that a local discard/destruction procedure is documented.

9.6 Standard Policies

Trial site personnel will have access to a central electronic randomization system (IVRS/IWRS system) to allocate subjects, to assign IV study therapy to subjects and to manage the distribution of clinical supplies. Each person accessing the IVRS system must be assigned an individual unique PIN. They must use only their assigned PIN to access the system, and they must not share their assigned PIN with anyone.

10.0 ADMINISTRATIVE AND REGULATORY DETAILS

10.1 Confidentiality

10.1.1 Confidentiality of Data

By signing this protocol, the investigator affirms to the Sponsor that information furnished to the investigator by the Sponsor will be maintained in confidence, and such information will be divulged to the institutional review board, ethics review committee (IRB/ERC) or similar or expert committee; affiliated institution and employees, only under an appropriate understanding of confidentiality with such board or committee, affiliated institution and employees. Data generated by this trial will be considered confidential by the investigator, except to the extent that it is included in a publication as provided in the Publications section of this protocol.

10.1.2 Confidentiality of Subject Records

By signing this protocol, the investigator agrees that the Sponsor (or Sponsor representative), IRB/ERC, or regulatory authority representatives may consult and/or copy trial documents in order to verify worksheet/case report form data. By signing the consent form, the subject agrees to this process. If trial documents will be photocopied during the process of verifying worksheet/case report form information, the subject will be identified by unique code only; full names/initials will be masked prior to transmission to the Sponsor.

By signing this protocol, the investigator agrees to treat all subject data used and disclosed in connection with this trial in accordance with all applicable privacy laws, rules and regulations.

10.1.3 Confidentiality of Investigator Information

By signing this protocol, the investigator recognizes that certain personal identifying information with respect to the investigator, and all subinvestigators and trial site personnel, may be used and disclosed for trial management purposes, as part of a regulatory submissions, and as required by law. This information may include:

1. name, address, telephone number and e-mail address;
2. hospital or clinic address and telephone number;
3. curriculum vitae or other summary of qualifications and credentials; and

4. other professional documentation.

Consistent with the purposes described above, this information may be transmitted to the Sponsor, and subsidiaries, affiliates and agents of the Sponsor, in your country and other countries, including countries that do not have laws protecting such information. Additionally, the investigator's name and business contact information may be included when reporting certain serious adverse events to regulatory authorities or to other investigators. By signing this protocol, the investigator expressly consents to these uses and disclosures.

If this is a multicenter trial, in order to facilitate contact between investigators, the Sponsor may share an investigator's name and contact information with other participating investigators upon request.

10.1.4 Confidentiality of IRB/IEC Information

The Sponsor is required to record the name and address of each IRB/IEC member that reviews and approves this trial. The Sponsor is also required to document that each IRB/IEC meets regulatory and ICH GCP requirements by requesting and maintaining records of the names and qualifications of the IRB/IEC members and to make these records available for regulatory agency review upon request by those agencies.

10.2 Compliance with Financial Disclosure Requirements

Financial Disclosure requirements are outlined in the US Food and Drug Administration Regulations, Financial Disclosure by Clinical Investigators (21 CFR Part 54). It is the Sponsor's responsibility to determine, based on these regulations, whether a request for Financial Disclosure information is required. It is the investigator's/subinvestigator's responsibility to comply with any such request.

The investigator/subinvestigator(s) agree, if requested by the Sponsor in accordance with 21 CFR Part 54, to provide his/her financial interests in and/or arrangements with the Sponsor to allow for the submission of complete and accurate certification and disclosure statements. The investigator/subinvestigator(s) further agree to provide this information on a Certification/Disclosure Form, commonly known as a financial disclosure form, provided by the Sponsor or through a secure password-protected electronic portal provided by the Sponsor. The investigator/subinvestigator(s) also consent to the transmission of this information to the Sponsor in the United States for these purposes. This may involve the transmission of information to countries that do not have laws protecting personal data.

10.3 Compliance with Law, Audit and Debarment

By signing this protocol, the investigator agrees to conduct the trial in an efficient and diligent manner and in conformance with this protocol; generally accepted standards of Good Clinical Practice (e.g., International Conference on Harmonization of Technical Requirements for Registration of Pharmaceuticals for Human Use Good Clinical Practice: Consolidated Guideline and other generally accepted standards of good clinical practice); and all applicable federal, state and local laws, rules and regulations relating to the conduct of the clinical trial.

The Code of Conduct, a collection of goals and considerations that govern the ethical and scientific conduct of clinical investigations sponsored by Merck, is provided in Section 12.1 - Merck Code of Conduct for Clinical Trials.

The investigator also agrees to allow monitoring, audits, IRB/ERC review and regulatory authority inspection of trial-related documents and procedures and provide for direct access to all trial-related source data and documents.

The investigator agrees not to seek reimbursement from subjects, their insurance providers or from government programs for procedures included as part of the trial reimbursed to the investigator by the Sponsor.

The investigator shall prepare and maintain complete and accurate trial documentation in compliance with Good Clinical Practice standards and applicable federal, state and local laws, rules and regulations; and, for each subject participating in the trial, provide all data, and, upon completion or termination of the clinical trial, submit any other reports to the Sponsor as required by this protocol or as otherwise required pursuant to any agreement with the Sponsor.

Trial documentation will be promptly and fully disclosed to the Sponsor by the investigator upon request and also shall be made available at the trial site upon request for inspection, copying, review and audit at reasonable times by representatives of the Sponsor or any regulatory authorities. The investigator agrees to promptly take any reasonable steps that are requested by the Sponsor as a result of an audit to cure deficiencies in the trial documentation and worksheets/case report forms.

The investigator must maintain copies of all documentation and records relating to the conduct of the trial in compliance with all applicable legal and regulatory requirements. This documentation includes, but is not limited to, the protocol, worksheets/case report forms, advertising for subject participation, adverse event reports, subject source data, correspondence with regulatory authorities and IRBs/ERCs, consent forms, investigator's curricula vitae, monitor visit logs, laboratory reference ranges, laboratory certification or quality control procedures and laboratory director curriculum vitae. By signing this protocol, the investigator agrees that documentation shall be retained until at least 2 years after the last approval of a marketing application in an ICH region or until there are no pending or contemplated marketing applications in an ICH region or until at least 2 years have elapsed since the formal discontinuation of clinical development of the investigational product. Because the clinical development and marketing application process is variable, it is anticipated that the retention period can be up to 15 years or longer after protocol database lock. The Sponsor will determine the minimum retention period and notify the investigator when documents may be destroyed. The Sponsor will determine the minimum retention period and upon request, will provide guidance to the investigator when documents no longer need to be retained. The sponsor also recognizes that documents may need to be retained for a longer period if required by local regulatory requirements. All trial documents shall be made available if required by relevant regulatory authorities. The investigator must consult with and obtain written approval by the Sponsor prior to destroying trial and/or subject files.

ICH Good Clinical Practice guidelines recommend that the investigator inform the subject's primary physician about the subject's participation in the trial if the subject has a primary physician and if the subject agrees to the primary physician being informed.

The investigator will promptly inform the Sponsor of any regulatory authority inspection conducted for this trial.

Persons debarred from conducting or working on clinical trials by any court or regulatory authority will not be allowed to conduct or work on this Sponsor's trials. The investigator will immediately disclose in writing to the Sponsor if any person who is involved in conducting the trial is debarred or if any proceeding for debarment is pending or, to the best of the investigator's knowledge, threatened.

In the event the Sponsor prematurely terminates a particular trial site, the Sponsor will promptly notify that trial site's IRB/IEC.

According to European legislation, a Sponsor must designate an overall coordinating investigator for a multi-center trial (including multinational). When more than one trial site is open in an EU country, Merck, as the Sponsor, will designate, per country, a national principal coordinator (Protocol CI), responsible for coordinating the work of the principal investigators at the different trial sites in that Member State, according to national regulations. For a single-center trial, the Protocol CI is the principal investigator. In addition, the Sponsor must designate a principal or coordinating investigator to review the trial report that summarizes the trial results and confirm that, to the best of his/her knowledge, the report accurately describes the conduct and results of the trial [Clinical Study Report (CSR) CI]. The Sponsor may consider one or more factors in the selection of the individual to serve as the Protocol CI and or CSR CI (e.g., availability of the CI during the anticipated review process, thorough understanding of clinical trial methods, appropriate enrollment of subject cohort, timely achievement of trial milestones). The Protocol CI must be a participating trial investigator.

10.4 Compliance with Trial Registration and Results Posting Requirements

Under the terms of the Food and Drug Administration Amendments Act (FDAAA) of 2007, and the European Medicines Agency (EMA) clinical trial Directive 2001/20/EC, the Sponsor of the trial is solely responsible for determining whether the trial and its results are subject to the requirements for submission to http://www.clinicaltrials.gov, www.clinicaltrialregister.eu or other local registries. Merck, as Sponsor of this trial, will review this protocol and submit the information necessary to fulfill these requirements. Merck entries are not limited to FDAAA or the EMA clinical trials directive mandated trials. Information posted will allow subjects to identify potentially appropriate trials for their disease conditions and pursue participation by calling a central contact number for further information on appropriate trial locations and trial site contact information.

By signing this protocol, the investigator acknowledges that the statutory obligations under FDAAA, the EMA clinical trials directive or other locally mandated registries are that of the Sponsor and agrees not to submit any information about this trial or its results to those registries.

10.5 Quality Management System

By signing this protocol, the Sponsor agrees to be responsible for implementing and maintaining a quality management system with written development procedures and functional area standard operating procedures (SOPs) to ensure that trials are conducted and data are generated, documented, and reported in compliance with the protocol, accepted standards of Good Clinical Practice, and all applicable federal, state, and local laws, rules and regulations relating to the conduct of the clinical trial.

10.6 Data Management

The investigator or qualified designee is responsible for recording and verifying the accuracy of subject data. By signing this protocol, the investigator acknowledges that his/her electronic signature is the legally binding equivalent of a written signature. By entering his/her electronic signature, the investigator confirms that all recorded data have been verified as accurate.

Detailed information regarding Data Management procedures for this protocol will be provided separately.

10.7 Publications

This trial is intended for publication, even if terminated prematurely. Publication may include any or all of the following: posting of a synopsis online, abstract and/or presentation at a scientific conference, or publication of a full manuscript. The Sponsor will work with the authors to submit a manuscript describing trial results within 12 months after the last data become available, which may take up to several months after the last subject visit in some cases such as vaccine trials. However, manuscript submission timelines may be extended on OTC trials. For trials intended for pediatric-related regulatory filings, the investigator agrees to delay publication of the trial results until the Sponsor notifies the investigator that all relevant regulatory authority decisions on the trial drug have been made with regard to pediatric-related regulatory filings. Merck will post a synopsis of trial results for approved products on www.clinicaltrials.gov by 12 months after the last subject's last visit for the primary outcome, 12 months after the decision to discontinue development, or product marketing (dispensed, administered, delivered or promoted), whichever is later.

These timelines may be extended for products that are not yet marketed, if additional time is needed for analysis, to protect intellectual property, or to comply with confidentiality agreements with other parties. Authors of the primary results manuscript will be provided the complete results from the Clinical Study Report, subject to the confidentiality agreement. When a manuscript is submitted to a biomedical journal, the Sponsor's policy is to also include the protocol and statistical analysis plan to facilitate the peer and editorial review of the manuscript. If the manuscript is subsequently accepted for publication, the Sponsor will allow the journal, if it so desires, to post on its website the key sections of the protocol that are relevant to evaluating the trial, specifically those sections describing the trial objectives and hypotheses, the subject inclusion and exclusion criteria, the trial design and procedures, the efficacy and safety measures, the statistical analysis plan, and any amendments relating to those sections. The Sponsor reserves the right to redact proprietary information.

For multicenter trials, subsequent to the multicenter publication (or after public disclosure of the results online at www.clinicaltrials.gov if a multicenter manuscript is not planned), an investigator and his/her colleagues may publish their data independently. In most cases, publication of individual trial site data does not add value to complete multicenter results, due to statistical concerns. In rare cases, publication of single trial site data prior to the main paper may be of value. Limitations of single trial site observations in a multicenter trial should always be described in such a manuscript.

Authorship credit should be based on 1) substantial contributions to conception and design, or acquisition of data, or analysis and interpretation of data; 2) drafting the article or revising it critically for important intellectual content; and 3) final approval of the version to be published. Authors must meet conditions 1, 2 and 3. Significant contributions to trial execution may also be taken into account to determine authorship, provided that contributions have also been made to all three of the preceding authorship criteria. Although publication planning may begin before conducting the trial, final decisions on authorship and the order of authors' names will be made based on participation and actual contributions to the trial and writing, as discussed above. The first author is responsible for defending the integrity of the data, method(s) of data analysis and the scientific content of the manuscript.

The Sponsor must have the opportunity to review all proposed abstracts, manuscripts or presentations regarding this trial 45 days prior to submission for publication/presentation. Any information identified by the Sponsor as confidential must be deleted prior to submission; this confidentiality does not include efficacy and safety results. Sponsor review can be expedited to meet publication timelines.

11.0 LIST OF REFERENCES

1. U.S. Prescribing Information: ZOSYN (piperacillin/tazobactam) for Injection: single-dose and pharmacy bulk vials, ZOSYN (piperacillin/tazobactam) Injection: single dose GALAXY containers: 2013.1
2. U.S. Product Circular: ZYVOX (linezolid) injection, tablets and for oral suspension: 2011.
3. Niederman MS, Craven DE. American Thoracic Society Documents: Guidelines for the management of adults with hospital-acquired, ventilator-associated, and healthcare-associated pneumonia. Am J Respir Crit Care Med 2005;171: 388-416.
4. Liu C, Bayer A, Cosgrove SE, Daum RS, Fridkin SK, Gorwitz RJ, et al. Clinical practice guidelines by the infectious diseases society of america for the treatment of methicillin-resistant Staphylococcus aureus infections in adults and children: executive summary. Clin Infect Dis. 2011 Feb 1;52(3):285-92.
5. Wunderink RG, Niederman MS, Kollef MH, et al. Linezolid in methicillin-resistant Staphylococcus aureus nosocomial pneumonia: a randomized, controlled study. Clin Infect Dis 2012; 54:621-629.
6. Kollef MH, Chastre J, Clavel M, Restrepo MI, Michiels B, Kaniga K, et al. A randomized trial of 7-day doripenem versus 10-day imipenem-cilastatin for ventilator associated pneumonia. Crit Care 2012;16:1-17.
7. Freire AT, Melnyk V, Kim MJ, Datsenko O, Dzyublik O, Glumcher F, et al. Comparison of tigecycline with imipenem/cilastatin for the treatment of hospital-acquired pneumonia. Diagn Microbiol Infect Dis 2010;68:140-51.
8. Rubinstein, E, Tahaniyat, L, et al. Telavancin versus Vancomycin for Hospital-Acquired Pneumonia due to Gram-positive Pathogens. Clin Infect Dis 2011; 52(1):31-40.
9. Safdar N, Dezfulian C, Collard HR, Saint S. Clinical and economic consequences of ventilator-associated pneumonia: A systematic review. Crit Care Med 2005;33(10):2184-93.
10. Hyle EP, Lipworth AD, Zaoutis TE, Nachamkin I, Bilker WB, Lautenbach E. Impact of inadequate initial antimicrobial therapy on mortality in infections due to extended spectrum-lactamase-producing enterobacteriaceae: Variability by site of infection. Arch Intern Med 2005;165:1375-80.
11. Ramirez J, Dartois N, Gandjini H, Yan JL, Korth-Bradley J, McGovern PC. Randomized phase 2 trial to evaluate the clinical efficacy of two high-dosage tigecycline regimens versus imipenem-cilastatin for treatment of hospital-acquired pneumonia. Antibiot Chemother 2013;57(4):1756-62.
12. Joshi M, Metzler M, McCarthy M, Olvey S, Kassira W, Cooper A. Comparison of piperacillin/tazobactam and imipenem/cilastatin, both in combination with tobramycin, administered every 6 h for treatment of nosocomial pneumonia. Respir Med 2006;100:1554-65.

13. Schmitt DV, Leitner E, Welte T, Lode H. Piperacillin/tazobactam vs. imipenem/cilastatin in the treatment of nosocomial pneumonia - a double blind prosepective multicentre study. Infection 2006; 34(3):127-34.

14. Freire AT, Melnyk V, Kim MJ, Datsenko O, Dzyublik O, Glumcher F, et al. Comparison of tigecycline with imipenem/cilastatin for the treatment of hospital-acquired pneumonia. Diagn Microbiol Infect Dis 2010;68:140-51.

15. Chastre J, Wunderink R, Prokocimer P, Lee M, Kaniga K, Friedland I. Efficacy and safety of intravenous infusion of doripenem versus imipenem in ventilator-associated pneumonia: a multicenter, randomized study. Crit Care Med 2008;36(4):1089-96.

16. Pugh R, Grant C, Cooke RP, Dempsey G. Short-course versus prolonged-course antibiotic therapy for hospital-acquired pneumonia in critically ill adults. Cochrane Database Syst Rev. 2011 Oct 5;(10):CD007577.

17. Chastre J, Wolff M, Fagon JY, Chevret S, Thomas F, Wermert D, et al. Comparison of 8 vs. 15 days of antibiotic therapy for ventilator-associated pneumonia in adults: a randomized trial. JAMA. 2003 Nov 19; 290(19):2588-98.

18. West M, Boulanger BR, Fogarty C, Tennenberg A, Wiesinger B, Oross M, et al. Levofloxacin compared with imipenem/cilastatin followed by ciprofloxacin in adult patients with nosocomial pneumonia: a multicenter, prospective, randomized, open-label study. Clin Ther 2003;25(2):485-506.

19. Torres A, Bauer TT, León-Gil C, Castillo F, Alvarez-Lerma F, Martínez-Pellús A, et al. Treatment of severe nosocomial pneumonia: a prospective randomised comparison of intravenous ciprofloxacin with imipenem/cilastatin. Thorax 2000;55(12):1033-9.

20. Knaus WA, Draper EA, Wagner DP, Zimmerman JE. APACHE II: a severity of disease classification system. Crit Care Med 1985; 13(10):818-29.

21. M. Zilberberg and A. Shorr. Ventilator-Associated Pneumonia: The Clinical Pulmonary Infection Score as a Surrogate for Diagnostics and Outcome. *Clin Infect Dis,* 2010;51(S1): S131–S135.

22. Robert J. Hemmings & John A. Lewis . The Implications of Current Methodological Issues for International Guidance. Statistics in Biopharmaceutical Research, 2013;5:3, 211-222.

23. Food and Drug Administration, Center for Drug Evaluation and Research (CDER). Guidance for Industry: Hospital-acquired bacterial pneumonia and ventilator- associated bacterial pneumonia: developing drugs for treatment [Internet]. Silver Spring, MD: Department of Health and Human Services; 2014 [Updated May 2014]. Available from: http://www.fda.gov/Drugs/GuidanceComplianceRegulatoryInformation/Guidances/default.htm

24. Miettinen O, Nurminen M. Comparative analysis of two rates. Stat Med 1985;4:213

12.0 APPENDICES

12.1 Merck Code of Conduct for Clinical Trials

Merck*
Code of Conduct for Clinical Trials

I. Introduction

A. Purpose

Merck, through its subsidiaries, conducts clinical trials worldwide to evaluate the safety and effectiveness of our products. As such, we are committed to designing, implementing, conducting, analyzing and reporting these trials in compliance with the highest ethical and scientific standards. Protection of subject safety is the overriding concern in the design of clinical trials. In all cases, Merck clinical trials will be conducted in compliance with local and/or national regulations and in accordance with the ethical principles that have their origin in the Declaration of Helsinki.

B. Scope

Such standards shall be endorsed for all clinical interventional investigations sponsored by Merck irrespective of the party (parties) employed for their execution (e.g., contract research organizations, collaborative research efforts). This Code is not intended to apply to trials which are observational in nature, or which are retrospective. Further, this Code does not apply to investigator-initiated trials which are not under the control of Merck.

II. Scientific Issues

A. Trial Conduct

1. Trial Design

Except for pilot or estimation trials, clinical trial protocols will be hypothesis-driven to assess safety, efficacy and/or pharmacokinetic or pharmacodynamic indices of Merck or comparator products. Alternatively, Merck may conduct outcomes research trials, trials to assess or validate various endpoint measures, or trials to determine subject preferences, etc.

The design (i.e., subject population, duration, statistical power) must be adequate to address the specific purpose of the trial. Research subjects must meet protocol entry criteria to be enrolled in the trial.

2. Site Selection

Merck selects investigative sites based on medical expertise, access to appropriate subjects, adequacy of facilities and staff, previous performance in Merck trials, as well as budgetary considerations. Prior to trial initiation, sites are evaluated by Merck personnel to assess the ability to successfully conduct the trial.

3. Site Monitoring/Scientific Integrity

Trial sites are monitored to assess compliance with the trial protocol and general principles of Good Clinical Practice. Merck reviews clinical data for accuracy, completeness and consistency. Data are verified versus source documentation according to standard operating procedures. Per Merck policies and procedures, if fraud, misconduct or serious GCP-non-Compliance are suspected, the issues are promptly investigated. When necessary, the clinical site will be closed, the responsible regulatory authorities and ethics review committees notified and data disclosed accordingly.

B. Publication and Authorship

To the extent scientifically appropriate, Merck seeks to publish the results of trials it conducts. Some early phase or pilot trials are intended to be hypothesis-generating rather than hypothesis testing. In such cases, publication of results may not be appropriate since the trial may be underpowered and the analyses complicated by statistical issues of multiplicity.

Merck's policy on authorship is consistent with the requirements outlined in the ICH-Good Clinical Practice guidelines. In summary, authorship should reflect significant contribution to the design and conduct of the trial, performance or interpretation of the analysis, and/or writing of the manuscript. All named authors must be able to defend the trial results and conclusions. Merck funding of a trial will be acknowledged in publications.

III. Subject Protection

A. IRB/ERC review

All clinical trials will be reviewed and approved by an independent IRB/ERC before being initiated at each site. Significant changes or revisions to the protocol will be approved by the IRB/ERC prior to implementation, except that changes required urgently to protect subject safety and well-being may be enacted in anticipation of IRB/ERC approval. For each site, the IRB/ERC and Merck will approve the subject informed consent form.

B. Safety

The guiding principle in decision-making in clinical trials is that subject welfare is of primary importance. Potential subjects will be informed of the risks and benefits of, as well as alternatives to, trial participation. At a minimum, trial designs will take into account the local standard of care. Subjects are never denied access to appropriate medical care based on participation in a Merck clinical trial.

All participation in Merck clinical trials is voluntary. Subjects are enrolled only after providing informed consent for participation. Subjects may withdraw from a Merck trial at any time, without any influence on their access to, or receipt of, medical care that may otherwise be available to them.

C. Confidentiality

Merck is committed to safeguarding subject confidentiality, to the greatest extent possible. Unless required by law, only the investigator, sponsor (or representative) and/or regulatory authorities will have access to confidential medical records that might identify the research subject by name.

D. Genomic Research

Genomic Research will only be conducted in accordance with informed consent and/or as specifically authorized by an Ethics Committee.

IV. Financial Considerations

A. Payments to Investigators

Clinical trials are time- and labor-intensive. It is Merck's policy to compensate investigators (or the sponsoring institution) in a fair manner for the work performed in support of Merck trials. Merck does not pay incentives to enroll subjects in its trials. However, when enrollment is particularly challenging, additional payments may be made to compensate for the time spent in extra recruiting efforts.

Merck does not pay for subject referrals. However, Merck may compensate referring physicians for time spent on chart review to identify potentially eligible subjects.

B. Clinical Research Funding

Informed consent forms will disclose that the trial is sponsored by Merck, and that the investigator or sponsoring institution is being paid or provided a grant for performing the trial. However, the local IRB/ERC may wish to alter the wording of the disclosure statement to be consistent with financial practices at that institution. As noted above, publications resulting from Merck trials will indicate Merck as a source of funding.

C. Funding for Travel and Other Requests

Funding of travel by investigators and support staff (e.g., to scientific meetings, investigator meetings, etc.) will be consistent with local guidelines and practices including, in the U.S., those established by the American Medical Association (AMA).

V. Investigator Commitment

Investigators will be expected to review Merck's Code of Conduct as an appendix to the trial protocol, and in signing the protocol, agree to support these ethical and scientific standards.

* In this document, "Merck" refers to Merck Sharp & Dohme Corp. and Schering Corporation, each of which is a subsidiary of Merck & Co., Inc. Merck is known as MSD outside of the United States and Canada. As warranted by context, Merck also includes affiliates and subsidiaries of Merck & Co., Inc."

12.2 Collection and Management of Specimens for Future Biomedical Research

1. Definitions

a. Biomarker: A biological molecule found in blood, other body fluids, or tissues that is a sign of a normal or abnormal process or of a condition or disease. A biomarker may be used to see how well the body responds to a treatment for a disease or condition.[1]

b. Pharmacogenomics: The investigation of variations of DNA and RNA characteristics as related to drug/vaccine response.[2]

c. Pharmacogenetics: A subset of pharmacogenomics, pharmacogenetics is the influence of variations in DNA sequence on drug/vaccine response.[2]

d. DNA: Deoxyribonucleic acid.

e. RNA: Ribonucleic acid.

2. Scope of Future Biomedical Research

The specimens collected in this trial as outlined in Section 7.1.3.3 – Future Biomedical Research will be used to study various causes for how subjects may respond to a drug/vaccine. Future biomedical research specimen(s) will be stored to provide a resource for future trials conducted by Merck focused on the study of biomarkers responsible for how a drug/vaccine enters and is removed by the body, how a drug/vaccine works, other pathways a drug/vaccine may interact with, or other aspects of disease. The specimen(s) may be used for future assay development and/or drug/vaccine development.

It is now well recognized that information obtained from studying and testing clinical specimens offers unique opportunities to enhance our understanding of how individuals respond to drugs/vaccines, enhance our understanding of human disease and ultimately improve public health through development of novel treatments targeted to populations with the greatest need. All specimens will be used by Merck or designees and research will be monitored and reviewed by a committee of our scientists and clinicians.

3. Summary of Procedures for Future Biomedical Research

a. Subjects for Enrollment

All subjects enrolled in the clinical trial will be considered for enrollment in the Future Biomedical Research sub-trial.

b. Informed Consent

Informed consent for specimens (i.e., DNA, RNA, protein, etc.) will be obtained during screening for protocol enrollment from all subjects or legal guardians, at a trial visit by the investigator or his or her designate. Informed consent for Future Biomedical Research should be presented to the subjects on Visit 1. If delayed, present consent at next possible Subject Visit. Informed consent must be obtained prior to collection of all Future Biomedical Research specimens. Consent forms signed by the subject will be kept at the clinical trial site under secure storage for regulatory reasons. Information contained on the consent form alone cannot be traced

to any specimens, test results, or medical information once the specimens have been rendered de-identified

A template of each trial site's approved informed consent will be stored in the Sponsor's clinical document repository. Each consent will be assessed for appropriate specimen permissions.

Each informed consent approved by an ethics committee is assigned a unique tracking number. The tracking number on this document will be used to assign specimen permissions for each specimen into the Entrusted Keyholder's Specimen Database.

c. eCRF Documentation for Future Biomedical Research Specimens

Documentation of both consent and acquisition of Future Biomedical Research specimens will be captured in the electronic Case Report Forms (eCRFs). Reconciliation of both forms will be performed to assure that only appropriately-consented specimens are used for this sub-trial's research purposes. Any specimens for which such an informed consent cannot be verified will be destroyed.

d. Future Biomedical Research Specimen Collections

Collection of specimens for Future Biomedical Research will be performed as outlined in the trial flow chart. In general, if additional blood specimens are being collected for Future Biomedical Research, these will usually be obtained at a time when the subject is having blood drawn for other trial purposes.

4. Confidential Subject Information for Future Biomedical Research

In order to optimize the research that can be conducted with Future Biomedical Research specimens, it is critical to link subject' clinical information with future test results. In fact little or no research can be conducted without connecting the clinical trial data to the specimen. The clinical data allow specific analyses to be conducted. Knowing subject characteristics like gender, age, medical history and treatment outcomes are critical to understanding clinical context of analytical results.

To maintain privacy of information collected from specimens obtained for Future Biomedical Research, Merck has developed secure policies and procedures. All specimens will be de-identified as described below.

At the clinical trial site, unique codes will be placed on the Future Biomedical Research specimens for transfer to the storage facility. This first code is a random number which does not contain any personally identifying information embedded within it. The link (or key) between subject identifiers and this first unique code will be held at the trial site. No personal identifiers will appear on the specimen tube.

This first code will be replaced with a second code at a Merck designated storage/lab facility. The second code is linked to the first code via a second key. The specimen is now double coded. Specimens with the second code are sometimes referred to as de-identified specimens. The use of the second code provides additional confidentiality and privacy protection for subjects over the use of a single code. Access to both keys would be needed to link any data or specimens back to the subject's identification.

The second code is stored separately from the first code and all associated personal specimen identifiers. A secure link, the second key, will be utilized to match the second code to the first code to allow clinical information collected during the course of the trial to be associated with the specimen. This second key will be transferred under secure procedures by the Merck designated facility to an Entrusted Keyholder at Merck. The second code will be logged into the primary biorepository database at Merck and, in this database, this identifier will not have identifying demographic data or identifying clinical information (i.e., race, sex, age, diagnosis, lab values) associated with it. The specimen will be stored in a designated biorepository site with secure policies and procedures for specimen storage and usage.

The second key can be utilized to reconstruct the link between the results of future biomedical research and the clinical information, at the time of analysis. This linkage would not be possible for the scientist conducting the analysis, but can only be done by the Merck Entrusted Keyholder under strict security policies and procedures. The Merck Entrusted Keyholder will link the information and then issue a de-identified data set for analysis. The only other circumstance by which future biomedical research data would be directly linked to the full clinical data set would be those situations mandated by regulatory authorities (e.g., EMEA, FDA), whereby this information would be directly transferred to the regulatory authority.

5. Biorepository Specimen Usage

Specimens obtained for the Merck Biorepository will be used for analyses using good scientific practices. However, exploratory analyses will not be conducted under the highly validated conditions usually associated with regulatory approval of diagnostics. The scope of research performed on these specimens is limited to the investigation of the variability in biomarkers that may correlate with a clinical phenotype in subjects.

Analyses utilizing the Future Biomedical Research specimens may be performed by Merck, or an additional third party (e.g., a university investigator) designated by Merck. The investigator conducting the analysis will be provided with double coded specimens. Re-association of analysis results with corresponding clinical data will only be conducted by the Merck Entrusted Keyholder. Any contracted third party analyses will conform to the specific scope of analysis outlined in this sub-trial. Future Biomedical Research specimens remaining with the third party after the specific analysis is performed will be returned to the sponsor or destroyed and documentation of destruction will be reported to Merck.

6. Withdrawal From Future Biomedical Research

Subjects may withdraw their consent for Future Biomedical Research and have their specimens and all derivatives destroyed. Subjects may withdraw consent at any time by contacting the principal investigator for the main trial. If medical records for the main trial are still available, the investigator will contact Merck using the designated mailbox (clinical.specimen.management@merck.com) and a form will be provided by Merck to obtain appropriate information to complete specimen withdrawal. Subsequently, the subject's specimens will be removed from the biorepository and be destroyed. A letter will be sent from Merck to the investigator confirming the destruction. It is the responsibility of the investigator to inform the subject of completion of destruction. Any

analyses in progress at the time of request for destruction or already performed prior to the request being received by the Sponsor will continue to be used as part of the overall research trial data and results. No new analyses would be generated after the request is received.

In the event that the medical records for the main trial are no longer available (e.g., if the investigator is no longer required by regulatory authorities to retain the main trial records) or the specimens have been completely anonymized, there will no longer be a link between the subject's personal information and their specimens. In this situation, the request for specimen destruction can not be processed.

7. Retention of Specimens

Future Biomedical Research specimens will be stored in the biorepository for potential analysis for up to 20 years from acquisition. Specimens may be stored for longer if a regulatory or governmental authority has active questions that are being answered. In this special circumstance, specimens will be stored until these questions have been adequately addressed.

Specimens from the trial site will be shipped to a central laboratory and then shipped to the Merck designated biorepository. The specimens will be stored under strict supervision in a limited access facility which operates to assure the integrity of the specimens. Specimens will be destroyed according to Merck policies and procedures and this destruction will be documented in the biorepository database.

8. Data Security

Separate databases for specimen information and for results from the Future Biomedical Research sub-trial will be maintained by Merck. This is done to separate the future exploratory test results (which include genetic data) from the clinical trial database thereby maintaining a separation of subject number and these results. The separate databases are accessible only to the authorized Sponsor and the designated trial administrator research personnel and/or collaborators. Database user authentication is highly secure, and is accomplished using network security policies and practices based in international standards (e.g., ISO17799) to protect against unauthorized access. The Merck Entrusted Keyholder maintains control over access to all specimen data. These data are collected for future biomedical research purposes only as specified in this sub-trial will not be used for any other purpose.

9. Reporting of Future Biomedical Research Data to Subjects

There is no definitive requirement in either authoritative ethical guidelines or in relevant laws/regulations globally that research results have to be, in all circumstances, returned to the trial participant. Some guidelines advocate a proactive return of data in certain instances. No information obtained from exploratory laboratory studies will be reported to the subject or family, and this information will not be entered into the clinical database maintained by Merck on subjects. Principle reasons not to inform or return results to the subject include: lack of relevance to subject health, limitations of predictive capability, concerns of misinterpretation and absence of good clinical practice standards in exploratory research typically used for diagnostic testing.

If any exploratory results are definitively associated with clinical significance for subjects while the clinical trial is still ongoing, investigators will be contacted with information as to how to offer clinical diagnostic testing (paid for by Merck) to subjects enrolled and will be advised that counseling should be made available for all who choose to participate in this diagnostic testing.

If any exploratory results are definitively associated with clinical significance after completion of a clinical trial, Merck will publish the results without revealing specific subject information, inform all trial sites who participated in the Merck clinical trial and post anonymized results on our website or other accredited website(s) that allow for public access (e.g., disease societies who have primary interest in the results) in order that physicians and patients may pursue clinical diagnostic testing if they wish to do so.

10. Gender, Ethnicity and Minorities

Although many diagnoses differ in terms of frequency by ethnic population and gender, every effort will be made to recruit all subjects diagnosed and treated on Merck clinical trials for future biomedical research. When trials with specimens are conducted and subjects identified to serve as controls, every effort will be made to group specimens from subjects and controls to represent the ethnic and gender population representative of the disease under current investigation.

11. Risks Versus Benefits of Future Biomedical Research

For future biomedical research, risks to the subject have been minimized. Risks include those associated with venipuncture to obtain the whole blood specimen. This specimen will be obtained at the time of routine blood specimens drawn in the main trial.

Merck has developed strict security, policies and procedures to address subject data privacy concerns. Data privacy risks are largely limited to rare situations involving possible breach of confidentiality. In this highly unlikely situation there is risk that the information, like all medical information, may be misused.

It is necessary for subject-related data (i.e., ethnicity, diagnosis, drug therapy and dosage, age, toxicities, etc.) to be re-associated to double coded specimens at the time of data analysis. These subject data will be kept in a separate, secure Merck database, and all specimens will be stripped of subject identifiers. No information concerning results obtained from future biomedical research will be entered into clinical records, nor will it be released to outside persons or agencies, in any way that could be tied to an individual subject.

12. Self-Reported Ethnicity

Subjects who participate in future biomedical research will be asked to provide self-reported ethnicity. Subjects who do not wish to provide this data may still participate in future biomedical research.

13. Questions

Any questions related to the future biomedical research should be e-mailed directly to clinical.specimen.management@merck.com.

14. References

1. National Cancer Institute: http://www.cancer.gov/dictionary/?searchTxt=biomarker
2. International Conference on Harmonization: DEFINITIONS FOR GENOMIC BIOMARKERS, PHARMACOGENOMICS, PHARMACOGENETICS, GENOMIC DATA AND SAMPLE CODING CATEGORIES - E15; http://www.ich.org/LOB/media/MEDIA3383.pdf

12.3 Pharmacogenetics Informational Brochure for IRBs/IECs & Investigational Site Staff

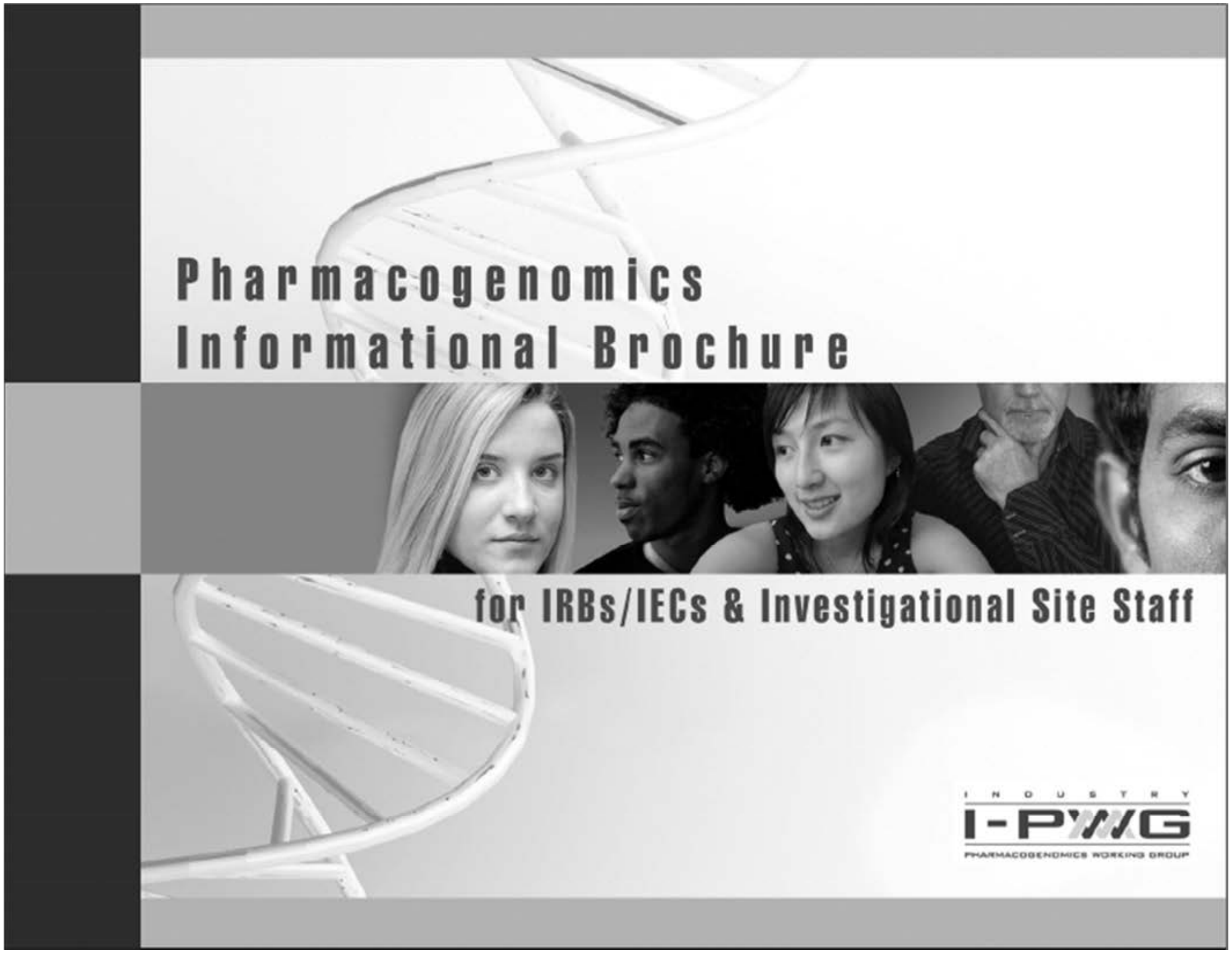

This Informational Brochure is intended for IRBs/IECs & Investigational Site Staff. The brochure was developed to address issues relevant to DNA collection and research in the context of pharmaceutical drug development.

Developed by
The Industry Pharmacogenomics Working Group (I-PWG)
www.i-pwg.org

What is DNA and What is Pharmacogenomics?

The cells of the body contain **deoxyribonucleic acid** (**DNA**). DNA is inherited, and carries a code (in the form of **genes**), which determines physical appearance and other personal features. In a process called gene transcription, DNA is copied into a related molecule, ribonucleic acid (RNA), before ultimately being translated into proteins, which determine cellular function. Naturally-occurring variation in DNA is a major determinant of differences among people. This variation, referred to as **genetic polymorphism**, occurs both within genes and outside of genes throughout the entire **human genome**. This variation partly explains why some people develop certain diseases and others do not, why some people respond better than others to certain drugs, and why some people develop side effects while others do not.

Pharmacogenomics (PGx) is a branch of science that uses genetic/genomic information to better understand why people respond differently to drugs. The terms **pharmacogenomics** and **pharmacogenetics** are often used interchangeably, although pharmacogenetics generally refers to the study of DNA, while pharmacogenomics is a broader term encompassing the study of both DNA and RNA[1], and generally on a larger scale. Pharmacogenomic research is different from **genetic testing** done for the purpose of diagnosing a person with a certain disease or for risk for developing a certain disease (e.g., genetic testing for Huntington's Disease). PGx focuses on genetic variability that affects response to drugs. This primarily occurs through pathways related to drug metabolism, drug mechanism of action, disease etiology or subtype, and adverse events. PGx overlaps with **disease genetics** research since different disease subtypes can respond differently to drugs.

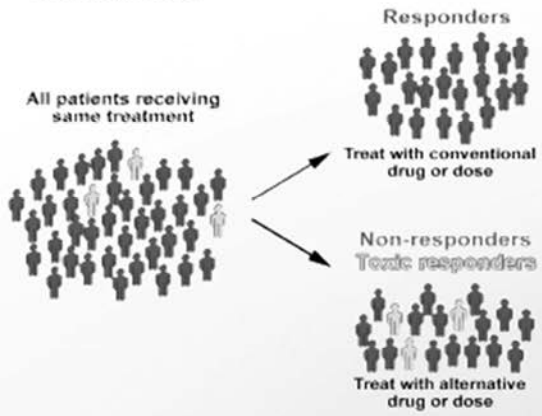

Why is Pharmacogenomics Important?

PGx is one approach to explore whether a drug will be useful or harmful in certain people. By identifying genetic polymorphisms that are associated with drug efficacy and safety, PGx is allowing for more individualized drug therapies based on the genetic makeup of patients. This is sometimes referred to as **personalized medicine**. By better understanding diseases at the molecular level, PGx is opening opportunities for the discovery of novel drugs.

1

PGx has the overarching goal of developing safer, more effective drugs, and ensuring that patients receive the correct dose of the correct drug at the correct time.

How is Pharmacogenomics Being Used in Drug Development?

PGx is increasingly becoming a core component of drug development programs. By using PGx to determine how drugs work differently in subgroups of patients, drug developers are making better decisions about which drugs to develop and how best to develop them. Technologies are now available to simultaneously analyze over 1 million genetic polymorphisms in the human genome. This is allowing for the identification of novel genetic markers of drug response and of disease in absence of pre-existing knowledge of the involvement of specific pathways.

PGx research is currently being used in drug development to:

- Explain variability in response among subjects in clinical trials
- Address emerging clinical issues, such as unexpected adverse events
- Determine eligibility for clinical trials (pre-screening) to optimize trial design
- Develop drug-linked diagnostic tests to identify patients who are more likely or less likely to benefit from treatment or who may be at risk of adverse events
- Better understand the mechanism of action or metabolism of new and existing drugs
- Provide better understanding of disease mechanisms
- Allow physicians to prescribe the right drugs at the optimal dose for individual patients

Pharmacogenomics Already a Reality in Drug Labels

A number of drugs now have instructions on their labels either recommending or requiring a PGx test when prescribing a drug or when making dosing decisions. A well-known example is the anti-coagulant drug *warfarin*. The drug label for warfarin now includes a recommended PGx test to minimize the risk of excessive bleeding (US label). There are currently three categories of PGx information in drug labels according to the FDA:

i) tests **required** for prescribing

ii) tests **recommended** when prescribing

iii) PGx information **for information only**.

For a current list of examples of how PGx is impacting drug labeling see:
www.fda.gov/Drugs/ScienceResearch/ResearchAreas/Pharmacogenetics/ucm083378.htm

DNA Samples from Clinical Trials An Invaluable Resource

Adequate sample sizes and high-quality clinical data are key to advancements in the field of PGx. Drug development programs are therefore an invaluable resource and a unique opportunity for highly productive research in PGx. Although PGx is a rapidly evolving branch of science, the complexities of the genetic code are only beginning to be understood. As scientific discoveries continue to be made, samples collected today will become a valuable resource

I-PWG

for future research. This may lead to the future development of new drugs that are better targeted to certain individuals and to disease subtypes.

For these reasons, it is vital to systematically collect DNA samples across all centers recruiting subjects into clinical trials that include a PGx component (where local regulations permit). Consent for storage of samples for future research should also be obtained if maximum benefit is to be derived from DNA samples donated by subjects. The scope of the research that may be performed both during the trial and in the future should be clearly defined in the informed consent form.

Informed Consent

Policies and regulations for legally effective informed consent vary on national, state, and local levels. There currently are no internationally recognized regulations that dictate the basic elements of informed consent for PGx research. The I-PWG has published an article on the elements of informed consent to be considered in PGx research studies[2]. These elements build upon existing basic elements of informed consent for clinical research on human subjects[3].

Return of Genomic Research Results to Study Subjects

Policies for the return of genomic results to study subjects vary among pharmaceutical companies. There are many considerations that pharmaceutical companies weigh when determining their policy regarding the return of PGx research results to study subjects. These include i) the conditions under which genomic results were generated (i.e., research laboratory environment versus accredited diagnostic laboratory), ii) whether the results will have an impact on patient medical care, iii) whether genetic counseling is necessary, and iv) international, national, and local guidelines, policies, legislation, and regulations regarding subjects' rights to access data generated on them. These considerations are addressed in detail in Renegar et al. 2006[4].

Privacy, Confidentiality, and Patient Rights

An issue that is generally perceived to be of relevance to clinical genetic research is the risk associated with inadvertent or intentional disclosure and misuse of genetic data. Although coded specimens generally have been considered adequate to protect patient privacy in most clinical development, companies and other institutions involved in PGx research have historically applied a variety of additional safeguards that can be used alone, or in combination, to further minimize the potential risk of disclosure and misuse of genetic data. These include:

i) Sample Labeling

DNA samples and corresponding clinical data can be labeled in several ways to achieve different levels of patient privacy and confidentiality. Definitions of labeling methods are provided in the glossary and are described in greater detail in the ICH Guidance E15[1]. It is important to recognize that there is a trade-off between the level of patient privacy protection and the ability to perform actions related to withdrawal of consent, data return, clinical monitoring, subject follow-up, and addition of new data (see Table 1)[1]. The *Identified* and *Anonymous* labeling categories described in the table are generally not applicable to pharmaceutical clinical trials.

I-PWG

Table adapted from ICH Guidance E15

Sample Coding Category		Link Between Subject's Personal Identifiers and Genomic Biomarker Data	Traceability back to the Subject (Actions Possible, Including e.g., Sample Withdrawal or Return of Individual Genomic Results at Subject's Request	Ability to Perform Clinical Monitoring, Subject Follow-up, or Addition of New Data	Extent of Subject's Confidentiality and Privacy Protection
Identified		Yes (Direct)<br>Allows for Subjects to be Identified	Yes	Yes	Similar to General Healthcare Confidentiality and Privacy
Coded	Single	Yes (Indirectly)<br>Allows for Subjects to be Identified (via Single, Specific Coding Key)	Yes	Yes	Standard for Clinical Research
	Double	Yes (Very Indirectly)<br>Allows for Subjects to be Identified (via the Two Specific Coding Keys)	Yes	Yes	Added Privacy and Confidentiality Protection over Single Code
Anonymized		No<br>Does not Allow Subject to be Re-Identified as the Coding-Key(s) Have Been Deleted	No	No	Genomic Data and Samples no Longer Linked to Subject as Coding Key(s) have been Deleted
Anonymous		No – Identifiers Never Collected and Coding Keys Never Applied. Does not Allow for Subjects to be Identified	No	No	Genomic Data and Samples Never Linked to Subject

ii) Separation of Data and Restricted Access

- Maintaining PGx-related documentation separate from other medical records.
- Restricting access to data and samples by means of password-protected databases and locked sample storage facilities.

PGx studies in pharmaceutical development are generally conducted in research laboratories that are not accredited diagnostic laboratories. Therefore, PGx research data usually cannot be used to make clinically meaningful or reliable decisions about a subject's health or health risks. Furthermore, confidentiality protections described above serve to guard against inappropriate disclosure of these data. For these reasons, the potential risk to a subject's employment or health/life insurance is considered to be minimal. The measures taken to protect subjects against reasonably foreseeable risks should be addressed in the informed consent form[2].

4

I-PWG

iii) Legislation on Genetic Discrimination

Many countries and regions have enacted legislation to protect individuals against discrimination based on their genetic information. For example, the USA Genetic Non-discrimination Act (GINA)[5, 6] serves to protect patients against health insurance and employment discrimination based on an individual's genetic make-up. Legislation continually evolves based on social, ethical, and legal considerations. A list of examples is periodically updated on the I-PWG website: http://www.i-pwg.org

Country-Specific Laws and Regulations on DNA Collection

DNA sampling in clinical trials is straightforward in most jurisdictions. However, some countries have specific laws and regulations regarding collection, labeling, storage, export, return of results, and/or use of DNA samples. Processes for the collection of DNA samples should always adhere to the regulations of the country/region in which those samples are collected. Efforts are currently underway toward improving harmonization and standardization of regulations and practices applicable to collection of DNA samples. However, it may be well into the future before there is consensus across nations. Because country-specific local and regional laws and regulations continually evolve, it is advisable to regularly verify these laws and regulations for the jurisdiction in which approval for DNA collection is being given.

Regulatory Authorities

The use of PGx information to improve the risk:benefit profile of drugs is increasingly being encouraged by regulatory health authorities. Authorities such as the FDA (USA), EMEA (European Union), MHLW (Japan), and ICH (International) are playing a key role in advancing this scientific field as it applies to pharmaceutical development. A significant number of regulatory guidances and concept papers have already been issued[1, 3, 7-18], and are available through: http://www.i-pwg.org. DNA sample collection has become a key component of clinical development. It is anticipated that regulatory authorities eventually may require relevant PGx data with drug submissions[19].

Where to Get More Information

Several expert organizations are helping to advance the adoption of PGx in clinical development and in medical care. A vast array of educational resources related to PGx that cater to health care professionals, IRBs/IECs, scientists, and patients have been created and are publicly available. Many of these organizations and resources are available through the I-PWG website: http://www.i-pwg.org.

What is the Industry Pharmacogenomics Working Group (I-PWG)?

The Industry Pharmacogenomics Working Group (I-PWG) (formerly the Pharmacogenetics Working Group) is a voluntary association of pharmaceutical companies engaged in PGx research. The Group's activities focus on non-competitive educational, informational, ethical, legal, and regulatory topics. The Group provides information and expert opinions on these topics and sponsors educational/informational programs to promote better understanding of PGx research for key stakeholders. The I-PWG interacts with regulatory authorities and policy groups to ensure alignment. More information about the I-PWG is available at: http://www.i-pwg.org.

Glossary

Identified Data and Samples: Identified data and samples are labeled with personal identifiers such as name or identification numbers (e.g., social security or national insurance number). The use of identified data and samples allows for clinical monitoring and subject follow-up and are generally not considered appropriate for purposes of clinical trials in drug development. (Not generally applicable to PGx in pharmaceutical clinical trials).

Coded Data and Samples: Coded data and samples are labeled with at least one specific code, and do not carry any personal identifiers.

Single-Coded Data and Samples: are usually labeled with a single specific code. It is possible to trace the data or samples back to a given individual with the use of a single coding key.

Double-Coded (De-Identified) Data and Samples: are initially labeled with a single specific code and do not carry any personal identifiers. The data and samples are then relabeled with a second code, which is linked to the first code via a second coding key. It is possible to trace the data or samples back to the individual by the use of both coding keys. The use of the second code provides additional confidentiality and privacy protection for subjects over the use of a single code.

Anonymized Data and Samples: Anonymized data and samples are initially single or double coded but the link between the subjects' identifiers and the unique code(s) is subsequently deleted. Once the link has been deleted, it is no longer possible to trace the data and samples back to individual subjects through the coding key(s). Anonymization is intended to prevent subject re-identification.

Anonymous Data and Samples: Anonymous data and samples are never labeled with personal identifiers when originally collected, nor is a coding key generated. Therefore, there is no potential to trace back genomic data and samples to individual subjects. Due to restrictions on the ability to correlate clinical data with such samples, they are generally of little use to PGx research. (Not generally applicable to PGx in pharmaceutical clinical trials).

6

References

1. ICH E15 - Definitions for Genomic Biomarkers, Pharmacogenomics, Pharmacogenetics, Genomic Data and Sample Coding Categories. April 2008. (Accessed at: http://www.fda.gov/OHRMS/DOCKETS/98fr/FDA-2008-D-0199-gdl.pdf and at: http://www.ich.org/LOB/media/MEDIA3383.pdf)

2. Anderson DC, Gomez-Mancilla B, Spear BB, et al. Elements of informed consent for pharmacogenetic research; perspective of the pharmacogenetics working group. Pharmacogenomics Journal 2002;2(5):284-92.

3. ICH E6(R1) - Guideline for Good Clinical Practice. June 1996. (Accessed at: http://www.ich.org/LOB/media/MEDIA482.pdf)

4. Renegar G, Webster CJ, Stuerzebecher S, et al. Returning genetic research results to individuals: points-to-consider. Bioethics 2006;20(1):24-36.

5. Genetic Information Nondiscrimination Act (GINA): 2007-2008. (Accessed at: http://www.genome.gov/24519851)

6. Hudson KL, Holohan MK, Collins FS. Keeping pace with the times--the Genetic Information Nondiscrimination Act of 2008. New England Journal of Medicine 2008;358(25):2661-3.

7. EMEA CHMP Reflection Paper on Pharmacogenomics in Oncology - Draft. 2008. (Accessed at: http://www.emea.europa.eu/pdfs/human/pharmacogenetics/12843506endraft.pdf)

8. EMEA CHMP Position Paper on Terminology in Pharmacogenetics. June 2003. (Accessed at: http://www.tga.health.gov.au/docs/pdf/euguide/emea/307001en.pdf)

9. EMEA CHMP Reflection Paper on the Use of Pharmacogenetics in the Pharmacokinetic Evaluation of Medicinal Products. May 2007. (Accessed at: http://www.emea.europa.eu/pdfs/human/pharmacogentics/12851706enfin.pdf)

10. EMEA CHMP Guideline on Pharmacogenetic Briefing Meetings. November 2006. (Accessed at: http://www.emea.europa.eu/pdfs/human/pharmacogenetics/2022704en.pdf)

I-PWG

11. EMEA CHMP Reflection Paper on Pharmacogenomic Samples, Testing, and Data Handling. November 2007. (Accessed at: http://www.emea.europa.eu/pdfs/human/pharmacogenetics/20191406en.pdf)

12. EMEA CHMP Reflection Paper on the Use of Genomics in Cardiovascular Clinical Intervention Trials. November 2007. (Accessed at: http://www.emea.europa.eu/pdfs/human/pharmacogenetics/27878906enfin.pdf)

13. EMEA CHMP Biomarkers Qualification: Guidance to Applicants. 2008. (Accessed at: http://www.emea.europa.eu/pdfs/human/biomarkers/7289408en.pdf)

14. EMEA CHMP Understanding Terminology Used in Pharmacogenetics July 2004. (Accessed at: http://www.emea.europa.eu/pdfs/human/pharmacogenetics/384204en.pdf)

15. FDA Companion Guidance - Pharmacogenomic Data Submissions - draft. August 2007. (Accessed at: http://www.fda.gov/downloads/Drugs/GuidanceComplianceRegulatoryInformation/Guidances/ucm079855.pdf)

16. FDA. FDA Guidance - Pharmacogenetic Tests and Genetic Tests for Heritable Markers. June 2007. (Accessed at: www.fda.gov/MedicalDevices/DeviceRegulationandGuidance/GuidanceDocuments/ucm077862.htm)

17. FDA Guidance - Pharmacogenomic Data Submissions. March 2005. (Accessed at: http://www.fda.gov/downloads/RegulatoryInformation/Guidances/UCM126957.pdf)

18. EMEA FDA - Processing Joint FDA EMEA VGDSs within the framework of the Confidentiality Arrangement May 2006. (Accessed at: http://www.emea.europa.eu/pdfs/general/direct/pr/FDAEMEA.pdf)

19. Rittenhouse P. Framing DNA collection in the clinic. Biocentury, The Bernstein Report on BioBusiness March 2008:A13-5.

Created by the Industry Pharmacogenomics Working Group Education Task Force
© 2008 Industry Pharmacogenomics Working Group
All rights reserved.

This document represents the current view of the I-PWG at the time of publishing. Views and company representation in the I-PWG may change over time. The content of this document should not be modified without prior consent of the I-PWG. Translations must also be reviewed by the I-PWG. Since local laws and regulations may change at any time, individuals should always ensure adherence to applicable laws, regulations, and guidelines.

7 I-PWG

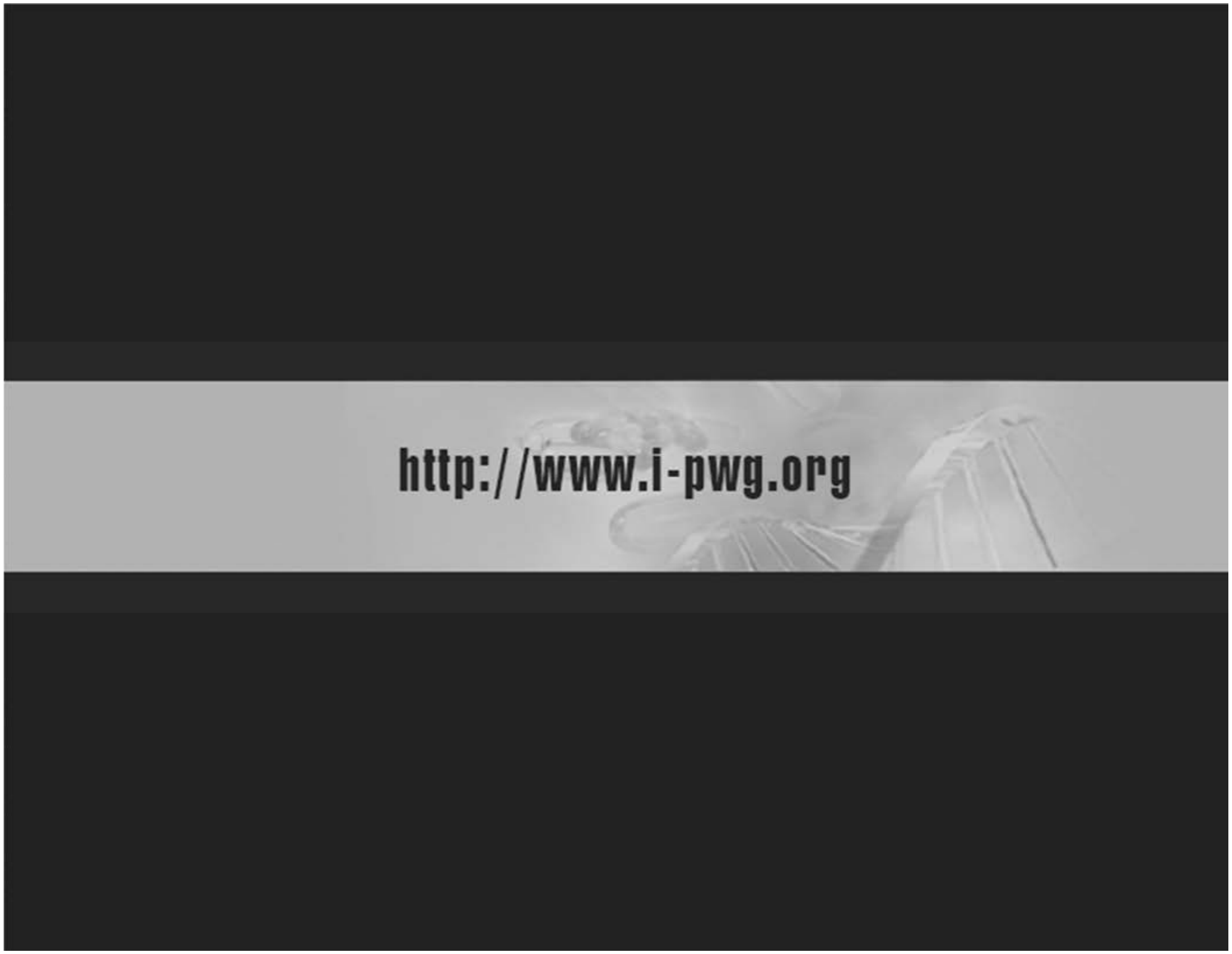
http://www.i-pwg.org

12.4 Approximate Blood/Tissue Volumes Drawn/Collected by Trial Visit and by Sample Types

Trial Visit	Screening	IV Study Treatment					Post-Treatment	
	V1 Screening	V2 Randomization	V3 OTX_1	V 4 OTX_2	V 5 OTX_3	V 6 EOT	V7 EFU	V8 Day 28
**Blood Parameter**	**Approximate Blood Volume (mL)**							
Hematology		2.0	2.0	2.0	2.0	2.0	2.0	2.0
Serum/Plasma Chemistry		5.0	5.0	5.0	5.0	5.0	5.0	5.0
Serum β-Human Chorionic Gonadotropin (β-hCG)[a]	3.5							3.5
Blood for Planned Genetic Analysis		8.5						
Serum/plasma for PK/PD evaluation	4.0	8.0	8.0	8.0				
**Expected Total (mL)[bc]**	**7.5**	**23.5**	**15.0**	**15.0**	**7.0**	**7.0**	**7.0**	**10.5**

a. For female subjects of child bearing potential only

b. Additional blood samples may be collected in support of evaluation for an underlying etiology throughout the study. The trial site guidance for assessment and follow up of these criteria can be found in the Investigator Trial File Binder. Depending on the results of initial testing, additional blood volumes could range from approximately 8.5 mL up to approximately 92.0 mL and could include HIV and/or Hepatitis testing.

c. Blood cultures will be collected in all subjects prior to initiation of IV study therapy. Subjects with positive blood cultures at screening should have follow-up blood cultures collected daily until 2 consecutive blood cultures demonstrate no growth. Depending on the results, additional blood volumes could range from approximately 40mL/patient (2 sets of blood cultures (10mL x 2 = 20mL)/aerobic culture; (10mL x 2 = 20mL)/anaerobic culture.) up to 560 mL. Blood cultures will be performed at the local microbiology laboratory according to recognized methods and per each laboratory's standard procedures.

12.5 APACHE II Severity of Disease Classification System – APACHE II Score Form

A. Acute Physiology Score:

	PHYSIOLOGIC VARIABLE	HIGH ABNORMAL RANGE					LOW ABNORMAL RANGE			
		+4	+3	+2	+1	0	+1	+2	+3	+4
1	Temperature rectal (^{o}C)	☐ ≥41	☐ 39-40.9		☐ 38.5-38.9	☐ 36.0-38.4	☐ 34-35.9	☐ 32-33.9	☐ 30-31.9	☐ ≤29.9
2	Mean arterial pressure = (2 x diastolic + systolic)/3	☐ ≥160	☐ 130-159	☐ 110-129		☐ 70-109		☐ 50-69		☐ ≤49
3	Heart rate (ventricular response)	☐ ≥180	☐ 140-179	☐ 110-139		☐ 70-109		☐ 55-69	☐ 40-54	☐ ≤39
4	Respiratory rate (nonventilated or ventilated)	☐ ≥50	☐ 35-49		☐ 25-34	☐ 12-24	☐ 10-11	☐ 6-9		☐ ≤5
5	Oxygenation A-aDO_2 or PaO_2 (mm Hg) a)FiO_2≥0.5:record A-aDO_2	☐ ≥500	☐ 350-499	☐ 200-349		☐ <200				
	b)FiO_2<0.5:record only PaO_2					☐ >70	☐ 61-70		☐ 55-60	☐ <55
6	Arterial pH (*If no ABGs record Serum HCO3 below)	☐ ≥7.7	☐ 7.6-7.69		☐ 7.5-7.59	☐ 7.33-7.49		☐ 7.25-7.32	☐ 7.15-7.24	☐ <7.15
7	Serum Sodium	☐ ≥180	☐ 160-179	☐ 155-159	☐ 150-154	☐ 130-149		☐ 120-129	☐ 111-119	☐ ≤110
8	Serum Potassium	☐ ≥7	☐ 6-6.9		☐ 5.5-5.9	☐ 3.5-5.4	☐ 3-3.4	☐ 2.5-2.9		☐ <2.5
9	Serum Creatinine (mg/dL) Double Point for acute renal failure	☐ ≥3.5	☐ 2-3.4	☐ 1.5-1.9		☐ 0.6-1.4		☐ <0.6		
10	Hematocrit (%)	☐ ≥60		☐ 50-59.9	☐ 46-49.9	☐ 30-45.9		☐ 20-29.9		☐ <20
11	White Blood Count	☐ ≥40		☐ 20-39.9	☐ 15-19.9	☐ 3-14.9		☐ 1-2.9		☐ <1
12	Glasgow Coma Scale Enter 15 minus actual GCS –see calculations in table below	15-GCS =								
A	Total Acute Physiology Score (APS)	Sum of the 12 individual variable points =								
*	Serum HCO3(venous-mMol/L) (Not preferred, use if no ABGs)	☐ ≥52	☐ 41-51.9		☐ 32-40.9	☐ 22-31.9		☐ 18-21.9	☐ 15-17.9	☐ <15

APACHE II Severity of Disease Classification System

Glasgow Coma Score (GCS)<br>(circle appropriate response)		
Eyes open (E)<br>4 - spontaneously<br>3 - to verbal command<br>2 - to painful stimul<br>1 - no response	Motor response (M)<br>6 - to verbal command<br>5 - localizes to pain<br>4 - withdraws to pain<br>3 - decorticate<br>2 - decerebrate<br>1 - no response	Verbal - Response (V)<br>5-oriented and controversed<br>4-confused and disoriented<br>3-inappropriate words<br>2-incomprehensible sounds<br>1-no response
GLASGOW COMA SCORE† = E + M + V		
†Subjects scoring 3 or 4 have an 85% chance of dying or remaining vegetative, while scores above 11 indicate 5 to 10% likelihood of death or vegetative state and 85% chance of moderate disability or good recovery. Intermediate scores correlate with proportional chances of subjects recovering.		
B. Age Points		
Age / Points<br>≤44 0<br>45-54 2<br>55-64 3<br>65-74 5<br>≥75 6<br>Age points = _________		
C. Chronic Health Points (CHE)		
If any of the 5 CHE categories is answered with yes give +5 points for nonoperative or emergency postoperative subjects, or +2 points for elective postoperative subjects<br>Liver - Cirrhosis with Portal Hypertension (PHT) or encephalopathy<br>Cardiovascular –NYHA Class IV angina at rest or with minimal self-care activities<br>Pulmonary -chronic hypoxemia or hypercapnia or polycythemia or PHT >40 mm Hg<br>Kidney -chronic peritoneal dialysis or hemodialysis<br>Immune -immune compromised host<br><br>Chronic Health Points=________		
APACHE-II Score is sum of A+B+C<br>APS points A _______<br>Age points +B _______<br>Chronic Health Points +C _________<br>Total APACHE-II Score=________		

Source: [20]

12.6 Clinical Pulmonary Infection Score (CPIS)

**PARAMETER**	**SCORE**
**Temperature (°C)**	
≥36.5 and ≤38.4	0
≥38.5 and ≤38.9	1
≥39.0 or ≤36.5	2
**White Blood Cell (WBC) Count (X 10^9/L)**	
≥4,000 and ≤11,000	0
<4,000 or >11,000	1
<4,000 or >11,000 & band forms ≥50%	2
**Lower Respiratory Tract Sample/Tracheal Secretions**	
No or minimal sputum/secretions	0
Non-purulent sputum/secretions	1
Purulent sputum/secretions	2
**PaO_2/FiO_2***	
Not ventilated to allow assessment	0
>240 or evidence of ARDS**/pulmonary contusion	0
≤240 and no evidence of ARDS*/pulmonary contusion	2
**Chest Radiograph at Study Entry**	
No infiltrate	0
Diffuse (or patchy) infiltrate	1
Localized infiltrate	2

* Ratio of partial pressure of arterial oxygen to the fraction of inspired oxygen.

** ARDS is defined as a PaO_2/FiO_2 ≤200, PAOP ≤18 mmHg, and acute bilateral infiltrates

ARDS = acute respiratory distress syndrome; PaO_2/FiO_2 = ratio of partial pressure of arterial oxygen to the fraction of inspired oxygen; PAOP = pulmonary artery occlusion pressure

Source: Modified from [21]

12.7 List of Abbreviations

Abbreviation	Definition
ABG	arterial blood gas
AC	alveolar cells
AE	adverse event
ALT	alanine aminotransferase
APACHE	acute physiology and chronic health evaluation
ARDS	acute respiratory distress syndrome
ASaT	All Subjects as Treated
AST	aspartate aminotransferase
ATS	American Thoracic Society
$AUC_{0-\infty}$	area under the concentration time curve
BAL	bronchoalveolar lavage
β-hCG	β-Human Chorionic Gonadotropin
BLI	β-lactamase inhibitor
CE	clinically evaluable
C-G	Cockcroft-Gault
CHMP	Committee for Medicinal Products for Human Use
cIAI	complicated intra-abdominal infection
CL_{plasma}	plasma clearance
CMS	colistimethate sodium
CMH	Cochran-Mantel-Haenszel
CPIS	Clinical Pulmonary Infection Score
CSR	clinical study report
cUTI	complicated urinary tract infection
DCIV	discontinuation of IV therapy
DMC	Data Monitoring Committee
DNA	deoxyribonucleic acid
ECI	event of clinical interest
eCRF	electronic case report form
eDMC	external Data Monitoring Committee
EFU	early follow-up
ELF	epithelial lung fluid
EMA	European Medicines Agency
EOC	Executive Oversight Committee
EOT	end of therapy
ESRD	end-stage renal disease
EU	European Union
FDA	Food and Drug Administration
GFR	glomerular filtration rate
HABP	hospital-acquired bacterial pneumonia
ICF	Informed Consent Form
HIV	human immunodeficiency virus
IB	Investigator's Brochure
ICH	International Conference on Harmonisation
ICU	intensive care unit
IDSA	Infectious Diseases Society of America
IMI	imipenem/cilastatin

Abbreviation	Definition
Imipenem-R	imipenem-resistant
INR	international normalized ratio
IRB/ERC	Institutional Review Board/Ethics Review Committee
IU	international units
IV	intravenous (parental)
IVRS/IWRS	interactive voice response system/integrated web response system
KPC	*Klebsiella pneumoniae* carbapenemase
LOS	length of stay
MAOI	monoamine oxidase inhibitors
MDR	multi-drug resistant
ME	microbiologically-evaluable
Mg	milligram
MITT	modified intention-to-treat
mMITT	microbiological modified intention-to-treat
MRSA	methicillin-resistant *Staphylococcus aureus*
MSCC	mid-stream clean catch
MSSA	methicillin-susceptible *Staphylococcus aureus*
NI	non-inferior
OTX	on-therapy visit
PaO_2/FiO_2	partial pressure of oxygen to the fraction of inspired oxygen
PD	pharmacodynamic
PIP	piperacillin
PGt	pharmacogenetic
PK	pharmacokinetic
q6h	every 6 hours
q12h	every 12 hours
REL	relebactam
SAE	serious adverse event
SAP	statistical analysis plan
siDMC	standing internal Data Management Committee
sSAP	supplemental Statistical Analysis Plan
TAH	total abdominal hysterectomy
TAZ	tazobactam
$t_{1/2}$	terminal half-life
ULN	upper limit of normal
US	United States
VABP	ventilator-associated bacterial pneumonia
WBC	white blood cell

13.0 SIGNATURES

13.1 Sponsor's Representative

TYPED NAME	
TITLE	
SIGNATURE	
DATE SIGNED	

13.2 Investigator

I agree to conduct this clinical trial in accordance with the design outlined in this protocol and to abide by all provisions of this protocol (including other manuals and documents referenced from this protocol). I agree to conduct the trial in accordance with generally accepted standards of Good Clinical Practice. I also agree to report all information or data in accordance with the protocol and, in particular, I agree to report any serious adverse events as defined in Section 7.0 – Assessing and Recording Adverse Events. I also agree to handle all clinical supplies provided by the Sponsor and collect and handle all clinical specimens in accordance with the protocol. I understand that information that identifies me will be used and disclosed as described in the protocol, and that such information may be transferred to countries that do not have laws protecting such information. Since the information in this protocol and the referenced Investigator's Brochure is confidential, I understand that its disclosure to any third parties, other than those involved in approval, supervision, or conduct of the trial is prohibited. I will ensure that the necessary precautions are taken to protect such information from loss, inadvertent disclosure or access by third parties.

TYPED NAME	
TITLE	
SIGNATURE	
DATE SIGNED	
